# Acid/Base-Triggered Photophysical and Chiroptical Switching in a Series of Helicenoid Compounds

**DOI:** 10.3390/molecules28217322

**Published:** 2023-10-29

**Authors:** Laure Guy, Maëlle Mosser, Delphine Pitrat, Jean-Christophe Mulatier, Mercedes Kukułka, Monika Srebro-Hooper, Erwann Jeanneau, Amina Bensalah-Ledoux, Bruno Baguenard, Stéphan Guy

**Affiliations:** 1 Laboratoire de Chimie UMR 5182, Université Lyon, ENS de Lyon, CNRS, F-69342 Lyon, France; maelle.mosser@orange.fr (M.M.); delphine.pitrat@ens-lyon.fr (D.P.); jean-christophe.mulatier@ens-lyon.fr (J.-C.M.); 2 Faculty of Chemistry, Jagiellonian University, 30-387 Krakow, Poland; 3 Centre de Diffractométrie Henri Longchambon, Université Claude Bernard Lyon 1, 5 Rue de la Doua, F-69100 Villeurbanne, France; erwann.jeanneau@univ-lyon1.fr; 4 Institut Lumière Matière UMR 5306, Université Lyon, CNRS, F-69622 Villeurbanne, France; amina.bensalah-ledoux@univ-lyon1.fr (A.B.-L.); bruno.baguenard@univ-lyon1.fr (B.B.); stephan.guy@univ-lyon1.fr (S.G.)

**Keywords:** dibenzo[c]acridine derivatives, chiroptical properties, ECD, CPL, acid/base equilibrium, (TD)DFT, chiral switch

## Abstract

A series of molecules that possess two quinolines, benzoquinolines, or phenanthrolines connected in a chiral fashion by a biaryl junction along with their water-soluble derivatives was developed and characterized. The influence of the structure on the basicity of the nitrogen atoms in two heterocycles was examined and the photophysical and chiroptical switching activity of the compounds upon protonation was studied both experimentally and computationally. The results demonstrated that changes in the electronic structure of the protonated vs. neutral species, promoting a bathochromic shift of dominant electronic transitions and alternation of their character from π-to-π* to charge-transfer-type, when additionally accompanied by the high structural flexibility of a system, leading to changes in conformational preferences upon proton binding, produce particularly pronounced modifications of the spectral properties in acidic medium. The latter combined with reversibility of the read-out make some of the molecules in this series very promising multifunctional pH probes.

## 1. Introduction

The concentration of hydrogen ions, commonly expressed using the pH scale, is an important parameter in numerous (bio)chemical reactions and physiological processes affecting, for example, ion transport along with cell growth and proliferation [1]. Abnormal pH values may thus give rise to cell dysfunction and, in consequence, to such conditions as heart stroke, Alzheimer’s disease, or cancer [1,2,3]. Therefore, quantitative evaluation of pH changes in environmental and biochemical analyses appears vital in monitoring and understanding these pathological processes, and increasingly in ensuring proper medical diagnosis and treatment evaluation. To date, a number of methods have been developed to measure pH, including electrochemical techniques using electrodes and spectroscopic approaches based on nuclear magnetic resonance (NMR), optical activity, absorption spectroscopy, and fluorescent probes [4,5,6,7]. Recently, extensive efforts have been put into proposing methods to monitor pH changes in situ [8,9,10]. Of all the reported approaches, those based on fluorescence measurements attract considerable attention due to their noninvasiveness, rapid response time, good sensitivity, and high specificity. Until now, several families of fluorescent probes have been developed [11], but their optimization is still a subject of active research, particularly in certain pH ranges or detection wavelengths. Although circularly polarized luminescence (CPL) is sensitive to chirality, a property that is very common in biological environments, it is rarely considered a workable method for replacing unpolarized luminescence, due to typically weak CPL signals for organic molecules. The quest for methods to enhance their intensity, for example by using plasmonics or strain effects in two-dimensional materials, is thus still very active [12,13,14]. However, significant achievements have been reported for chiroptical switches responding to external stimuli such as light, temperature, mechanical stress, or chemical environment [15,16]. Among them, those based on a helicene skeleton, due to its intense chiroptical properties, have shown easy read-out of the protonation state [17,18,19].

**Figure 1:**
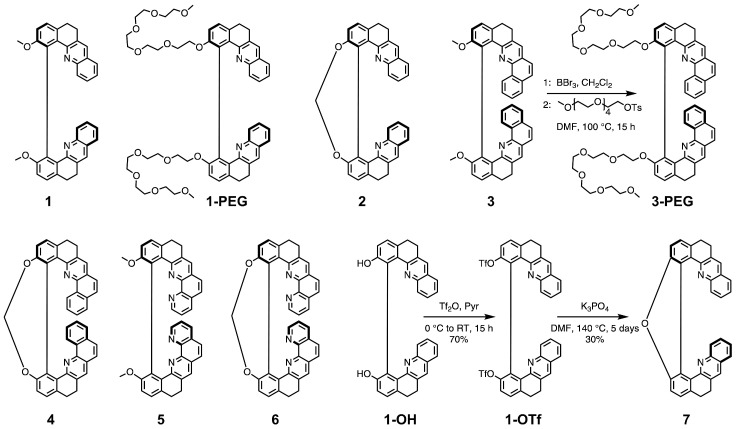
Structure (in (+)-stereoisomeric form in each case) of the *open* (non-constrained) and *closed* (constrained) helical molecules studied in this work along with synthetic pathways for the newly developed derivatives **3-PEG** and **7**.

Our laboratory has mastered a synthesis of chiral helicene-like systems that exhibit optical activity strongly depended on conformational changes [20]. These compounds present intense chiroptical signals compared to those reported for other small molecules [21] and possess nitrogen atoms constituting basic sites in their skeleton that make them promising potential pH probes readable by chiroptical techniques. Herein, we report on the protonation-triggered photophysical and chiroptical switching properties of seven such helicenoid molecules varying in the number of rings and flexibility of the structure (Figure 1), which have been investigated both experimentally and via quantum-chemical calculations. Moreover, since pH measurements are related to aqueous solutions, we also present newly developed water-soluble derivatives of some of these systems.

## 2. Results and Discussion

### 2.1. Studied Systems

Syntheses of the molecules **1**–**6** considered in this study (Figure 1) have been reported elsewhere [20,22]. We showed that these two series of non-constrained (*open*) 8-/10-ring and constrained (*closed*) 9-/11-ring helically-shaped compounds can be obtained as pure enantiomers on a gram scale from a key chiral intermediate [23]. Recently, a water-soluble PEGylated (PEG = polyethylene glycol) derivative of **1**, labelled in the following as **1-PEG**, has been proposed [24]. Herein, we introduce its analogue, a water-soluble derivative of **3**, **3-PEG**. **1-PEG** and **3-PEG** were obtained in 69% and 53% yields, respectively, for (+)-**1-PEG** and (−)-**1-PEG**, or 69% and 67% for (+)-**3-PEG** and (−)-**3-PEG**, by successive demethoxylation of **1** or **3** using boron tribromide (BBr${}_{3}$) and phenol nucleophilic substitution with 2,5,8,11,14-pentaoxahexadecan-16-yl 4-methylbenzene-1-sulfonate (Figure 1). We also present the synthesis of a new *closed* compound **7** bridged by a furan ring. It has been unexpectedly obtained for the first time as the only product in our attempts to synthesize a bis-boronate derivative of **1** by Miyaura borylation reaction using bis(diphenylphosphino)ferrocene dichloropalladium(II) (PdCl${}_{2}$(dppf)) as a catalyst under basic conditions. Similarly, intramolecular O-arylation ring closure instead of cross-coupling of bis(pinacolato)diboron (B${}_{2}$pin${}_{2}$) with bis-triflated precursors has already been reported, and the proposed pathway involves (i) partial hydrolysis of one of the triflates by a trace amount of water in the solvent and (ii) intramolecular aromatic nucleophilic substitution that leads to the formation of the oxygen bridge [25,26]. In an attempt to optimize the yield of **7** by varying the type of catalyst, PdCl${}_{2}$(dppf) or Pd(PPh${}_{3}$)${}_{4}$ and dicyclohexyl(2’-methyl-[1,1’-biphenyl]-2-yl)phosphine or Pd${}_{2}$(dba)${}_{3}$ (dba = dibenzylideneacetone) and 9,9-dimethyl-4,5-bis(diphenylphosphino)xanthene (Xanphos), temperature, and reaction time, we ultimately found that the best results are obtained without a catalyst. Finally, (±)-**1-OTf** [24] is heated at 140 ${}^{\circ }$C for 5 days in the presence of two equivalents of K${}_{3}$PO${}_{4}$, yielding 30% of (±)-**7** (Figure 1). Such conditions are not compatible with the synthesis of **7** as pure enantiomers due to possible racemisation of **1-OTf**. Subsequently, the preparative resolution by chiral HPLC was performed (see the Supplementary Materials) and gave both enantiomers of **7** with an enantiomeric excess higher than 99.5%.

**Figure 2:**
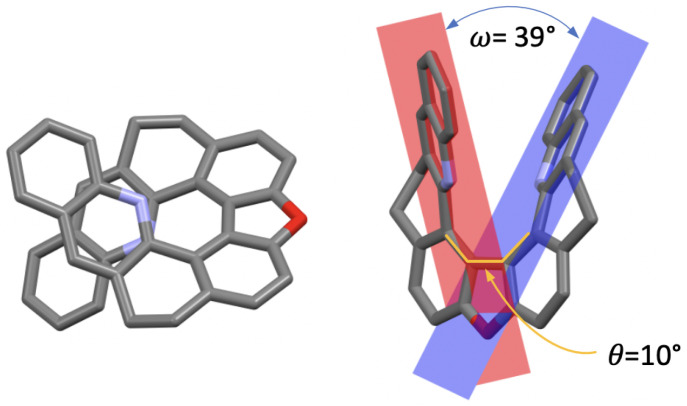
X-ray crystal structure of (±)-**7** (only the (−)-**7** stereoisomer is shown) presented in two orientations. Hydrogen atoms are omitted for clarity; C, O, and N atoms are marked in grey, red, and blue, respectively. θ: biphenyl’s torsional angle, ω: angle between the two benzo[c]acridine subunits’ planes.

The X-ray analysis of monocrystals of (±)-**7**, obtained by slow evaporation, enabled us to make a comparison of its solid-state conformation with that of the other compounds in the series, focusing on two parameters: the central biphenyl’s torsional angle, θ, and the angle between two benzo[c]acridine moieties’ planes, ω (Figure 2). In the case of the *open* structures **1**, **3**, and **5**, θ ranges from 115${}^{\circ }$ in **3** to 64${}^{\circ }$ in **5**, while for their corresponding *closed* derivatives **2**, **4**, and **6**, θ values, fixed by the nature of the seven-member ring of 1,3-dioxepin in its C${}_{2}$ conformation, do not vary as much and are close to 50${}^{\circ }$ [20,27]. Regarding **7**, the closure of the five-member ring results in an almost flat dibenzofuran motif with the consequently greatly diminished torsional angle θ of 10${}^{\circ }$. As far as the ω angle is concerned, its value, related to the helical pitch of the structure, ranges from 62${}^{\circ }$ in **3** to 7${}^{\circ }$ in **5** [20,22], with **7** demonstrating a value of 39${}^{\circ }$, which is clearly increased compared to its 1,3-dioxepin-based analogue **2** (24${}^{\circ }$). While both molecules demonstrate some flexibility of the backbone provided by the saturated -CH${}_{2}$-CH${}_{2}$- bridges that allow the helix to distort, the presence of the dibenzofuran motif in **7** visibly limits the interaction between the two terminal rings in helically arranged structures.

### 2.2. Basicity Constants Measurements

**Figure 3:**
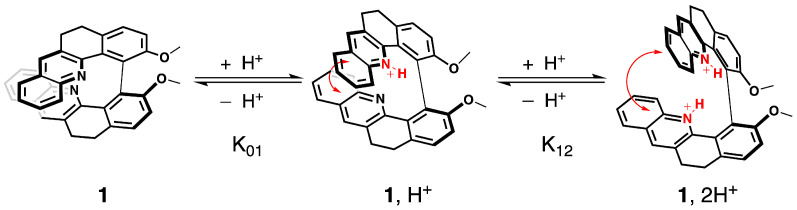
Electronic and structural states of **1** associated with the two acid/base equilibria and the corresponding chemical constants.

We have already evidenced that the spectroscopic properties of the molecules in the series of **1**–**6** are strongly affected by their electronic and conformational structure [20]. In this study, we postulate that protonation will modulate spectroscopic fingerprints in a similar way. Namely, after the binding of a proton to the pyridinic nitrogen atoms, the inductive attractor effect of the pyridinium will disturb the electronic distribution along the skeleton of the molecule, while repulsion between the bis-pyridinium positive charges is expected to change the helical pitch of the structure and thus the conformational preferences, as illustrated for **1** in Figure 3 (vide infra).

**Figure 4:**
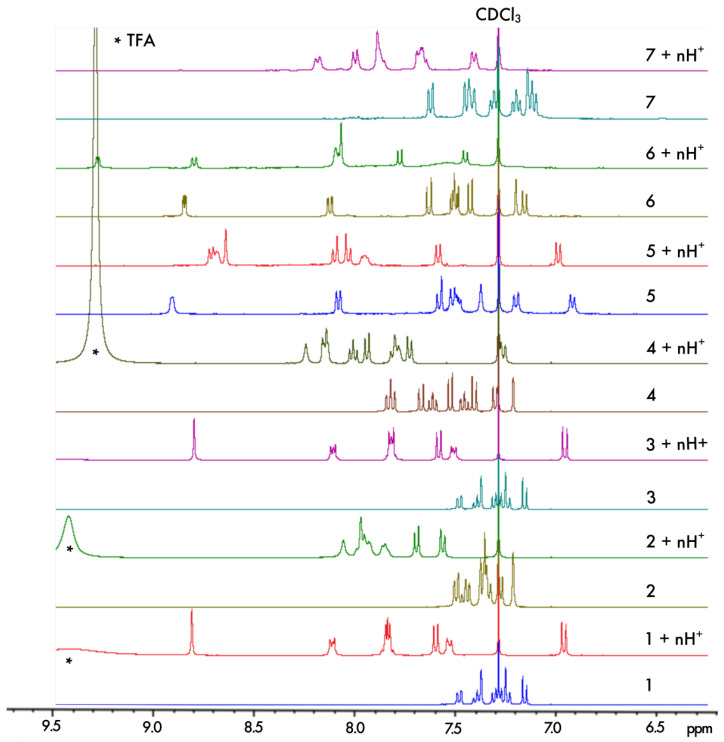
Comparison of the chemical shifts in the aromatic region of the ${}^{1}$H NMR spectra for **1**–**7** in CDCl${}_{3}$ and upon their protonation in CDCl${}_{3}$ with an excess of TFA (indicated by * in the spectra) ensuring n ≫ 2.

**Figure 5:**
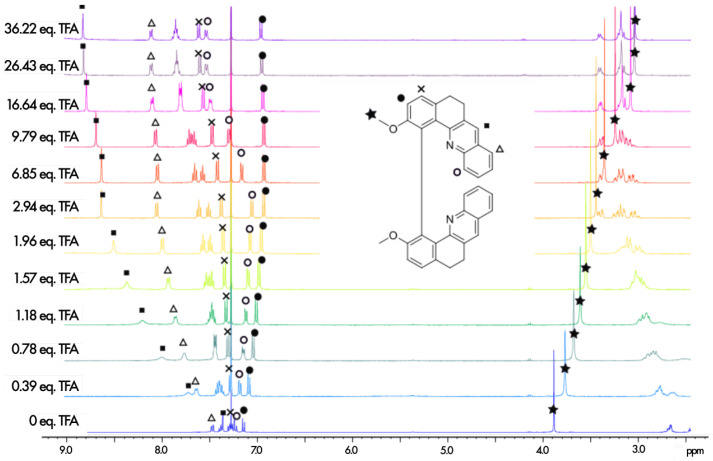
${}^{1}$H NMR spectra recorded during the titration of **1** with TFA (400 MHz, 298 K, CDCl${}_{3}$). The assignment of the full neutral spectrum was unambiguously performed by 2D NMR.

The protonation process was monitored using ${}^{1}$H NMR spectroscopy. The aromatic regions of the spectra for **1**–**7** in pure CDCl${}_{3}$ and in CDCl${}_{3}$ with an excess of trifluoroacetic acid (TFA) are presented in Figure 4. As can be seen, except for **5**, complete protonation results in an enlargement of the aromatic region with a downfield or an upfield of chemical shifts by several ppm for the majority of the protons. Furthermore, titration with TFA allows us to determine the values of two proton-binding constants, K${}_{01}$ and K${}_{12}$, for both acid/base equilibria, presented schematically in Figure 3, using the fact that consistently, along the series, a rapid exchange on the NMR time scale is observed for these equilibria according to the average chemical shifts of the species upon addition of the titrant. In particular, as shown for **1** in Figure 5, the chemical shift of the proton at the β position to the aromatic nitrogen atom (marked by an empty circle) is first upfielded until less than three equivalents of TFA are added, and then it is shifted to the lower field. The equilibrium constants, along with the corresponding pKa values for the two considered protonation equilibria for all the considered systems, are presented in Table 1.

As can be seen in Table 1, the values of the equilibrium constants determined for the first protonation for **1**–**6** are significantly higher compared to those for the second protonation, which is due to the presence of the first positive charge in the molecule. It is worth noting that pKa values are measured in deuterated chloroform, a solvent less polar than water, so it is not surprising that they are lower compared to, for example, pKa = 5.2 in water or pKa = 3.4 in DMSO for the pyridinium/pyridine couple. Furthermore, while pyridine has a higher pKa than phenanthroline in water (5.2 vs. 4.8), **5**, which contains two phenanthroline moieties in its skeleton, exhibits a very high pKa${}_{1}$ value (4.7) in CDCl${}_{3}$, almost twice that of the bis-pyridine derivative **1** (2.6). This, along with the lowest pKa${}_{2}$ compared to the other studied systems, seems to stem from an exceptional stabilization of the mono-protonated species of **5** (vide infra). The second trend that emerges from the data is that both pKa values of the *closed* structures are at least approximately 1–2 times lower than those of the corresponding *open* systems. This loss in basicity probably arises from the rigidity of the structure and thus the lower accessibility of the nitrogen doublets. The behaviour of **7** upon its first protonation is consistent with the other *closed* molecules with pKa${}_{1}$ < 2 but, interestingly, this is the only compound exhibiting pKa${}_{2}$ > pKa${}_{1}$ that clearly reflects its different—compared especially to the remaining *closed* systems—smaller and more rigid structure of the bridging central ring.

**Figure 6:**
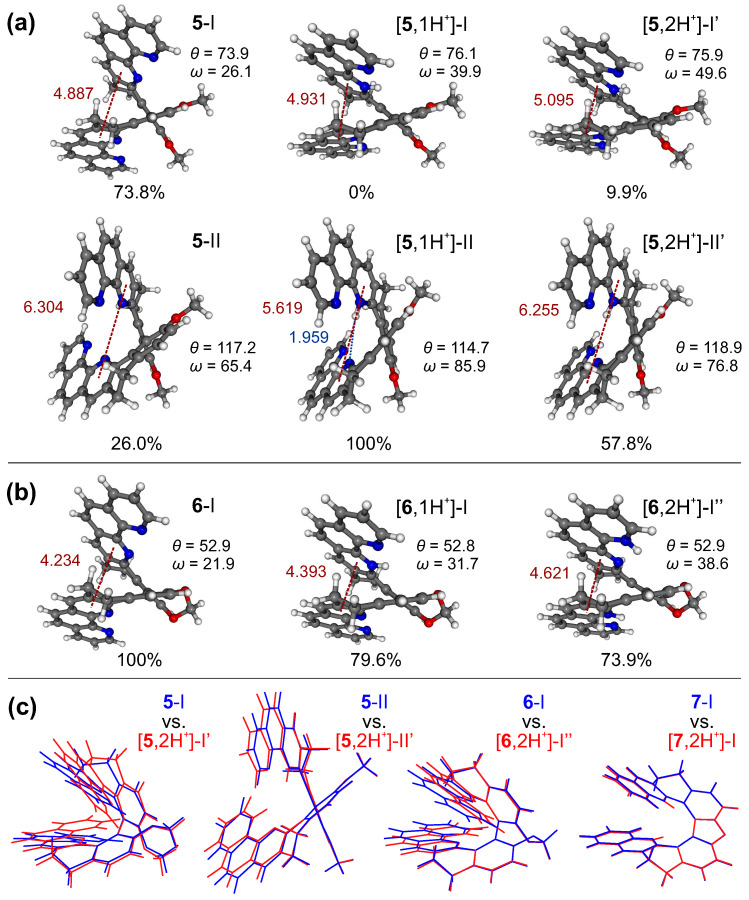
The lowest-energy optimized structures of the neutral [20], mono-, and double-protonated species of **5** (**a**) and **6** (**b**) in the ground state. Numbers listed are the Boltzmann populations at 298 K, distances (in Å) between the inner pyridine ring centroids of each 1,10-phenanthroline subunit (in red), biphenyl’s torsional angles, and angles between two aromatic subunits’ planes (θ and ω, respectively, in ${}^{\circ }$, compared with Figure 2). The length (in Å) of the hydrogen bonding formed between two subunits in [**5**,1H${}^{+}$]-II is also provided (in blue). (**c**) Overlays of the optimized structures of the most populated conformers of **5**, **6**, and **7** in their neutral (blue) and double-protonated (red) forms. BLYP/TZVP/COSMO(CH${}_{3}$CN) calculations.

To shed light on the effects of protonation processes on energetic and structural preferences for the systems in the examined series, density functional theory (DFT) geometry optimizations of the molecular structures of **1**–**7** in their mono- and double-protonated forms were performed, considering conformers varied in the folding of the helical skeleton and the relative orientation of the -CH${}_{2}$-CH${}_{2}$- bridges, and, where applicable, also in the position of the attached proton(s). The obtained results were analyzed and compared to those for the respective neutral species [20]. The illustrative examples are presented in Figure 6, while a full set of computed data can be found in Figures S2.1–S2.4 in the Supplementary Materials.

The calculations indeed confirm that proton binding generally leads to an increase in the distance between aromatic rings in the case of the structures with their parallel-like (helicene-like) arrangement, such as those for the *closed* compounds (see **6** in Figure 6b) and those corresponding to conformer I of the *open* systems (see **5** in Figure 6a), which is also reflected in the increased values of the angle between the planes formed by two aromatic subunits, ω. Furthermore, for the *open* compounds, as expected taking into account their higher flexibility, a visible increase in energetic preference/population of conformers with a greater helical pitch (as conformer II with a more perpendicular-like arrangement of the aromatic rings (see **5** in Figure 6a) was observed. Indeed, adopting such a structure ensures the most favourable positions of two repulsive bis-pyridinium positive charges in the double-protonated species, while in the case of mono-protonated ones it provides a stabilization of the introduced proton by intramolecular hydrogen bonding with the nitrogen atom(s) in the heterocycle of the opposite subunit. This additional stabilization of the mono-protonated conformers possible for the *open* compounds but not for the *closed* ones may account for the higher values of pKa observed for the former vs. latter systems. As for **5**, the stabilization of the first introduced proton appears to be particularly effective, due to the possibility of the formation of the intramolecular N⋯H hydrogen-bonding interaction of the pyridinium hydrogen atom, not only with the pyridine nitrogen of the opposite phenanthroline moiety but also with the adjacent nitrogen atom (located on the second pyridine ring of the same phenanthroline fragment); this compound, as aforementioned, demonstrates the highest pKa${}_{1}$ and the lowest pKa${}_{2}$ values.

### 2.3. Photophysical and Chiroptical Properties and Their Protonation-Triggered Switching

UV-vis absorption, fluorescence, electronic circular dichroism (ECD), CPL, and optical rotatory dispersion (ORD) spectra for **1**–**6** have been previously reported in ref. [20]. The chiroptical properties at neutral pH for this series of molecules were found to be closely related to the rigidification of the molecular structure. Namely, the *closed* compounds exhibit an intense and opposite-sign ECD signal in the low-energy region, strongly enhanced optical rotation (OR) parameters, and very strong CPL activity compared to their *open* precursors, as explained by their extended π-conjugation and lower conformational flexibility. The intense CPL signals of molecules **4** and **6** with dissymmetry factors up to $g_{lum}=8\times 10^{-3}$ are among the highest reported when considering (small) organic molecules [21,30,31].

**Figure 7:**
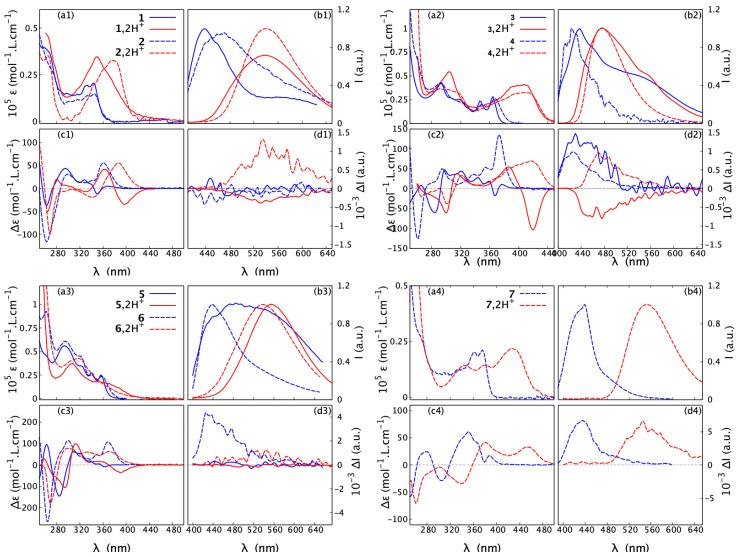
Absorption (**a1**–**a4**) and ECD (**c1**–**c4**) spectra, recorded in CH${}_{3}$CN at a typical concentration of $5\times 10^{-5}$ mol/L, along with fluorescence (**b1**–**b4**) and CPL (**d1**–**d4**), measured in CH${}_{2}$Cl${}_{2}$ at a typical concentration of 10${}^{-3}$ mol/L, for **1**–**7** (*open*: **1**, **3**, **5**; *closed*: **2**, **4**, **6**, **7**) in their neutral and protonated forms. Blue solid and dash lines represent, respectively, *open* and *closed* structures in neutral medium, while red solid and dash lines correspond to *open* and *closed* structures in acidic medium. For clarity, only spectra for the (+)-enantiomers are presented. For the corresponding results for the (-)-enantiomers, see the Supplementary Materials. For detailed measurement conditions, see Section 4.

The UV-vis absorption and ECD spectra of the newly demonstrated compound **7** in acetonitrile are shown in Figure 7 (bottom right panel, blue dash lines). Compared to other systems in the series (see Figure S2.5 in the Supplementary Materials), a bathochromic shift (red-shift) of up to ca. 30 nm of the lower-energy bands in both of these spectra can be observed. As shown in Figure S2.5, this shift is correctly reproduced by time-dependent DFT (TDDFT) calculations. As demonstrated in ref. [20], the lowest-energy absorption intensity for the *closed* compounds **2**, **4**, and **6** is associated with the lowest-energy excitation corresponding to a π-to-π*-type transition that involves the highest occupied (HO) and lowest unoccupied (LU) molecular orbitals (MOs), both almost uniformly delocalized over the π-electron system of the whole molecular structure. In the case of **7**, the underlying lowest-energy excitation originates from the HOMO-1-to-LUMO transition that also shows a π-to-π* character, but it is clearly predominantly localized in the central part of the molecule, i.e., the aromatic dibenzofuran bridge. The increased π-conjugation within the bridge observed in **7** compared to the 1,3-dioxepin-based systems also has a profound impact on orbital energies. In particular, it leads to a substantial stabilization of the LUMO level, making the HOMO-1/LUMO energy gap smaller than the corresponding HOMO/LUMO gaps in other *closed* compounds (see Figure S2.6 in the Supplementary Materials), which may rationalize the observed red-shift in the low-energy band of the UV-vis and ECD spectra of **7**.

The fluorescence spectrum of **7** shows a broad band centred around 440 nm, corresponding to the smallest Stokes shift among this series. More interestingly, with only nine rings in its structure, **7** exhibits an intense CPL response and its $g_{lum}$ reaches 10${}^{-2}$, a higher value than those for the previously reported 11-ring *closed* compounds **4** and **6**. Although the TDDFT-computed $g_{lum}$ values for **4**, **6**, and **7** appear clearly overestimated with respect to the experimental ones (especially for **4** and **6**), the aforementioned trend is, however, correctly reproduced (see Table S2.1 in the Supplementary Materials). In each case, the overall electronic character of the emitting $S_{1}$ state is rather similar and can be assigned as ππ* delocalized across almost the whole molecular structure with visible involvement of the lone pair of the nitrogen atoms that induces the nπ* component. The latter appears to be diminished in the case of **7**, for which, as in the ground state, more pronounced localization of the π-electron density in the central part of the molecule (dibenzofuran bridge) was additionally observed for HOMO and LUMO (see Figure S2.7 in the Supplementary Materials). Detailed analysis of the computed $g_{lum}=4R/D$ values (*R*/*D* is rotatory/dipole strength of the $S_{1}\to S_{0}$ emission transition) revealed that the increase in the dissymmetry factor observed for **7** vs. **4** and **6** might originate from an increase in the *R* value (see Table S2.2 in the Supplementary Materials). As *R* is a function of the magnitudes of the underlying electric |d| and magnetic |m| transition dipole moments and the cosinus of the angle φ between their vectors, R=|d|·|m|·cos(φ), this enhancement, on the other hand, can be traced back to an increase in |m| (Table S2.2), meaning that the $S_{1}\to S_{0}$ transition in **7** is more magnetically allowed [32].

**Figure 8:**
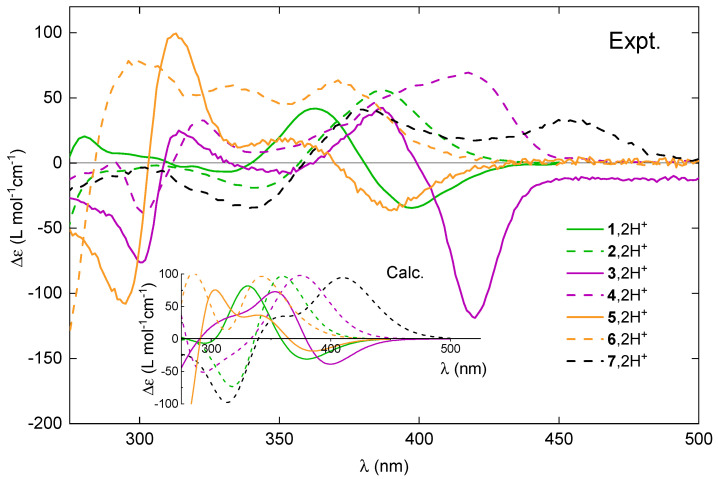
Comparison of the experimental ECD spectra for (+)-enantiomers of **1**–**7** upon their protonation. Inset: The corresponding TDDFT-simulated Boltzmann-averaged ECD spectra. No spectral shifts were applied. LC-PBE0*/SV(P)/PCM(CH${}_{3}$CN) calculations.

In our previous article [20], we concluded that the photophysical and chiroptical properties within the series of helicenoid compounds **1**–**6** are very sensitive to both the electronic structure of the system and its molecular flexibility. This study focuses on how those spectral features can be modulated by an external stimulus: the acidity of the medium. The influence of protonation of the examined *open* and *closed* compounds on their absorption, ECD, fluorescence, and CPL spectra (blue lines for pH-neutral conditions and red lines for pH-acidic conditions) is shown in Figure 7. Additionally, in Figure 8, a direct comparison of the ECD envelopes measured (and computed) for all the systems in their protonated forms is presented. These spectra were recorded in solutions containing a large excess of acid (10${}^{4}$ equivalents) in order to totally displace the equilibrium of the protonation process towards the double-protonated species.

As a general trend, upon protonation, the low-energy part of the UV-vis absorption spectra of **1**–**7** becomes broadened and red-shifted. The bathochromic shift of the lowest-energy band maximum for the *open* structures ranges from $\Delta \lambda_{abs}^{max}\left(1\right)=2$ nm to $\Delta \lambda_{abs}^{max}\left(3\right)=43$ nm. In comparison, the corresponding red-shift for the *closed* systems is larger but varies within a smaller range, between $\Delta \lambda_{abs}^{max}\left(2\right)=40$ nm and $\Delta \lambda_{abs}^{max}\left(7\right)=54$ nm. An exception that does not follow the aforementioned trend is compound **6**, which instead shows a hypsochromic shift (blue-shift) of 40 nm for the maximum of the lowest-energy UV-vis band upon proton binding. Nevertheless, consistent with what has been observed for other systems, the absorption spectrum of **6** in the acidic medium displays non-zero intensity at longer wavelengths than under the neutral conditions. Accordingly, for all the examined systems, the bathochromism upon protonation also occurs in the emission spectra, with shifts ranging from 40 nm for **3** to 114 nm for **7**.

**Figure 9:**
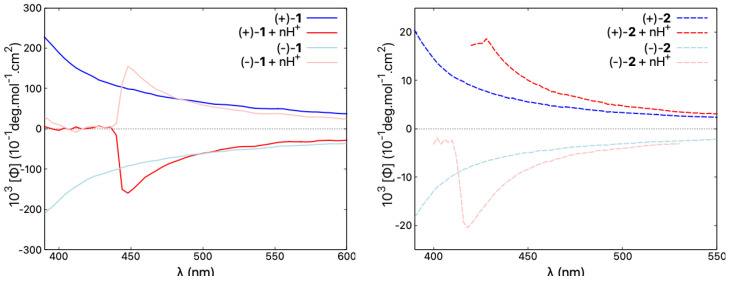
Comparison of ORD spectra for both enantiomers of **1** and **2**, representative of *open* and *closed* compounds, respectively, recorded in CH${}_{2}$Cl${}_{2}$ and in CH${}_{2}$Cl${}_{2}$ with an excess of TFA ensuring n ≫ 2, at a typical concentration of $5\times 10^{-3}$ mol/L. Blue/red lines correspond to neutral/acidic medium. See the Supplementary Materials for the corresponding ORD results for other molecules in the series.

Regarding ECD, the red-shift of the overall spectra for the protonated vs. neutral species of the *open* compounds is accompanied at the lowest-energy spectral range by either an increase in the negative intensity (in the case of (+)-**5**) or a sign inversion of the ECD intensity from (weakly) positive to strongly negative (in the case of (+)-**1** and (+)-**3**), so that, for all of these systems upon protonation, the lowest-energy band is negative. At the same time, as shown in Figure 9 and Figures S1.1–S1.5 in the Supplementary Materials, the ORD plots obtained in acidic medium, comparing to the one measured under neutral conditions, present the alternation of the sign for the entire low-energy spectral range that was examined. Interestingly, for all of the *open* molecules, the protonation thus has a clear signature: the intense negative ECD band appears in a spectral region, in which the corresponding neutral species presents no absorption. In comparison, the lower-energy part of the ECD spectra for the protonated (+)-enantiomers of the *closed* systems **2**, **4**, **6**, and **7** demonstrates bands with the same (positive) sign and similar intensity, but they are red-shifted and broadened compared to their neutral parent species. No sign inversion of the lowest-energy ECD band is further reflected by the same sign pattern of the corresponding ORD plots recorded under neutral and acidic conditions (see Figure 9 and Figures S1.1–S1.5 in the Supplementary Materials).

**Figure 10:**
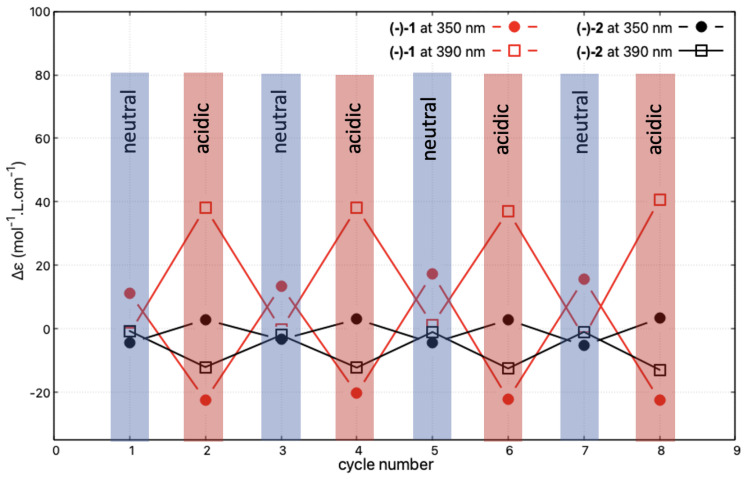
Reversibility of the protonation/deprotonation-triggered switching of the ECD signal at 350 and 390 nm for (-)-**1** and (-)-**2** (in CH${}_{3}$CN, at *c* = $3.4\times 10^{-5}$ mol/L and *c* = $4.2\times 10^{-5}$ mol/L, respectively) after successive changes in the character (neutral/acidic) of the medium. Four cycles of acidification (using sulphuric acid)/basification (using sodium hydroxide) have been performed, i.e., eight ECD spectra have been measured. The corresponding full ECD spectra obtained upon the protonation/deprotonation cycles are presented in Figures S1.6–S1.7 in the Supplementary Materials.

Reversibility and reproducibility of the modifications of the ECD signal upon successive protonation and deprotonation processes, reflecting the acid/base-triggered switching capacity of the studied systems, was then examined for (−)-**1** and (−)-**2**, representing, respectively, the *open* and *closed* compounds. The spectra for the neutral species were recorded after the deprotonation step, which was carried out by placing 3 mL of the acidic acetonitrile solution of the given system in contact with a sodium hydroxide pellet. The collected solution was subsequently acidified by the addition of 1 μL of sulphuric acid. Figure 10 shows the corresponding changes in the intensity of the ECD signals measured at 350 and 390 nm during four successive protonation/deprotonation cycles. The read-out of the protonated state is made very easy for (−)-**1** at these two wavelengths. Indeed, at 350 nm, the switch consists of a sign inversion of the signal, while at 390 nm the signal is turned on and off depending on the conditions. Exactly the same behaviour can also be observed for (−)-**2**, but it is associated with approximately one order of magnitude lower amplitude of the intensity changes.

**Figure 11:**
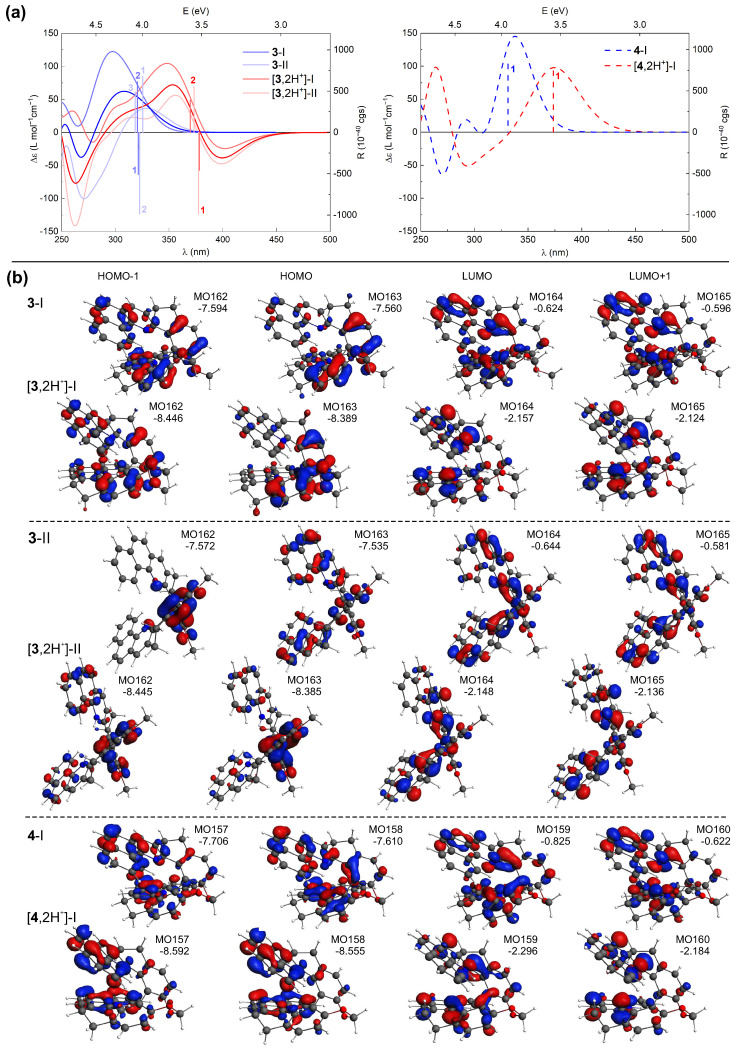
(**a**) TDDFT-simulated ECD spectra of **3** and **4** in their neutral [20] and double-protonated forms (Boltzmann-averaged spectra based on the most populated conformers (I and II) found are given in bright blue and red colors), along with selected excitation energies and rotatory strengths indicated as ’stick’ spectra. (**b**) Selected MOs (±0.04 au) involved in the corresponding lowest-energy excitations. Numbers listed are orbital energies in eV. LC-PBE0*/SV(P)/PCM(CH${}_{3}$CN) calculations.

TDDFT calculations accurately reproduce the shifts in the energetic positions and changes in sign of the lower-energy ECD bands for the investigated compounds upon their protonation (see Figure 11 for the representative results for **3** and **4** and the Supplementary Materials for a complete set of computed data) along with the relative positions of these bands within the series of protonated species (see Figure 8). This enables a conclusive assignment of the particular bands and, consequently, rationalization of the experimentally observed trends.

Comparative inspection of the orbital energies of MOs involved in excitations underlying the low-energy ECD intensity for both neutral and protonated forms of **1**–**7** revealed that proton binding has a pronounced stabilizing impact on the low unoccupied MOs for all the double-protonated species (vs. their neutral parent molecules), and to a lesser extent, on the high-lying occupied MOs. This imbalanced stabilization leads to a reduction in the respective MO gaps, including the HOMO/LUMO gap, providing a rational explanation for the observed red-shift of the low-energy ECD bands of **1**–**7** under acidic conditions. Equally important, protonation also affects the distribution of π-electron density in these frontier MOs. For example, the HOMO isosurfaces for the neutral forms of **3**-II and **4**-I (Figure 11b) are almost evenly distributed throughout the whole molecule, but upon protonation they become predominantly localized on either the central biphenyl or the terminal aromatic rings for **3** and **4**, respectively. Such polarization of the electron density within the molecular structure may pave the way for the induction of low-energy charge-transfer (CT) excitations.

Analysis of the results computed for (+)-**4** (Figure 11), representative of the *closed* compounds, revealed that while for both neutral and protonated forms of this system the intense positive low-energy ECD band originates from excitation no. 1, for the latter species this excitation indeed demonstrates a CT-like π-to-π* character (involving HOMO-to-LUMO and HOMO-1-to-LUMO+1 transitions) instead of purely (HOMO-to-LUMO) π-to-π*, as it is under pH-neutral conditions. It also appears that the more enhanced the CT character of the electronic transitions contributing to this lowest-energy excitation, the more significant changes in the first low-energy ECD band occurs upon protonation of the *closed* systems. Specifically, the most notable red-shift among these compounds is observed for **7**, which can be attributed to the pronounced CT component from the dibenzofuran moiety to the terminal aromatic rings of the structure within the HOMO-1-to-LUMO transition (see Figure S2.8 in the Supplementary Materials). Regarding (+)-**3**, shown here as a representative of the *open* systems, the simulated ECD spectra in Figure 11a represent Boltzmann-averaged envelopes for two dominant conformers I and II (compare with Figure 6 and see Figures S2.1 and S2.3 in the Supplementary Materials). In comparison to the neutral form, the individual spectral envelopes for the protonated species appear to be less affected by the structural flexibility/adopted conformation, and the observed increase in the negative ECD intensity in the low-energy range can be assigned to excitation no. 1 for both conformers. In contrast to the corresponding excitations computed for the neutral structures, this excitation is slightly more energetically separated from the neighbouring excitations nos. 2 and 3, which exhibit rotatory strengths of opposite sign and overall lower values. Consequently, its intensity is not so effectively suppressed for the protonated species vs. non-protonated ones, thus producing a significantly negative band. The aforementioned excitation no. 1 demonstrates a clear CT character. In the case of conformer I, it is assigned to HOMO-to-LUMO and HOMO-1-to-LUMO+1 transitions between the central part of the molecule and its pyridine rings, while for conformer II, it corresponds to HOMO-1-to-LUMO and HOMO-to-LUMO+1 transitions between the central biphenyl and pyridine rings; for the corresponding MOs isosurfaces, see Figure 11b. For a comparison, the lowest-energy excitation affording the negative rotatory strength for the neutral system represents predominantly HOMO-1-to-LUMO/HOMO-to-LUMO+1 (π-to-π*) and HOMO-to-LUMO+1/HOMO-2-to-LUMO (CT-like) transitions for conformers I/II, thus showing the mixed π-to-π* and CT nature. The negative intensity observed in the *open* systems can be thus traced back to several factors, including (i) a reduced cancellation due to the more uniform spectra of different protonated conformers (we note in passing that for compound **5**, the conformers varying in helical folding and/or positions of introduced protons demonstrate quite distinct spectral envelopes but in each case showing negative intensity of the lowest-energy band, preserved upon Boltzmann-averaging), (ii) a shift of the low-energy excitations exhibiting negative rotatory strength value preventing suppression of their intensity, and (iii) a visible increase of the CT contributions in the electronic transitions for **3** and **5**, or less extended π-electron delocalization (reduced to the diphenyl moiety) for **1**.

In line with the experimental data and reflecting the changes observed in the ECD intensities, the calculated OR values for **1**–**7** are also significantly affected by the proton binding, with its impact visibly depending on the molecular structure of the compound (see Table S2.3 in the Supplementary Materials). Specifically, for the *closed* systems (in their (+)-stereoisomers), the computed ORs remain strongly positive and their values increase for the protonated species compared to those of the neutral structures. This overall agrees well with the experimental results, apart from compound **6**, for which the opposite trend was observed in the measurements. A sign inversion of ORs seen upon protonation of the *open* systems, from positive to negative values for (+)-enantiomers, can be attributed, based on the TDDFT calculations, to the changes in the conformational preferences for the protonated vs. non-protonated forms, namely increased population of conformer II with perpendicular-like arrangement of the aromatic rings (compare with Figure 6 and see Figures S2.1 and S2.3 in the Supplementary Materials), in the structure of which a better stabilization of the introduced protons can be achieved. Such conformers demonstrate negative OR values that are additionally visibly enhanced as compared to the neutral state, enabling, on average, a cancellation of positive contributions from another significantly populated form, conformer I demonstrating helicene-like folding (compare with Figure 6 and see Figures S2.1 and S2.3 in the Supplementary Materials), which on the other hand demonstrates diminished OR values upon protonation. Accordingly, the computational results successfully reproduce the sign change in OR upon proton binding for (+)-**5**, whereas for (+)-**1** and (+)-**3**, the Boltzmann-averaged OR values are (only) significantly reduced (become less positive). The latter discrepancy with the experimental data may be due to errors in both energetic assessment of examined conformers (underestimated/overestimated contribution of conformers II/I in the overall population) and determination of OR magnitude, originated from the adopted computational protocol and model.

Apart from the aforementioned red-shift of the unpolarized and circularly polarized emission spectra for both the *open* and *closed* **1**–**7** compounds upon their protonation, interestingly, for the former (*open*) derivatives, the CPL recorded under acidic conditions shows the same sign inversion of the signal as observed for their ORD: negative/positive vs. positive/negative for (+)-enantiomer/(−)-enantiomer of the protonated vs. of the neutral species (Figure 7). For both the neutral and protonated forms of the *closed* structures, on the contrary, the same sign of the CPL signal is detected: positive and negative for (+)- and (−)-enantiomer, respectively. The variations in dissymmetry factor values upon proton binding differ across the series (Table 2). For the neutral *open* molecules, only compound **3** exhibits the CPL signal. The $g_{lum}$ value for (+)-**3** is nearly equal in magnitude to that for (+)-**3**,2H${}^{+}$, but, as aforementioned, has the opposite sign. Acidification of the medium switches on the CPL response for **1** and **2**, which then show comparable (ca. 10${}^{-3}$) dissymmetry factor magnitudes, whereas for the *closed* systems, the $g_{lum}$ values for the protonated form remain either almost the same (**4** and **7**) or decrease significantly (**6**) compared to those for the corresponding neutral species. Regarding the fluorescence quantum yield values for the examined systems, they are often below 1%, and the effect of protonation is not measurable, except for derivatives **3** and **4**, possessing two benzoquinolines in their skeleton. Interestingly, in these two cases, the quantum yield visibly increases upon proton binding with a particularly significant rise (by one order of magnitude) observed for the *closed* system **4** (see Table 2).

Calculated emission properties of **1**–**7** in their protonated forms (see Table S2.1 in the Supplementary Materials) overall deviate from experimental data, even more significantly as was noticed for their neutral parent precursors [20]. This is not unexpected as the charged protonated systems are very challenging for the calculations due to solvent and counter-ion effects [17,19,33]. While the aforementioned red-shift in the emission spectra of **1**–**7** upon proton binding is not always correctly reproduced (even qualitatively) and the computed $g_{lum}$ values are significantly overestimated compared to the measured ones, importantly, the calculations support the experimentally observed protonation-triggered change in the sign of the CPL signal for the *open* compounds and its preservation for the *closed* systems.

As the luminescence dissymmetry factor is computationally defined as $g_{lum}=4R/D$, its sign is determined by the sign of the rotatory strength of the $S_{1}\to S_{0}$ emission transition (as the corresponding dipole strength is, by definition, positive), which, according to the expression R=|d|·|m|·cos(φ) (vide supra), can be in turn traced back to the angle between electric **d** and magnetic **m** transition dipole moment vectors with an acute/obtuse angle translating into positive/negative *R*. The detailed analysis of the computed values (see Table S2.2 in the Supplementary Materials) revealed that for the rigid *closed* systems no drastic change in the φ angle is observed for the protonated vs. neutral species (it remains acute), preserving the positive sign of *R* and thus of $g_{lum}$ for (+)-enantiomers. This is in line with rather similar changes in the character of $S_{1}\to S_{0}$ emission transition observed for such systems, from ππ* delocalized across almost the whole molecular structure and accompanied by nπ* component (*n* representing a lone pair of the nitrogen atoms) to CT-like ππ*; see Figures S2.7 and S2.9 in the Supplementary Materials. As shown in ref. [20] for the neutral species and revealed here for the protonated ones, for the *open* systems, due to their pronounced flexibility, a co-existence of their various conformers may also occur in the $S_{1}$ excited state. Interestingly, although the same alternation of the $S_{1}$ excited state nature, as it was demonstrated for the *closed* molecules, is also present for the *open* structures upon proton binding, in the case of their conformer I (see Figure S2.10 in the Supplementary Materials), a change in the orientation between electric and magnetic transition dipole moment vectors (from the acute to obtuse angle between them for (+)-stereoisomer) occurs for its protonated compared to non-protonated form, resulting in the negative sign of its corresponding *R* and consequently negative sign of $g_{lum}$; see Table S2.2 in the Supplementary Materials. Since for conformer II, representing another significantly populated structure, the negative sign of $g_{lum}$ remains preserved, on average, the negative $g_{lum}$ values for the *open* systems are computationally obtained. We note in passing that a structural comparison of $S_{1}$ excited-state structures for the corresponding neutral and protonated species demonstrates that, in the case of benzoquinoline- and phenanthroline-based systems **3** and **5**, the alternation of the **d** and **m** angle for conformer I correlates with the increase in the biphenyl angle (see Figure S2.11 in the Supplementary Materials). The effect of such dihedral angle modifications on chiroptical properties was also found to be important in other atropisomeric systems [34] and can also be responsible for the CPL features of compounds **1**–**7** in their neutral form, in particular decreased (or even zero) CPL activity observed experimentally for the *open* systems [20].

### 2.4. Towards pH Chirosensors in Water

Thanks to their flexibility, as shown above, the *open* compounds exhibit dramatic chiroptical changes upon protonation, such as the OR and CPL sign inversion and the appearance of the intense negative ECD band for the (+)-enantiomer at wavelengths at which the corresponding neutral species are UV-vis inactive. Thus, we sought to use enantiopure **1-PEG** and **3-PEG**, soluble versions of **1** and **3**, as pH chirosensors in water.

**Figure 12:**
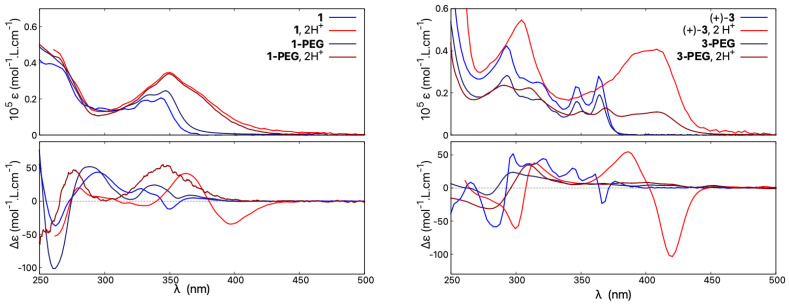
Comparison of absorption (**top**) and ECD spectra (**bottom**) of **1** and **1-PEG** (**left**) and of **3** and **3-PEG** (**right**). Measurements for **1** and **3** were performed in CH${}_{3}$CN and CH${}_{3}$CN/TFA, and for **1-PEG** and **3-PEG** in H${}_{2}$O and H${}_{2}$O/HCl. For clarity, only spectra for the (+)-enantiomers are presented. For detailed measurement conditions, see Section 4.

Accordingly, the UV-vis absorption and ECD spectra of **1-PEG** were recorded in water and compared to that of **1** measured at the same concentration in acetonitrile; see Figure 12, left. As can be seen, similar spectral envelopes are obtained for the absorption of both compounds in neutral or acidic medium, with a clear and similar protonation fingerprint visible. The ECD responses for **1** and **1-PEG** in their neutral forms also resemble each other, although more pronounced energy shifts and intensity changes of particular bands are visible compared to UV-vis. However, the ECD spectrum of **1-PEG** in H${}_{2}$O/HCl (upon protonation) is barely red-shifted between 325 and 400 nm and does not show any intense low-energy negative band observed for protonated species of all non-pegylated *open* compounds. Instead, the resulting ECD signal features a weak broad positive band centred at 350 nm.

The compound **3-PEG** shows a similar red-shift to **3** of its absorption upon acidification but with a lower molar extinction coefficient magnitude, in line with its generally less intense UV-vis signal in neutral medium (vs. **3** ), see Figure 12, right. Regarding ECD, the hydrophilic **3-PEG** presents a very poorly structured ECD spectrum in water under both neutral and acidic conditions. Similar to **1-PEG**, upon protonation, the ECD signal at low energies features a broad positive band for the (+)-enantiomer instead of an intense negative one.

**Figure 13:**
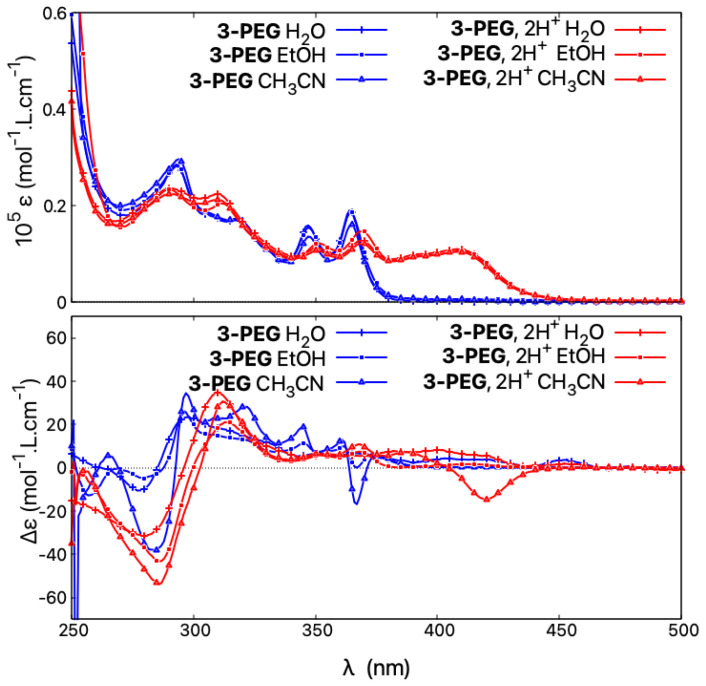
Comparison of absorption (top) and ECD spectra (bottom) of (+)-**3-PEG** in H${}_{2}$O, H${}_{2}$O/HCl, EtOH, EtOH/TFA, and CH${}_{3}$CN, CH${}_{3}$CN/TFA.

To further investigate why **1-PEG** and **3-PEG** do not follow the acid/base-triggered switching trend established for the other (non-pegylated) *open* systems **1**, **3**, and **5**, and do not demonstrate an intense negative band at low energy for their protonated (+)-enantiomers, the spectra of **3-PEG** were measured in various solvents under neutral and acidic conditions. As seen in Figure 13, the absorption properties show no solvatochromism and, regardless of the solvent employed in the study, the resulting spectra are superimposable in both neutral and acidic medium, the latter showing the typical protonation fingerprint. However, the solvent used visibly affects the ECD responses. Under neutral conditions, different spectra are obtained in water, ethanol, or acetonitrile. For example, the ECD signal at 367 nm demonstrates a Δϵ value of +7 mol${}^{-1}\cdot$L·cm${}^{-1}$ in water, 0 mol${}^{-1}\cdot$L·cm${}^{-1}$ in ethanol, and −17 mol${}^{-1}\cdot$L·cm${}^{-1}$ in acetonitrile, the latter being similar to what was found for **3** in the same solvent. In acidic medium, the same behaviour can be observed, as, for example, the ECD intensity at 422 nm changes from weakly positive in water/HCl to four times more intense and with the opposite sign in acetonitrile/TFA. The ECD spectral fingerprint of *open* **3** in its protonated state is thus restored if the spectra of **3-PEG** are recorded in acetonitrile.

Accordingly, it appears that the ECD spectra of the pegylated molecules are strongly dependent on the dielectric constant of the solvent. In acidic polar and protic solvents with a high dielectric constant, such as water, the recorded ECD responses resemble those of the *closed* structures, with the same intensity of the lower-energy band but red-shifted and broadened compared to that obtained in neutral medium. When the dielectric constant of the medium is low, as in acetonitrile or acetonitrile/TFA, the behaviour is the same as for *open* structures and an intense red-shifted negative band appears. We speculate that in acidic polar and protic solvents the two PEG chains are strongly interacting with each other and with surrounding solvent molecules, making the structures less flexible than their methylated counterparts and consequently promoting different conformational preferences, which, as shown by the calculations presented here for the *open* systems, may strongly affect chiroptical properties. However, further computational studies on such pegylated molecules, including molecular dynamic simulations to ensure a good sampling of large conformational space of these systems, are needed to fully rationalize the observed experimental trends.

## 3. Conclusions

In summary, we reported a photophysical and chiroptical study on a series of helicoidal systems including flexible (non-constrained) *open* derivatives **1**, **3**, and **5** and more rigid (constrained) *closed* compounds **2**, **4**, **6**, and **7**. The quinoline, benzoquinoline, or phenanthroline moieties (with nitrogen atoms available for protonation) included in their skeletons enabled us to investigate and compare the acid/base reactivity (basicity) of these systems and their use as potential multifunctional protonation-triggered switches. We showed that, thanks to their structural flexibility and consequently feasibility of providing better stabilization of the introduced protons, as established by the DFT calculations for neutral, mono-, and double-protonated species within this series, the *open* derivatives have higher pKa values (especially for the mono-protonation process) than the corresponding *closed* molecules. More importantly, both types of compounds demonstrate clear protonation fingerprints in their absorption, unpolarized emission, ECD, CPL, and ORD spectra, with noticeable changes in energetic position and intensity/magnitude of the signal observed for protonated vs. neutral species. Such spectral modifications are particularly pronounced for the *open* systems, for which, apart from the typical red-shift of the spectra, the appearance of an intense negative low-energy ECD band for the (+)-enantiomer along with OR and CPL sign inversion can be observed upon proton binding. Based on the results of the TDDFT calculations, these alternations can be traced back to changes in the electronic structure of the protonated vs. neutral species that are responsible for the bathochromic shift of dominant electronic transitions and for dictating their CT-type rather than π-to-π* character, and to structural flexibility that leads to different conformational preferences upon proton binding. The different central bridging ring in **7** as compared to the other *closed* compounds **2**, **4**, and **6** (furan vs. 1,3-dioxepin) visibly affects the structural and (chir)optical properties observed for this system, in both the neutral and protonated forms, which appears to stem mainly from the rigidity and fully π-conjugated electronic structure of the formed dibenzofuran moiety. Reversibility and reproducibility of the ECD modifications upon successive protonation and deprotonation processes was illustrated by an experiment involving four acidification/basification cycles that proved the acid/base-triggered chiroptical switching ability in the examined series of compounds. Finally, we developed and presented water-soluble pegylated analogues of two *open* systems, which, although showing less pronounced changes in their ECD signal for neutral vs. protonated states than was observed for their parent non-pegylated counterparts, could be considered as potential pH sensors.

## 4. Materials and Methods

### 4.1. Experimental Procedures

#### 4.1.1. General Methods

Normal atmospheric conditions were utilized in all experiments. Most reagents were used as purchased (without further purification), unless otherwise specified. In analytical thin-layer chromatography, glass plates coated with silica gel (0.25 mm 230–400 mesh) containing a fluorescent indicator were employed; silica gel (spherical neutral, particle size 63−210 μm) was also used in column chromatography. ${}^{1}$H and ${}^{13}$C NMR spectra measurements were performed at, respectively, 500.10352 MHz and 125.76408 MHz with a Bruker Avance II 500 MHz spectrometer or at, respectively, 400.140 MHz and 100.615 MHz with a Bruker Avance III 400 MHz spectrometer equipped with a Prodigy broad band probe. The corresponding chemical shifts (relative to Me${}_{4}$Si (TMS) standard) are reported in ppm. Mass spectrometry (HRMS) measurements were carried out by the Centre de Spectrométrie de Masse, University of Lyon, France. UV-vis, ECD, CPL, and ORD spectral data were obtained using in-house-built spectrometers at Institut Lumière Matière Lyon. A Horiba Jobin Yvon Fluorolog-3 fluorimeter was employed to determine absolute quantum yields utilizing an absolute quantum yield measurement system with an integrating sphere.

#### 4.1.2. Synthetic protocols

Compounds **1**–**6**, **1-PEG**, and **1-OTf** were synthesized as previously reported in ref. [20,22,24].

Preparation of 2,5,8,11,14-pentaoxahexadecan-16-yl 4-methylbenzensulfonate (**MePEG5Tos**):

NaOH pellets (284 mg, 7.35 mmol) are dissolved in water (1 mL), and commercial MeOPEG5 (1.07 g, 4.24 mmol) dissolved in THF (1 mL) is added. At 0 ${}^{\circ }$C, tosyl chloride (808 mg, 4.24 mmol) dissolved in THF (1 mL) is added dropwise. The mixture is stirred at room temperature for 24 h. Water (5 mL) is added, and the organic layer is extracted three times with Et${}_{2}$O (7 mL). The combined organic layers are washed with a saturated solution of NaCl (5 mL) and dried over Na${}_{2}$SO${}_{4}$. The solvent is removed under reduced pressure to give the product **MePEG5Tos**, which is used without further purification (1.29 g, 78%). ${}^{1}$H NMR (CDCl${}_{3}$, 500.10 MHz): δ (ppm) = 7.79 (d, *J* = 8.32 Hz, 2H), 7.34 (d, *J* = 8.33 Hz, 2H), 4.15 (t, *J* = 5.10 Hz, 2H), 3.52–3.71 (m, 18H), 3.36 (s, 3H), 2.44 (s, 3H). ${}^{13}$C NMR (CDCl${}_{3}$, 125.76 MHz): δ (ppm) = 144.78 (Cq), 133.01 (Cq), 129.82 (2 CH), 127.95 (2 CH), 71.91 (CH${}_{2}$), 70.71 (CH${}_{2}$), 70.58 (2 CH${}_{2}$), 70.54 (2 CH${}_{2}$), 70.50 (CH${}_{2}$), 70.48 (CH${}_{2}$), 69.25 (CH${}_{2}$), 68.65 (CH${}_{2}$), 58.99 (CH${}_{3}$), 21.61 (CH${}_{3}$). HRMS (ESI) [M + H]${}^{+}$: calcd. for C${}_{18}$H${}_{31}$O${}_{8}$S 407.1734; found: 407.1737.

Preparation of (−)- and (+)-2,2’-bis(2,5,8,11,14-pentaoxahexadecan-16-yloxy)-5,5’,6,6’- tetrahydro-1,1’-dibiben[c,h]acridine ((−)- and (+)-**3-PEG**):

Step 1—demethoxylation: Under argon at 0 ${}^{\circ }$C, a solution of BBr${}_{3}$ (1 M in CH${}_{2}$Cl${}_{2}$, 1.6 mL, 1.6 mmol) is added dropwise to a solution of (+)-**3** (100 mg, 0.19 mmol) in dry CH${}_{2}$Cl${}_{2}$ (2 mL). The mixture is stirred for 15 h at room temperature, then quenched at 0 ${}^{\circ }$C by dropwise addition of EtOH (1 mL). Et${}_{2}$O (5 mL) is added and the yellow precipitate is filtered off and washed with Et${}_{2}$O (5 mL) and not purified further (125 mg, 99%). ${}^{1}$H NMR (d6-DMSO, 500.10 MHz): δ (ppm) = 8.04 (s, 2H), 7.26 (d, *J* = 8.13 Hz, 2H), 7.26 (d, *J* = 8.75 Hz, 2H), 7.71 (m, 2H), 7.68 (d, *J* = 8.70 Hz, 2H), 7.27 (ddd, *J* = 8.45, 6.88, 1.08 Hz, 2H), 7.09 (d, *J* = 8.00 Hz, 2H), 6.75 (d, *J* = 8.00 Hz, 2H), 2.95–2.08 (m, 6H), 2.59 (m, 2H). HMRS (ESI) [M + H]${}^{+}$: calcd. for C${}_{42}$H${}_{28}$N${}_{2}$O${}_{2}$ 593.2224; found: 593.2202.

Step 2—alkylation: Cs${}_{2}$CO${}_{3}$ (209 mg, 0.64 mmol) is added to a solution of demethoxylated (+)-**3** (110 mg, 0.15 mmol) and **MePEG5Tos** (125 mg, 0.32 mmol) in DMF (9 mL). The solution is heated at 100 ${}^{\circ }$C for 15 h. After cooling, DMF is evaporated and replaced with CH${}_{2}$Cl${}_{2}$ (50 mL). The formed precipitate is filtered off and the filtrate is concentrated under reduced pressure to give the crude product, which is purified by chromatography over silica gel eluted with a gradient of CH${}_{2}$Cl${}_{2}$ from 100:0 to 80:20, giving (+)-**3-PEG** as a brown oil (106 mg, 65%). ${}^{1}$H NMR (CDCl${}_{3}$, 500.10 MHz): δ (ppm) = 7.82 (d, *J* = 7.68 Hz, 1H), 7.73 (d, *J* = 7.68 Hz, 1H), 7.60 (m, 2H), 7.51 (td, *J* = 7.34, 1.17 Hz, 1H), 7.45 (d, *J* = 8.84 Hz, 1H), 7.33 (td, *J* = 7.61, 1.04 Hz, 1H), 7.22 (d, *J* = 8.32 Hz, 1H), 7.02 (d, *J* = 8.06 Hz, 1H), 4.01 (m, 1H), 3.82 (m, 1H), 3.63-3.32 (m, 21H), 2.85 (m, 2H), 2.76 (td, 1H), 2.07 (m, 1H). ${}^{13}$C NMR (CDCl${}_{3}$, 125.76 MHz): δ (ppm) = 155.99 (Cq), 152.83 (Cq), 144.01 (Cq), 134.32 (Cq), 133.59 (Cq), 132.80 (Cq), 132.38 (CH), 132.37 (Cq), 131.93 (Cq), 129.78 (Cq), 127.11 (CH), 126.92 (CH), 126.64 (CH), 126.37 (CH), 125.78 (CH), 125.73 (CH), 124.70 (CH), 124.45 (Cq), 114.51 (CH), 71.93 (CH${}_{2}$), 70.56 (CH${}_{2}$), 70.53 (CH${}_{2}$), 70.49 (2 CH${}_{2}$), 70.46 (CH${}_{2}$), 70.37 (CH${}_{2}$), 69.53 (CH${}_{2}$), 69.14 (CH${}_{2}$), 59.03 (CH${}_{3}$), 29.37 (CH${}_{2}$), 29.21 (CH${}_{2}$). HRMS (ESI) [M + H]${}^{+}$: calcd. for C${}_{64}$H${}_{72}$N${}_{2}$O${}_{12}$ 1061.5158; found: 1061.5147.

The same procedure as described above for (+)-**3-PEG** is used for the synthesis of the (−)-enantiomer. In a typical scale experiment, (−)-**3-PEG** (113 mg, 67%) is obtained starting from 120 mg of (−)-**3**.

Preparation of (+)-**7** and (−)-**7**:

Synthesis of (±)-**7**: A mixture of **1-OTf** [24] (100 mg, 0.132 mmol) and K${}_{3}$PO${}_{4}$ (560 mg, 2.64 mmol) are stirred for 5 days at 140 ${}^{\circ }$C in a Schlenk tube. The mixture is cooled down, and CH${}_{2}$Cl${}_{2}$ and water are added. The organic layer is separated, and the aqueous layer is extracted with CH${}_{2}$Cl${}_{2}$ twice. The combined organic layers are dried over Na${}_{2}$SO${}_{4}$ and evaporated. Purification of the crude residue by silica gel column chromatography with CH${}_{2}$Cl${}_{2}$ as the eluent give (±)-**7** as a pale yellow solid (20 mg, 0.042 mmol, 32%). ${}^{1}$H NMR (CDCl${}_{3}$, 400.10 MHz): δ (ppm) = 7.63 (d, *J* = 8.2 Hz, 2H), 7.44 (d, *J* = 8.2 Hz, 2H), 7.42 (d, *J* = 8.5 Hz, 2H), 7.31 (ddd, *J* = 8.0, 6.8, 1.5 Hz, 2H), 7.19 (ddd, *J* = 8.0, 6.8, 1.5 Hz, 2H), 7.13 (s, 2H), 7.10 (dd, *J* = 8.0, 1.5 Hz, 2H), 3.37 (dddd, *J* = 14.9, 13.6, 4.8, 1.6 Hz, 1H), 3.11 (ddd, *J* = 15.5, 4.8, 1.8 Hz, 1H), 3.03–2.81 (m, 2H). ${}^{13}$C NMR (CDCl${}_{3}$, 125.76 MHz): δ (ppm) = 157.3 (Cq), 155.0 (Cq), 145.8 (Cq), 135.8 (Cq), 132.4 (Cq), 131.3 (CH), 128.4 (CH), 126.8 (CH), 126.4 (Cq), 125.2 (CH), 121.9 (CH), 112.3 (CH), 29.78 (CH${}_{2}$), 29.19 (CH${}_{2}$). HRMS (ESI) [M + H]${}^{+}$: calcd. for C${}_{34}$H${}_{23}$N${}_{2}$O 475.1803; found: 475.1805.

Resolution of (±)-**7**: 250 μL of a solution of (±)-**7** (28 mg dissolved in 2.5 mL of CH${}_{2}$Cl${}_{2}$) are injected 8 times every 8.1 minutes on a Chiralpak IE (250×10 mm) column. The two enantiomers are eluted with the following mobile phase: hexane/ethanol (with 0.1% trimethylamine)/dichloromethane (60/20/20) at a flow-rate of 5 mL/min. The detection is performed at 290 nm. The first collected fraction gives 13 mg of (+)-**7** with *ee* > 99.5%, and the second collected fraction gives 12 mg of (−)-**7** with *ee* > 99.5%. The corresponding chromatograms are presented in Figures S1.8–S1.9 in the Supplementary Materials.

#### 4.1.3. Photophysical and Chiroptical Spectra Measurements

The following specifications were used for all the spectra recorded in this study.

- Absorption and ECD were measured in acetonitrile (CH${}_{3}$CN) solutions at a concentration of approximately $5\times 10^{-5}$ mol/L. The unit of ϵ and Δϵ used for the plotting is mol${}^{-1}\cdot$L·cm${}^{-1}$.
- Fluorescence and CPL were recorded in dichloromethane (CH${}_{2}$Cl${}_{2}$) solutions at a concentration of approximately 10${}^{-3}$ mol/L.
- ORD were measured in CH${}_{2}$Cl${}_{2}$ solutions at a concentration between $5\times 10^{-3}$ and $10\times 10^{-3}$ mol/L. The unit of molar optical rotation [Φ] used for the plotting is 10${}^{-1}$ deg·mol${}^{-1}\cdot$cm${}^{2}$.
- Acidification was performed by the addition of 1 mL (13 mmol) of trifluoroacetic acid into 2 mL of the neutral solution of the examined compounds. The dilution of the sample was taken into account in the ϵ and Δϵ values of the corresponding spectra.

ECD, CPL, and ORD specta for both enantiomers of the examined compounds, either under neutral or acidic conditions, are presented in Figures S1.1–S1.5 and S1.10–S1.16 in the Supplementary Materials.

#### 4.1.4. X-ray Crystal Structure of **7**

Experimental: Single light yellow plate-shaped crystals of (±)-**7** were obtained by a slow evaporation from CH${}_{2}$Cl${}_{2}$. A suitable crystal of 0.61 × 0.20 × 0.06 mm${}^{3}$ was selected and mounted on a nylon loop in perfluoroether oil on an Xcalibur, Atlas, Gemini ultra diffractometer. The crystal was kept at a steady T = 150.04(13) K during data collection. The structure was solved with the ShelXT structure solution program [35] using the Intrinsic Phasing solution method with the Olex2 graphical interface [36]. The model was refined with version 2018/3 of ShelXL using Least Squares minimisation.

Crystal data: C${}_{34}$H${}_{22}$N${}_{2}$O, Mr= 474.53, orthorhombic, Pbca (No. 61), a = 9.3896(12) Å, b = 15.389(2) Å, c = 31.931(3) Å, α = β = γ = 90°, V = 4614.0(10) Å${}^{3}$, T = 150.04(13) K, Z = 8, Z’ = 1, m(MoKa) = 0.083, 35,035 reflections measured, 5835 unique (Rint = 0.0927), which were used in all calculations. The final wR2 was 0.1857 (all data) and R1 was 0.0676 (I > 2(I)). The XRD data were deposited in the Cambridge Structural Database under the deposition number CCDC 2291629 and can be requested free of charge via www.ccdc.cam.ac.uk, (accessed on 3 October 2019).

### 4.2. Quantum-Chemical Calculations

Calculations were performed for mono- and double-protonated forms of compounds **1**–**6**, and for neutral, mono-, and double-protonated species of the system **7**, all in their (+)-stereoisomeric structure. All computations employed DFT and TDDFT approaches, following the protocol established and successfully used in our studies on compounds **1**–**6** in their neutral form [20].

DFT geometry optimizations were performed with the BLYP density functional [37,38] and the triple-ζ valence polarization basis set, TZVP [39], using the conductor-like screening model (COSMO) [40,41] to simulate solvent (acetonitrile, CH${}_{3}$CN, ε = 35.688) effects. TDDFT calculations of UV-vis and ECD spectra employed optimally tuned long-range corrected hybrid functional based on the PBE density functional [42], LC-PBE0* with the range-separation parameter γ* set to 0.14 $a_{0}^{-1}$ [43,44,45], the split-valence basis set with one set of polarization functions for non-hydrogen atoms, SV(P) [39,46,47], and the polarizable continuum model (PCM) [48,49] for acetonitrile. The corresponding OR parameters were computed at the sodium *D*-line wavelength, λ = 589.3 nm, with LC-PBE0*/SV(P)/PCM(dichloromethane, CH${}_{2}$Cl${}_{2}$, ε = 8.93), preceded by the geometry re-optimizations employing BLYP/TZVP/COSMO(CH${}_{2}$Cl${}_{2}$). Electronic emission spectra via TDDFT $S_{1}$ excited-state geometry optimizations were obtained at the LC-PBE0*/SV(P)/ PCM(CH${}_{2}$Cl${}_{2}$) level of theory. For a full description of the computational details used in these studies along with the corresponding references [50,51,52,53,54], see the Supplementary Materials.

## Figures and Tables

**Table 1:** Protonation equilibrium constants determined for **1**–**7** based on the ${}^{1}$H NMR results obtained during titration with TFA (400 MHz, 298 K, CDCl${}_{3}$) using BindFit (http://supramolecular.org, accessed on 1 June 2023) [28,29]. pKa${}_{1}$ is defined as −log$\frac{1}{K_{01}}$ and pKa${}_{2}$ as −log$\frac{1}{K_{12}}$; see Figure 3.

Compound	First Protonation		Second Protonation	
	**K** ${}_{01}$ **(M** ${}^{-1}$ **)**	**pKa** ${}_{1}$	**K** ${}_{12}$ **(M** ${}^{-1}$ **)**	**pKa** ${}_{2}$
**1**	455 ± 9%	2.6	7.7 ± 7%	0.9
**2**	36.5 ± 10%	1.6	4.5 ± 2%	0.7
**3**	355.0± 10%	2.5	22.7 ± 6%	1.3
**4**	52.2 ± 20%	1.7	0.78 ± 7%	−0.1
**5**	53,047.4 ± 10%	4.7	0.06 ± 7%	−1.2
**6**	91.4 ± 4%	1.9	14.4 ± 6%	0.2
**7**	34.9 ± 7%	1.5	65.2 ± 16%	1.8

**Table 2:** Fluorescence quantum yields (QY) and dissymmetry factors ($g_{lum}$) measured for **1**–**7** and **1**,2H${}^{+}$–**7**,2H${}^{+}$ in, respectively, CH${}_{2}$Cl${}_{2}$ and CH${}_{2}$Cl${}_{2}$/TFA (2/3 *v*/*v*) solutions.

Compound	QY (%)	$g_{\mathrm{lum}}$	$\lambda_{\mathrm{lum}}^{\max}$
(+)-**1**	n. m.	0	437
(+)-**1**,2H${}^{+}$	1	$-1\times 10^{-3}$	545
(+)-**2**	2	0	463
(+)-**2**,2H${}^{+}$	2	$+2.5\times 10^{-3}$	550
(+)-**3**	<1	$+2.5\times 10^{-3}$	437
(+)-**3**,2H${}^{+}$	3.5	$-1.6\times 10^{-3}$	480
(+)-**4**	1.4	$+2\times 10^{-3}$	420
(+)-**4**,2H${}^{+}$	19	$+2\times 10^{-3}$	480
(+)-**5**	<1	0	592
(+)-**5**,2H${}^{+}$	<1	0	550
(+)-**6**	<1	$+8\times 10^{-3}$	437
(+)-**6**,2H${}^{+}$	<1	$+2\times 10^{-3}$	550
(+)-**7**	5	$+14\times 10^{-3}$	440
(+)-**7**,2H${}^{+}$	5	$+14\times 10^{-3}$	560

## Data Availability

The insight into detailed data might be obtained after the contact with the corresponding authors.

## Supplementary Materials

The following supporting information can be downloaded at: https://www.mdpi.com/article/10.3390/molecules28217322/s1: additional experimental data: ORD for both enantiomers of **3**–**7** (Figures S1.1–S1.5), ECD spectra recorded during switching experiment for **1** and **2** (Figures S1.6 and S1.7), chiral HPLC chromatograms for **7** (Figure S1.8), enantiomeric excess measurements for **7** (Figure S1.9), absorption, fluorescence, ECD, and CPL spectra for both enantiomers of **1**–**7** (Figures S1.10–S1.16); computational details description; additional calculated results: DFT-optimized neutral and protonated structures of **1**–**7** (Figures S2.1–S2.4), ECD spectra for neutral species of **1**–**7** (Figure S2.5), frontier MOs of neutral species of **2**, **4**, **6**, and **7** (Figure S2.6), MOs of $S_{0}$ at $S_{1}$ excited-state geometry of neutral forms of **2**, **4**, **6**, and **7** (Figure S2.7), MOs for double-protonated species of **7** (Figure S2.8), MOs of $S_{0}$ at $S_{1}$ excited-state geometry of double-protonated forms of **1**–**7** (Figure S2.9), optimized double-protonated structures of **1**–**7** in the $S_{1}$ excited state (Figure S2.10), overlays of the corresponding neutral and double-protonated structures of **1**–**7** in the ground and $S_{1}$ excited state (Figure S2.11), experimental and TDDFT-simulated UV-vis and ECD spectra for neutral and protonated species of **1**–**7** (Figures S2.12–S2.16), MOs for double-protonated species of **1**–**6** (Figures S2.17–S2.28), MOs for neutral species of **7** (Figure S2.29), optimized neutral structure of **7** in the $S_{1}$ excited state (Figure S2.30), photophysical data for **1**–**7** in the $S_{1}$ excited state (Tables S2.1 and S2.2), molar rotations for double-protonated species of **1**–**7** (Table S2.3), dominant excitations for double-protonated structures of **1**–**6** (Tables S2.4–S2.9), dominant excitations for neutral and double-protonated forms of **7** (Tables S2.10 and S2.11); Cartesian coordinates for optimized structures.

## Sample Availability

Samples of the compounds **1**–**6**, **1-PEG**, and **3-PEG** are available from the authors.

## Abbreviations

The following abbreviations are used in this manuscript:

COSMO	COnductor-like Screening MOdel
CPL	Circularly Polarized Luminescence
DFT	Density Functional Theory
ECD	Electronic Circular Dichroism
HOMO	Highest Occupied Molecular Orbital
LUMO	Lowest Unoccupied Molecular Orbital
MO	Molecular Orbital
OR	Optical Rotation
ORD	Optical Rotatory Dispersion
PCM	Polarizable Continuum Model
PEG	PolyEthylene Glycol
TDDFT	Time-Dependent DFT
TFA	Trifluoroacetic Acid
