# Supplementary material for: Acid/Base-Triggered Photophysical and Chiroptical Switching in a Series of Helicenoid Compounds
Source: Molecules. 2023 Oct 29;28(21):7322. doi: 10.3390/molecules28217322 (PMC10647711; doi:10.3390/molecules28217322)
Supplement: Supplementary file 1 [file molecules-28-07322-s001.zip › molecules-2675712-supplementary.pdf]

Supplementary Materials: Acid/Base-Triggered Photophysical and Chiroptical Switching in a Series of Helicenoid Compounds

Laure Guy [1,*], Maëlle Mosser [1], Delphine Pitrat [1], Jean-Christophe Mulatier [1], Mercedes Kukułka [2,*], Monika Srebro-Hooper [2,*], Erwann Jeanneau [3], Amina Bensalah-Ledoux [4], Bruno Baguenard [4], Stéphan Guy [4]

1. Additional experimental data

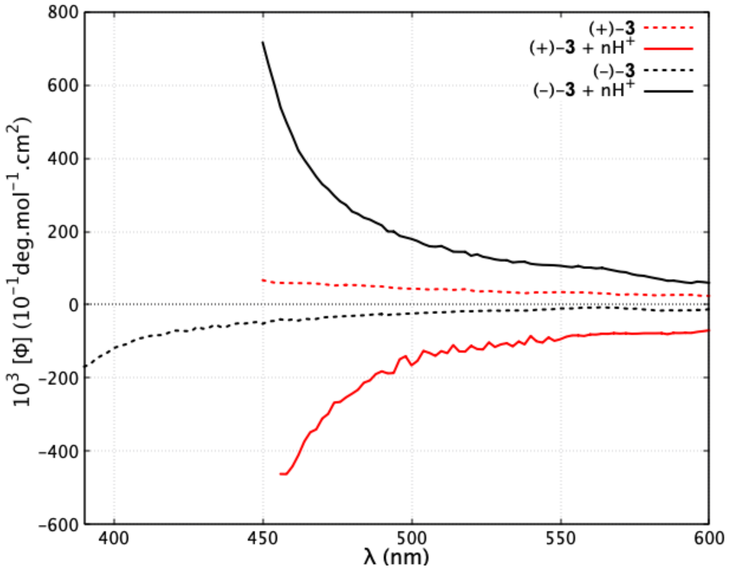

Figure S1.1. ORD spectra of (−)- and (+)-**3** in neutral and acidic medium.

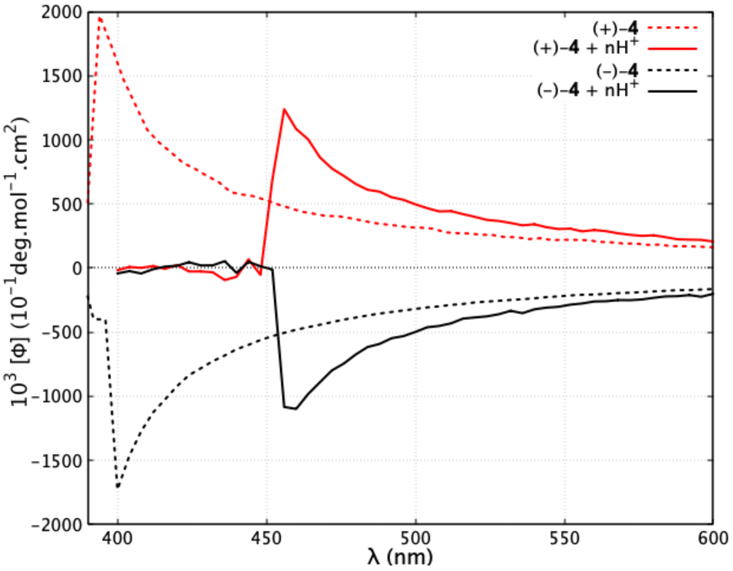

Figure S1.2. ORD spectra of (−)- and (+)-**4** in neutral and acidic medium.

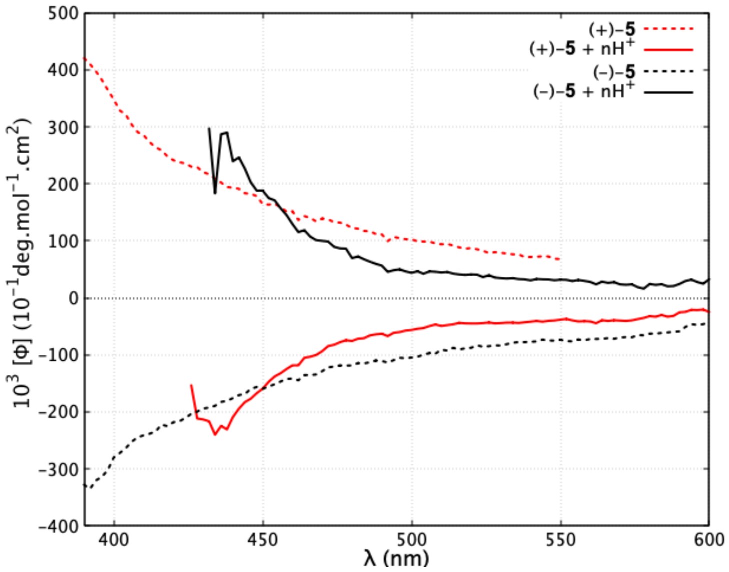

Figure S1.3. ORD spectra of (−)- and (+)-**5** in neutral and acidic medium.

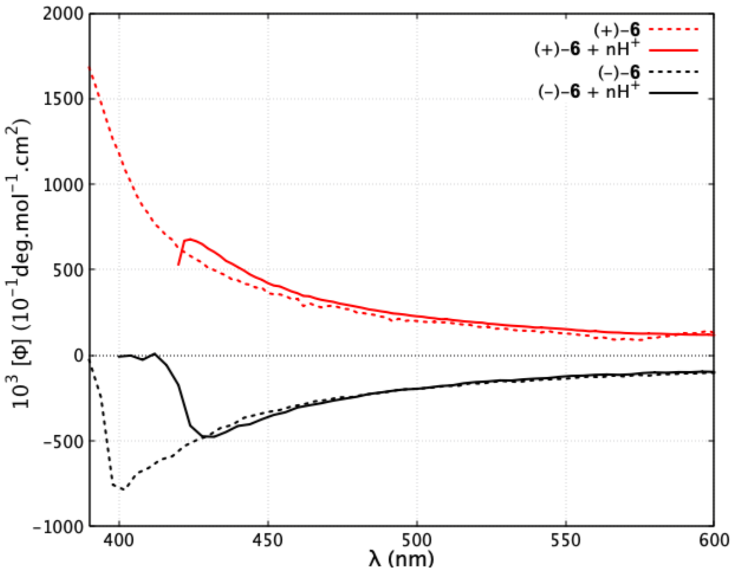

Figure S1.4. ORD spectra of (−)- and (+)-**6** in neutral and acidic medium.

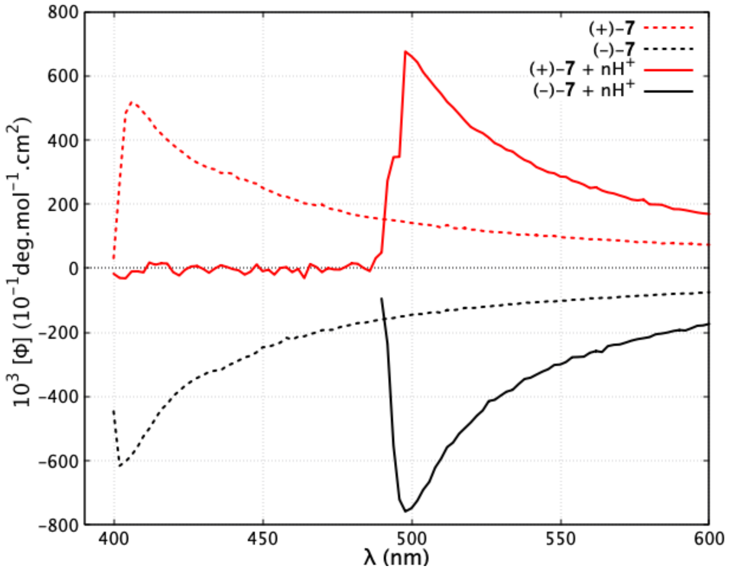

Figure S1.5. ORD spectra of (−)- and (+)-**7** in neutral and acidic medium.

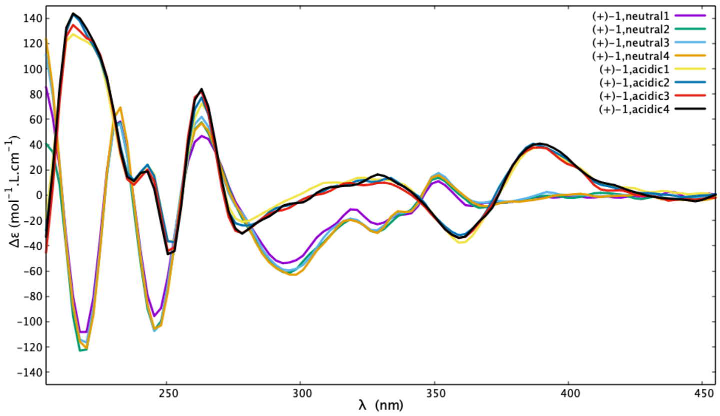

Figure S1.6. ECD spectra of (+)-**1** (c = 3.4 × 10^{-5} mol/L in CH_3CN) recorded during successive protonation/deprotonation processes. The spectra for the neutral species were measured after the deprotonation step, which was carried out by placing 3 mL of the acidic acetonitrile solution of the system in contact with a sodium hydroxide pellet. The collected solution was subsequently acidified by the addition of 1 μL of sulphuric acid. Four cycles of acidification/basification have been performed, *i.e.* eight ECD spectra have been measured.

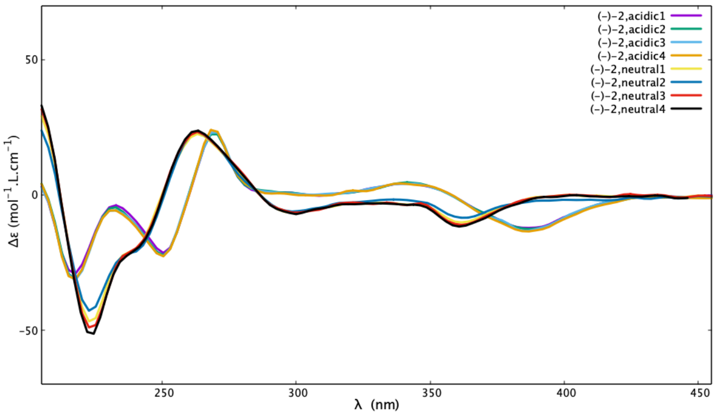

Figure S1.7. ECD spectra of (−)-**2** ($c = 4.2 \times 10^{-5}$ mol/L in CH_3CN) recorded during successive protonation/deprotonation processes. The spectra for the neutral species were measured after the deprotonation step, which was carried out by placing 3 mL of the acidic acetonitrile solution of the system in contact with a sodium hydroxide pellet. The collected solution was subsequently acidified by the addition of 1 μL of sulphuric acid. Four cycles of acidification/basification have been performed, *i.e.* eight ECD spectra have been measured.

Column	Mobile Phase	t1	k1	t2	k2	α	Rs
**Chiralpak IE**	Heptane / ethanol with triethylamine 0.1% / dichloromethane 60/20/20	4.87 (+)	0.65	6.53 (-)	1.21	1.86	7.49

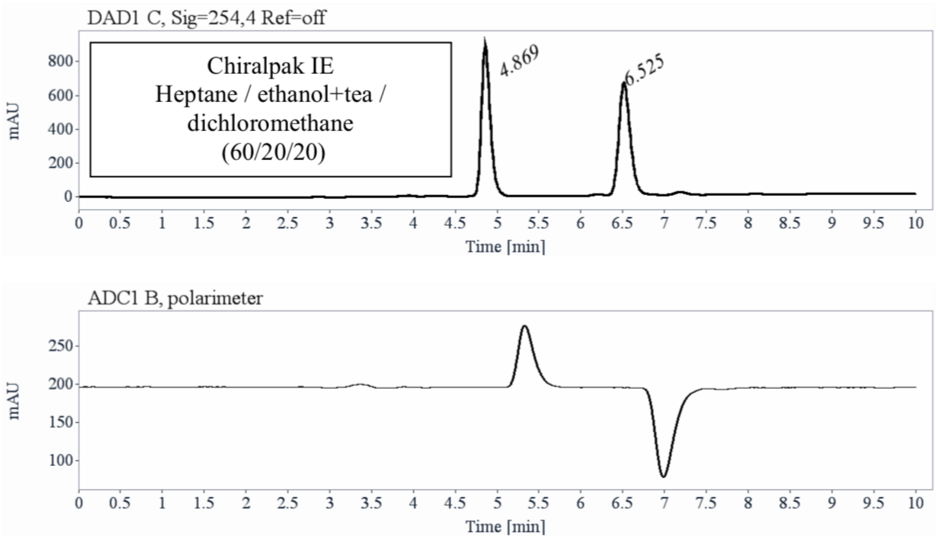

RT [min]	Area	Area%	Capacity Factor	Enantioselectivity	Resolution (USP)
4.87	6649	51.06	0.65		
6.53	6372	48.94	1.21	1.86	7.49
Sum	13022	100.00			

Figure S1.8. Analytical chiral HPLC resolution of compound (±)-**7**.

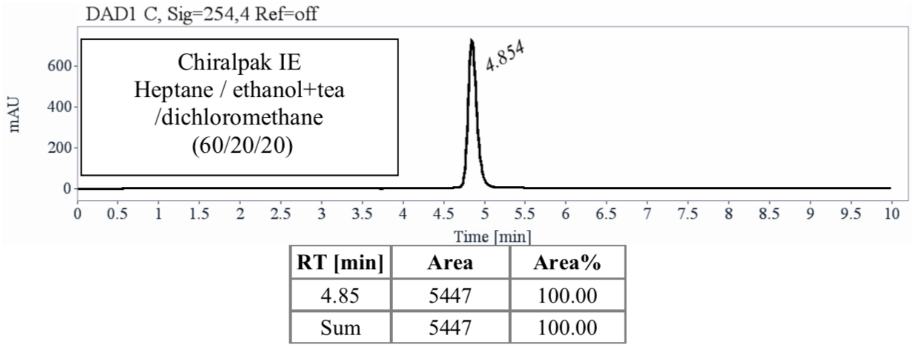

RT [min]	Area	Area%
4.85	5447	100.00
Sum	5447	100.00

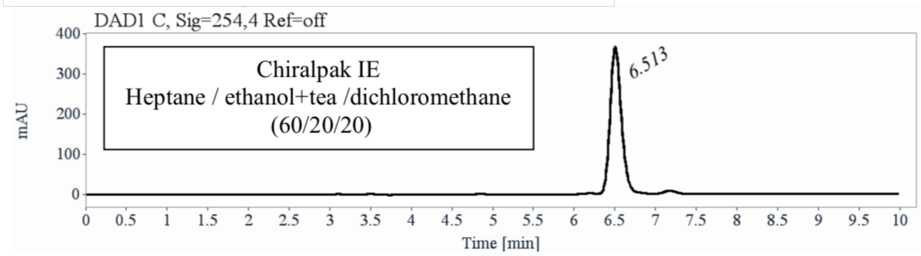

RT [min]	Area	Area%
6.51	3507	100.00
Sum	3507	100.00

Figure S1.9. Enantiomeric excess measurement of resolved (+)-**7** (top) and (−)-**7** (bottom).

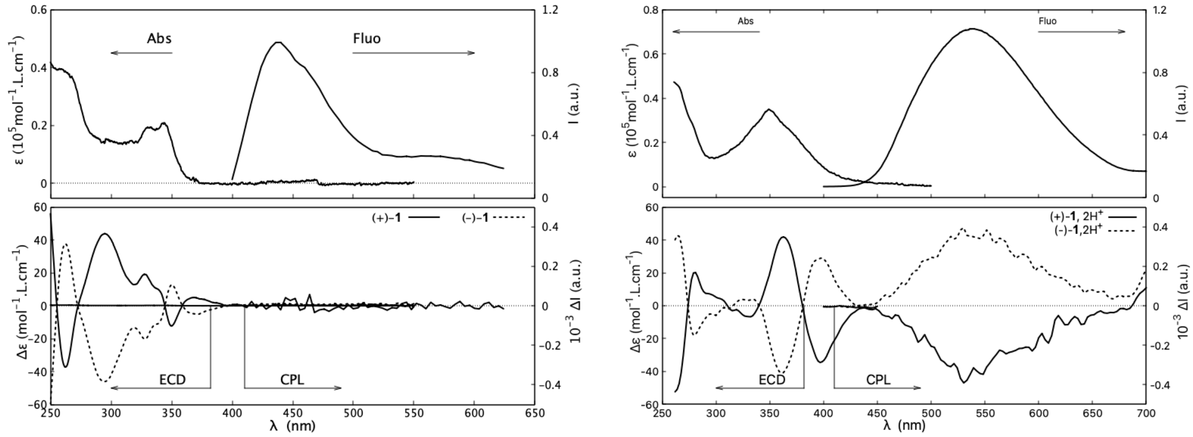

Figure S1.10. Absorption, fluorescence, ECD and CPL spectra of (˘)- and (+)-**1** in neutral and acidic medium.

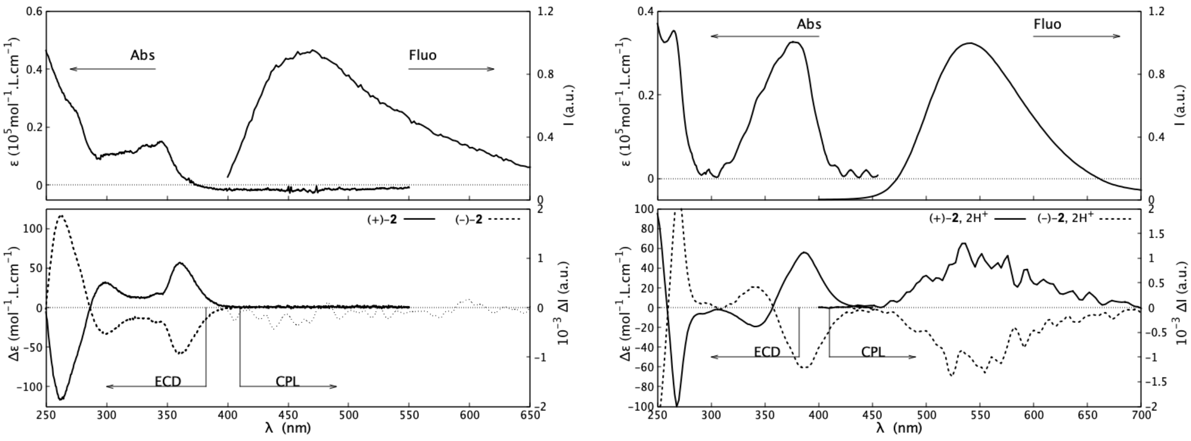

Figure S1.11. Absorption, fluorescence, ECD and CPL spectra of (−)- and (+)-**2** in neutral and acidic medium.

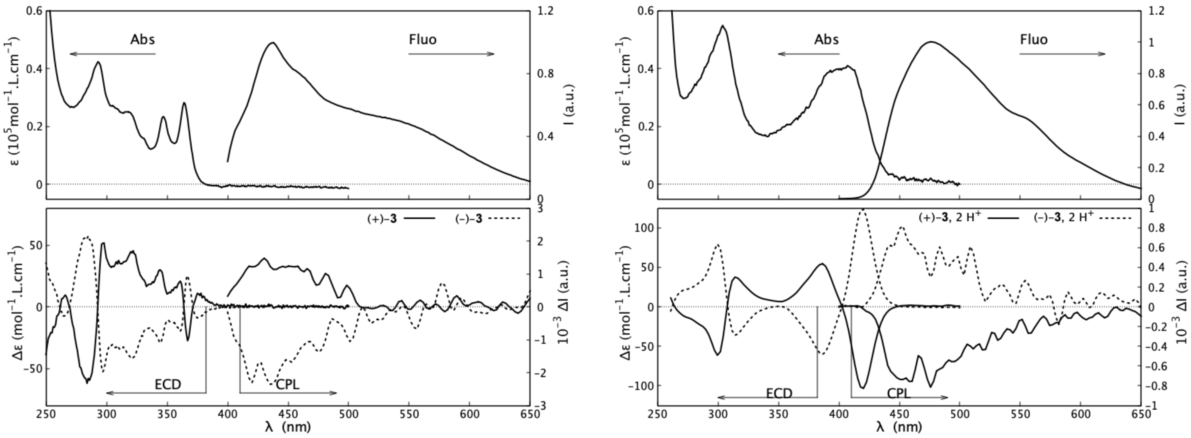

Figure S1.12. Absorption, fluorescence, ECD and CPL spectra of (−)- and (+)-**3** in neutral and acidic medium.

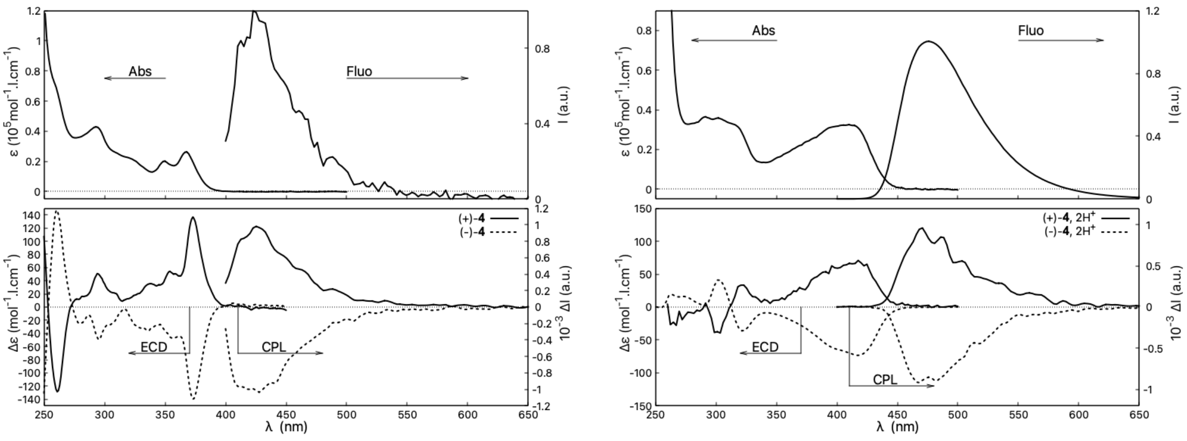

Figure S1.13. Absorption, fluorescence, ECD and CPL spectra of (−)- and (+)-**4** in neutral and acidic medium.

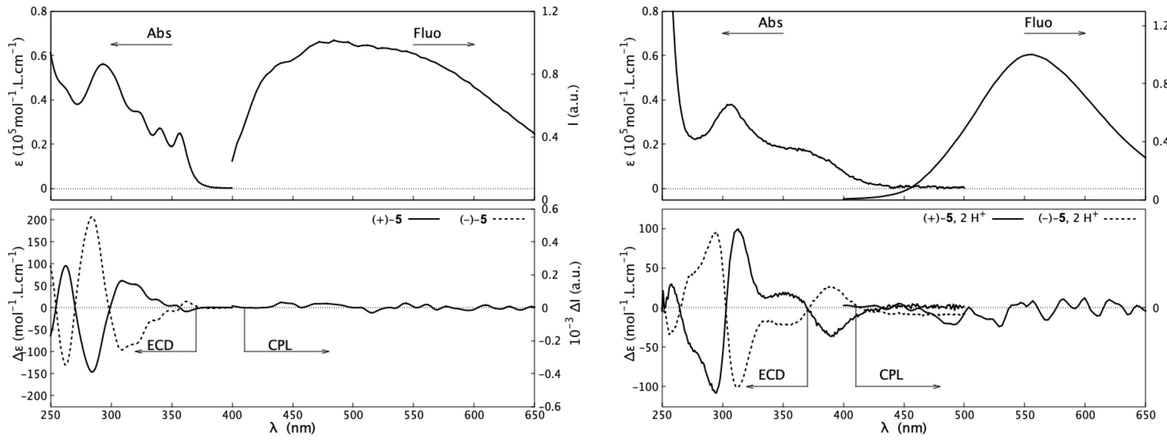

Figure S1.14. Absorption, fluorescence, ECD and CPL spectra of (−)- and (+)-**5** in neutral and acidic medium.

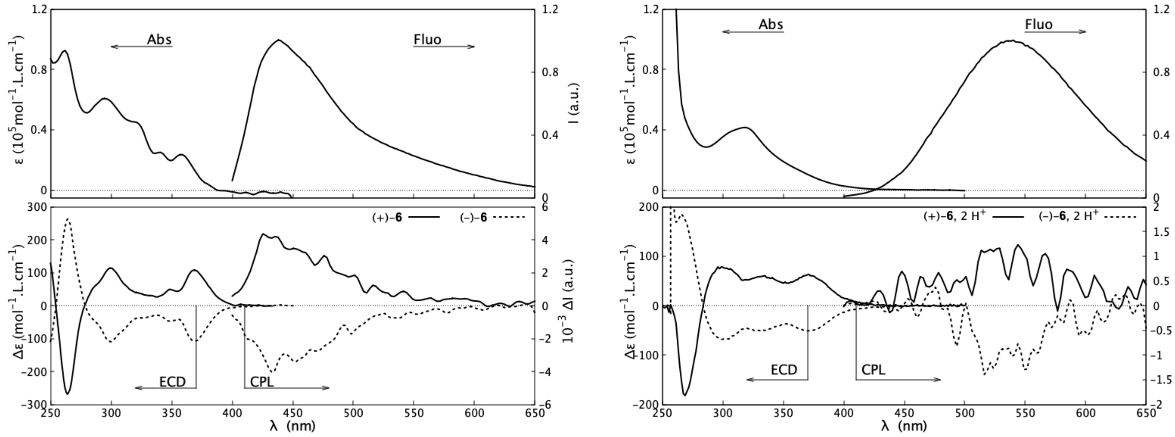

Figure S1.15. Absorption, fluorescence, ECD and CPL spectra of (−)- and (+)-**6** in neutral and acidic medium.

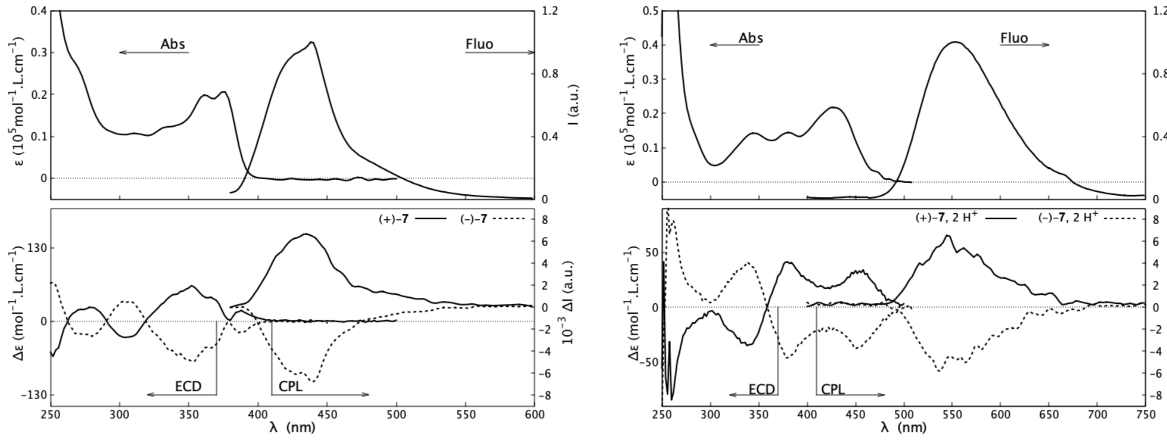

Figure S1.16. Absorption, fluorescence, ECD and CPL spectra of (−)- and (+)-**7** in neutral and acidic medium.

2. Quantum-chemical studies

2.1. Computational details

Molecular structures of mono- and double-protonated forms of the helicene-like systems **1–6** as well as of neutral and protonated species of the newly synthesized compound **7** were optimized using density functional theory (DFT) with resolution-of-identity approximation as implemented in the Turbomole package, version 7.1.[50,51] Following the recent studies on photophysical and chiroptical properties of **1–6** in their neutral state,[20,21] geometry optimizations were performed with the Becke, Lee–Yang–Parr (BLYP) exchange-correlation (XC) functional[37,38] and a triple-ζ valence polarization basis set, TZVP.[39] Solvent effects (acetonitrile, CH_3CN, ε = 35.688) were included in the calculations using the conductor-like screening model (COSMO)[40,41] with default parameters employed in the Turbomole package.

Subsequent calculations of optical rotation (OR) parameters, electronic circular dichroism (ECD) spectra, and emission properties *via* S_1 excited-state geometry optimizations were performed with time-dependent DFT (TDDFT), using the Gaussian 16 package.[52] Long-range corrected hybrid functional based on the Perdew-Burke-Ernzerhof (PBE) functional,[42] LC-PBE0*, was used with the range-separation parameter γ^* set to 0.14 a_0^{-1} that corresponds to the optimally tuned value for helicene systems.[43–45] In these calculations, a split-valence basis set with one set of polarization functions for non-hydrogen atoms, SV(P), was applied,[39,46,47] along with the polarizable continuum model (PCM)[48,49] with default parameters implemented in the Gaussian package to simulate the solvent (acetonitrile for ECD, dichloromethane, CH_2Cl_2, ε = 8.93 for OR and emission) effects. The OR calculations were proceeded by the geometry re-optimizations with BLYP/TZVP/COSMO(CH_2Cl_2) using the Turbomole package.

The TDDFT calculations covered the 120 lowest singlet excited states for each system. The corresponding spectra were simulated using the GaussView 5.0.9 program[53] as the sums of Gaussian functions centred at the vertical excitation energies and scaled using the computed rotatory strengths[54] with the implemented half-width at half-height (HWHH) factor set to 0.225 eV (which corresponds to σ = 0.2 eV). The optical rotation parameters, $[\alpha]$, were computed at the sodium D-line wavelength, λ = 589.3 nm. Related values of molar rotations were obtained from $[\alpha]$ using the molecular mass M, according to the equation:

$$[\Phi] = M \times [\alpha] \times 0.01 \tag{1}$$

All calculations were carried out for the (+)-isomers without explicitly imposing symmetry.

2.2. Additional calculated data

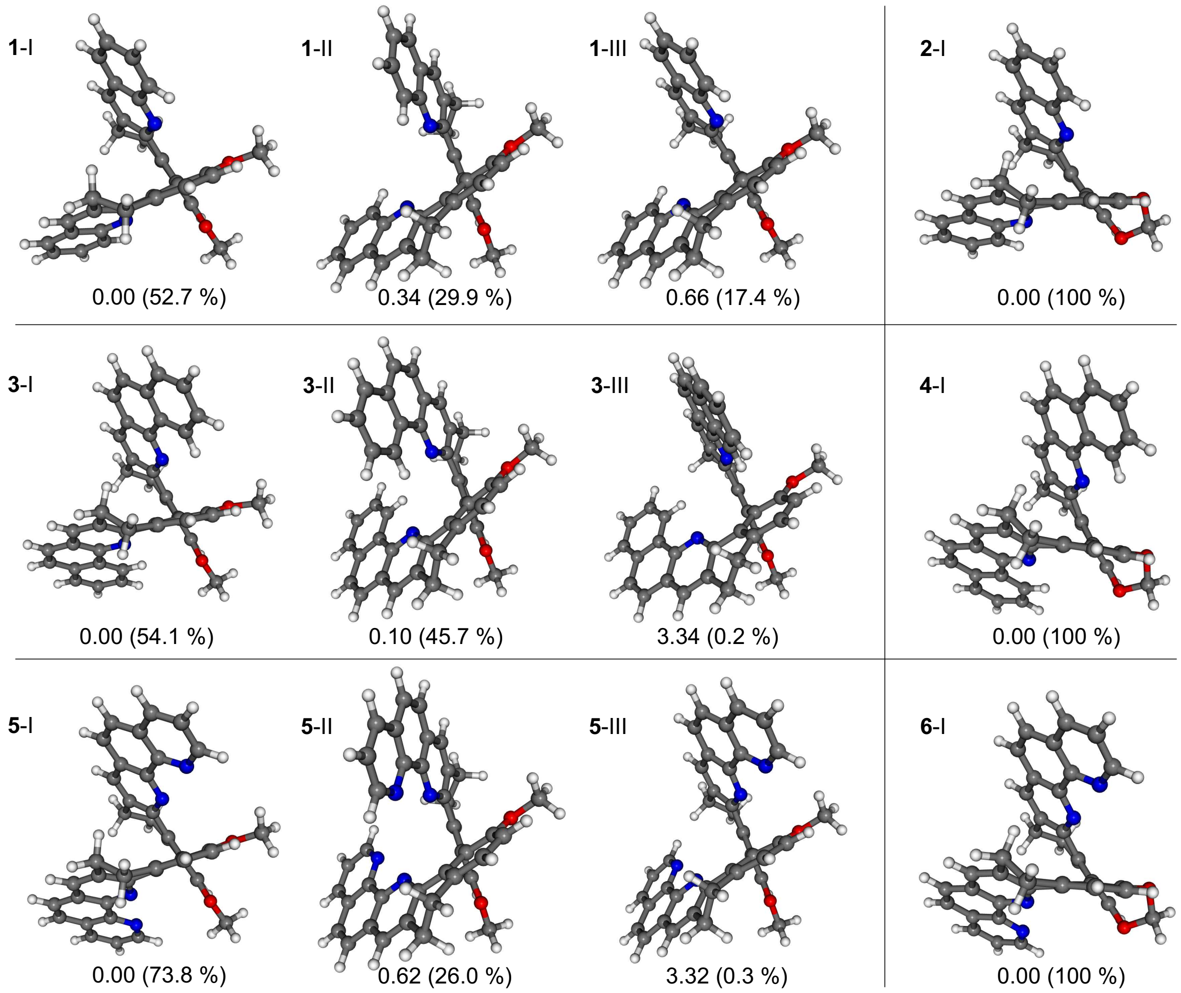

Figure S2.1. Selected low-energy DFT-optimized structures of (+)-enantiomers of **1**–**6** reported in ref. [20]. Numbers listed are relative energy values with respect to the lowest-energy conformer (in kcal mol^{-1}) and the corresponding Boltzmann populations at 298 K (provided in parentheses). BLYP/TZVP/COSMO(CH_3CN) calculations.

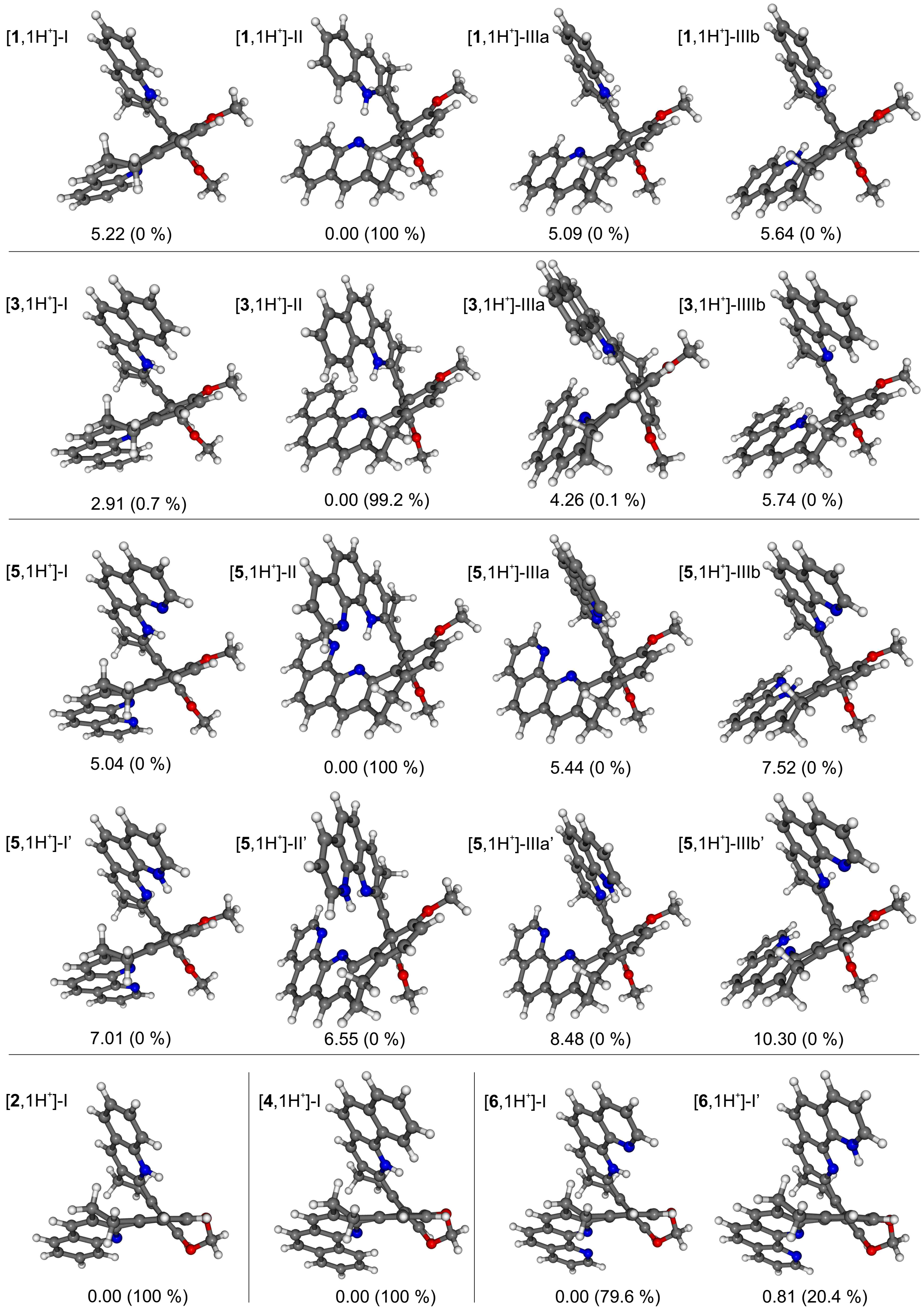

Figure S2.2. Selected low-energy DFT-optimized mono-protonated structures of (+)-enantiomers of **1**–**6**. Numbers listed are relative energy values with respect to the lowest-energy conformer (in kcal mol^{-1}) and the corresponding Boltzmann populations at 298 K (provided in parentheses). BLYP/TZVP/COSMO(CH_3CN) calculations.

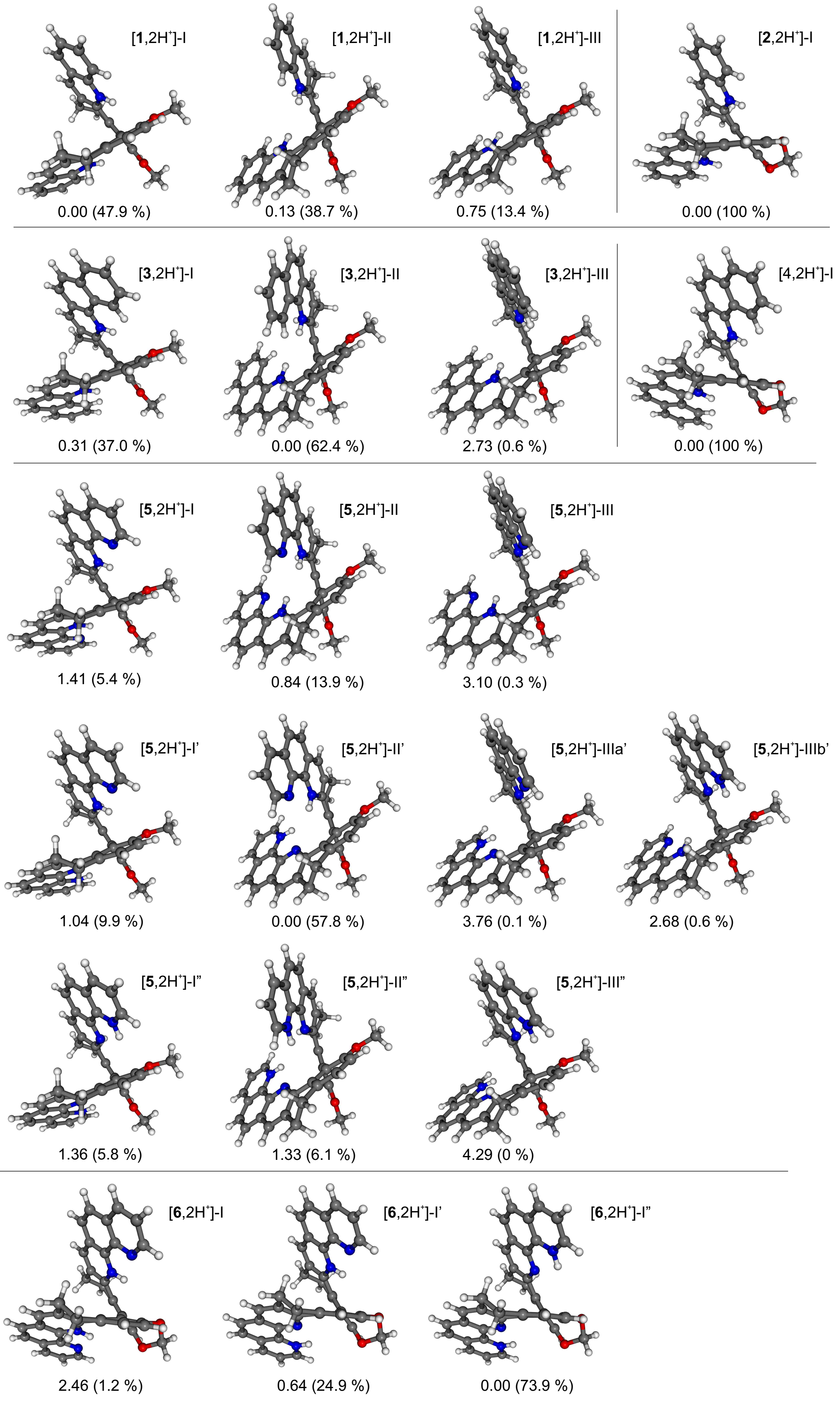

Figure S2.3. Selected low-energy DFT-optimized double-protonated structures of (+)-enantiomers of **1**–**6**. Numbers listed are relative energy values with respect to the lowest-energy conformer (in kcal mol^{-1}) and the corresponding Boltzmann populations at 298 K (provided in parentheses). BLYP/TZVP/COSMO(CH_3CN) calculations.

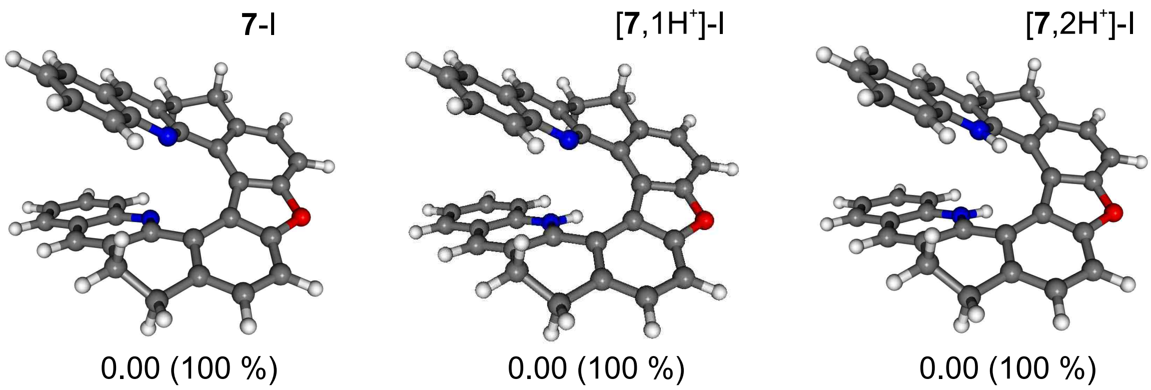

Figure S2.4. Selected low-energy DFT-optimized structures of (+)-enantiomer of **7** in its neutral and protonated forms. Numbers listed are relative energy values with respect to the lowest-energy conformer (in kcal mol^{-1}) and the corresponding Boltzmann populations at 298 K (provided in parentheses). BLYP/TZVP/COSMO(CH_3CN) calculations.

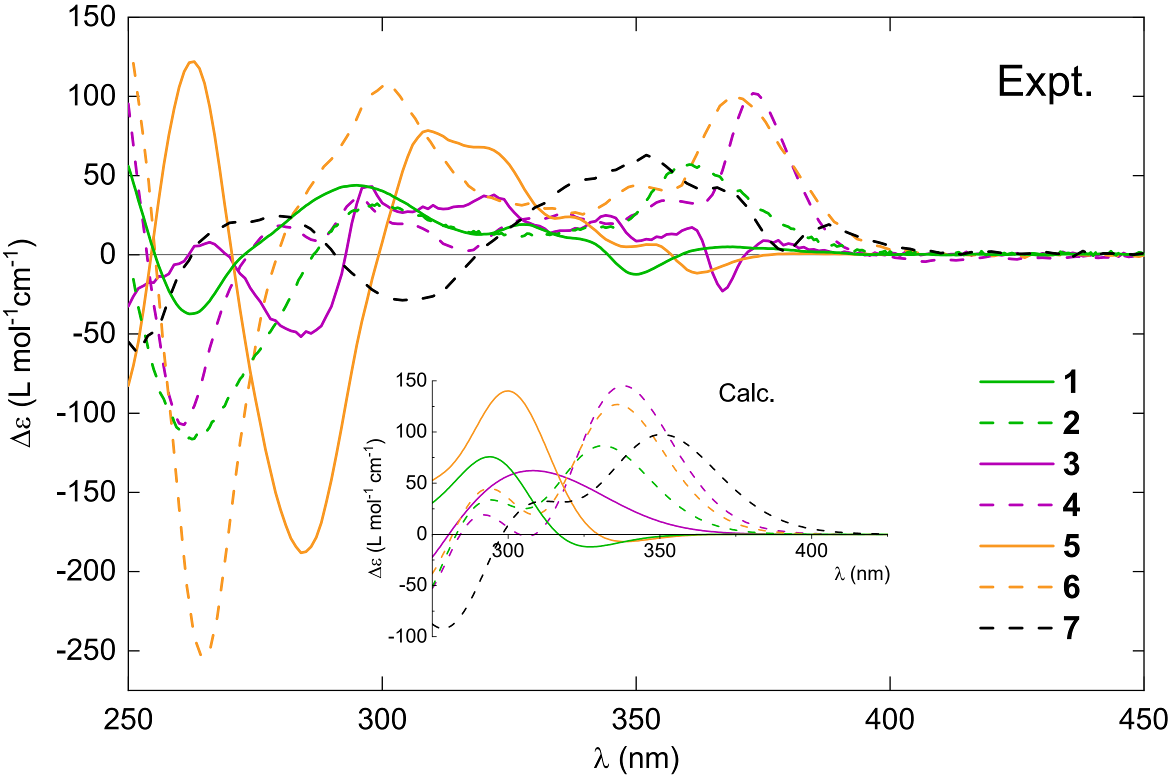

Figure S2.5. Experimental ECD spectra of (+)-enantiomers of **1**–**7** in their neutral forms. Inset: Corresponding TDDFT-simulated Boltzmann-averaged (at 298 K) ECD spectra. No spectral shifts were applied. LC-PBE0*/SV(P)/PCM(CH_3CN) calculations. Data for **1**–**6** taken from ref. [20].

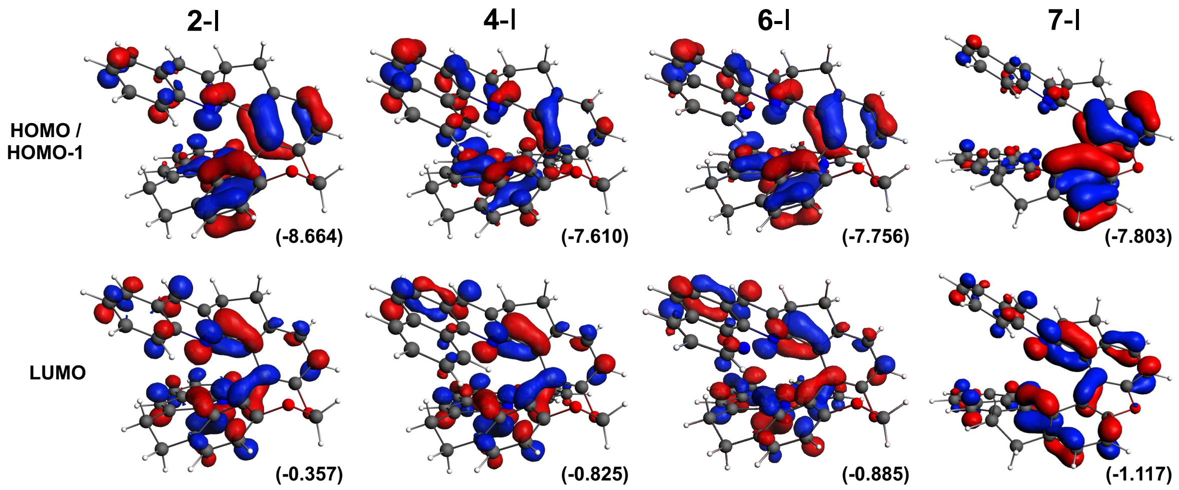

Figure S2.6. Isosurfaces (±0.04 au) of HOMO and LUMO for (+)-enantiomers of **2**, **4**, and **6** as well as of HOMO-1 and LUMO for (+)-enantiomer of **7** in their neutral forms. Values listed in parentheses are orbital energies (in eV). LC-PBE0*/SV(P)/PCM(CH_3CN) calculations. Data for **2**, **4**, and **6** taken from ref. [20].

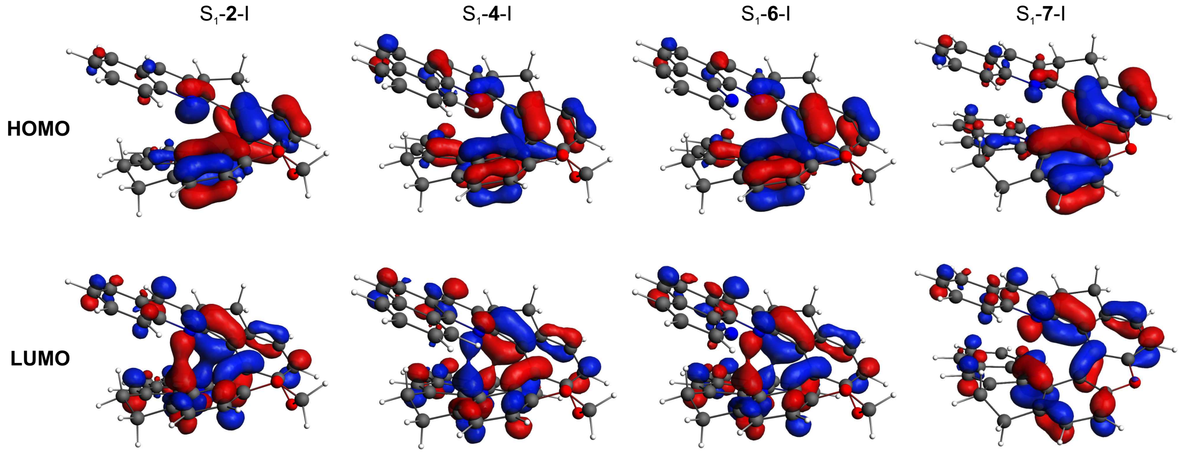

Figure S2.7. Isosurfaces (±0.04 au) of frontier MOs of S_0 at S_1 excited-state geometry involved in the $S_1 \rightarrow S_0$ emission transition for (+)-enantiomers of neutral species of **2**, **4**, **6**, and **7**. LC-PBE0*/SV(P)/PCM(CH_2Cl_2) calculations. Data for **2**, **4**, and **6** taken from ref. [20].

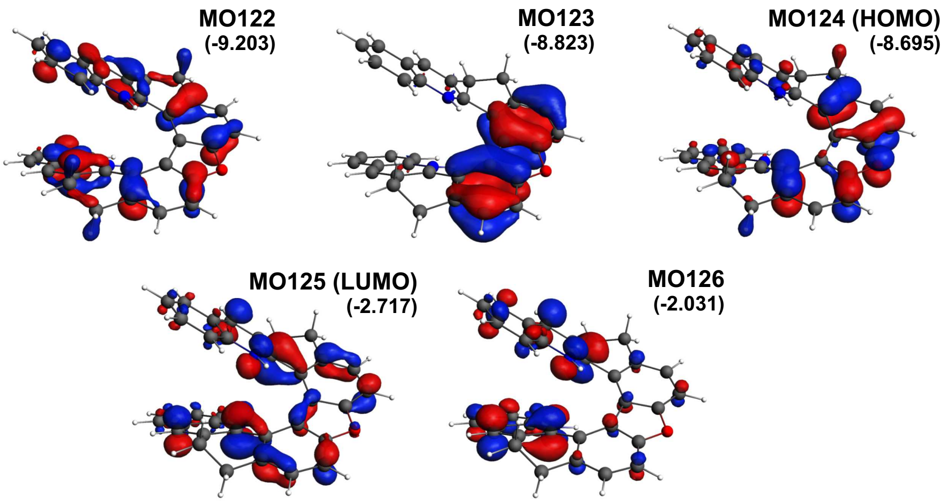

Figure S2.8. Isosurfaces (±0.04 au) of MOs involved in selected electronic transitions for (+)-[**7**,2H$^+$]-I. Values listed in parentheses are orbital energies (in eV). LC-PBE0*/SV(P)/PCM(CH_3CN) calculations.

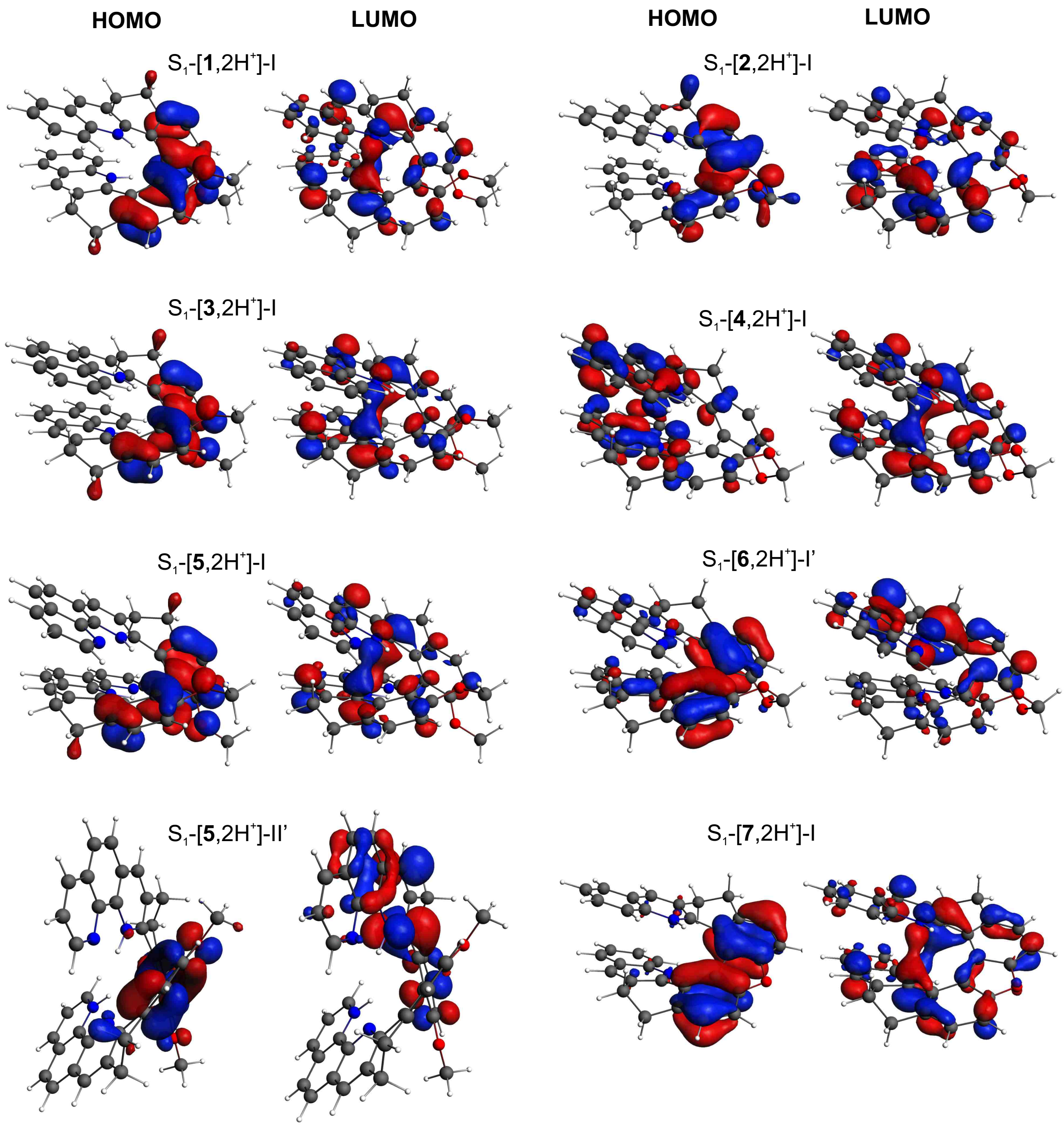

Figure S2.9. Isosurfaces (±0.04 au) of frontier MOs of S_0 at S_1 excited-state geometry involved in the $S_1 \rightarrow S_0$ emission transition for (+)-enantiomers of double-protonated forms of **1**–**7** in their most populated structures. LC-PBE0*/SV(P)/PCM(CH_2Cl_2) calculations.

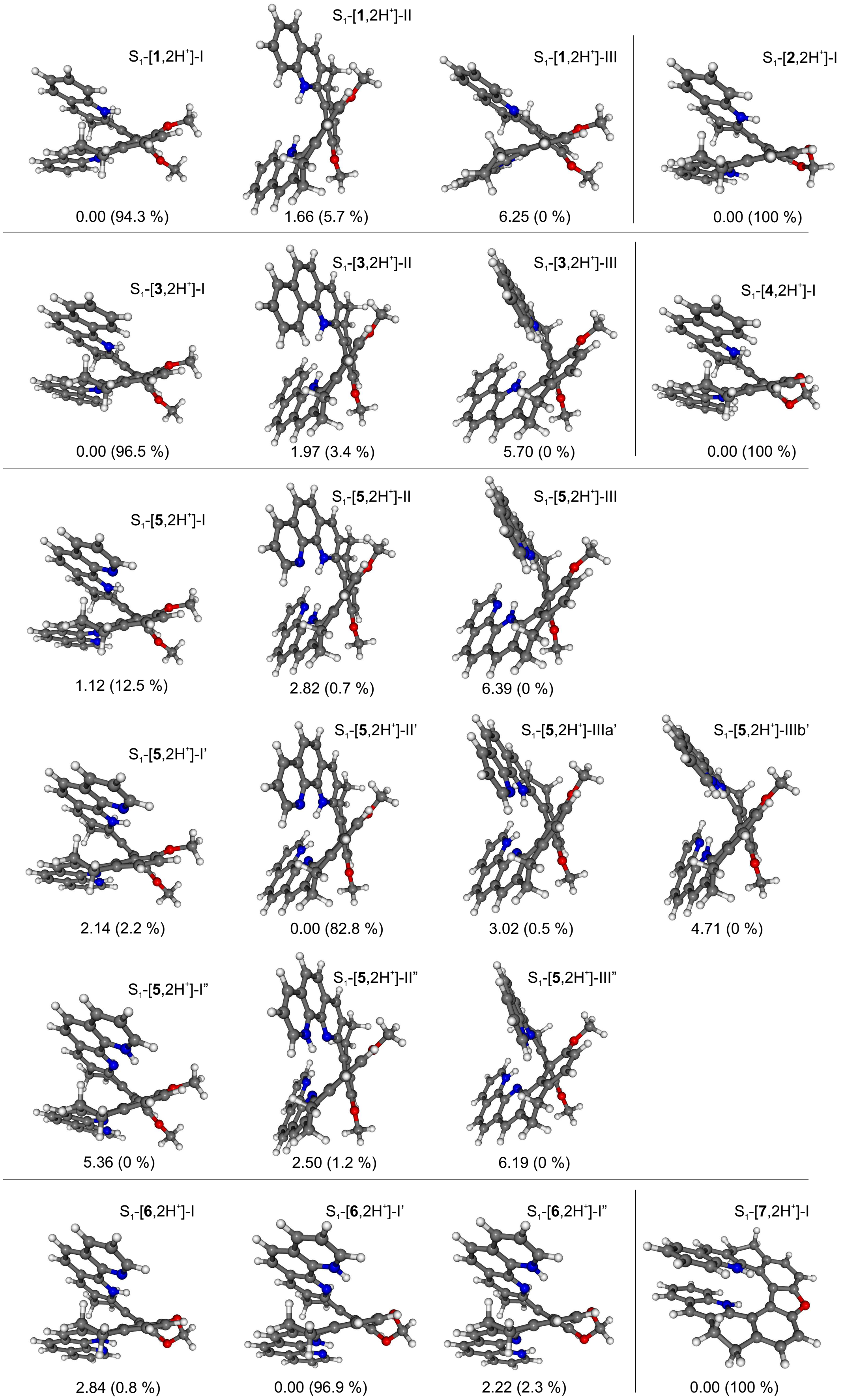

Figure S2.10. TDDFT-optimized structures of (+)-enantiomers of double-protonated forms of **1**–**7** in the S_1 excited state. Numbers listed are relative energy values with respect to the lowest-energy conformer (in kcal mol^{-1}) and the corresponding Boltzmann populations at 298 K (provided in parentheses). LC-PBE0*/SV(P)/PCM(CH_2Cl_2) calculations.

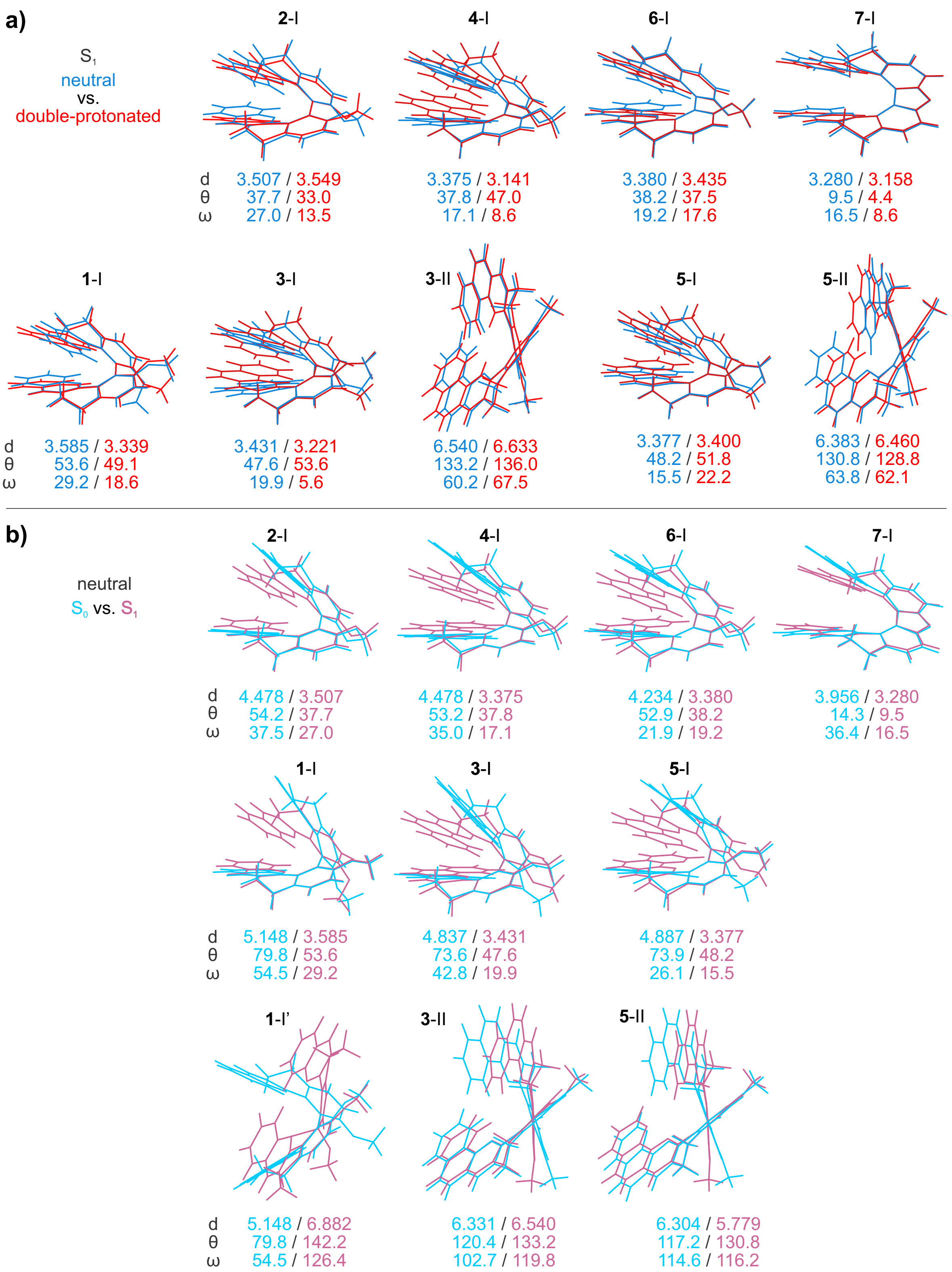

Figure S2.11. Overlays of optimized structures of (+)-enantiomers of neutral (blue) *vs.* double-protonated (red) forms of **1–7** in the S_1 excited state (panel a) and the ground (cyan) *vs.* S_1 excited (pink) states of neutral forms of **1–7** (panel b). Numbers listed are distances d (in Å) between the inner pyridine ring centroids of each heterocycle subunit, biphenyl's torsional angles θ (in deg) and angles between two aromatic subunits' planes ω (in deg). BLYP/TZVP/COSMO(CH_2Cl_2) and LC-PBE0*/SV(P)/PCM(CH_2Cl_2) calculations for the ground and S_1 excited state, respectively. Data for the neutral forms of **1–6** taken from ref. [20].

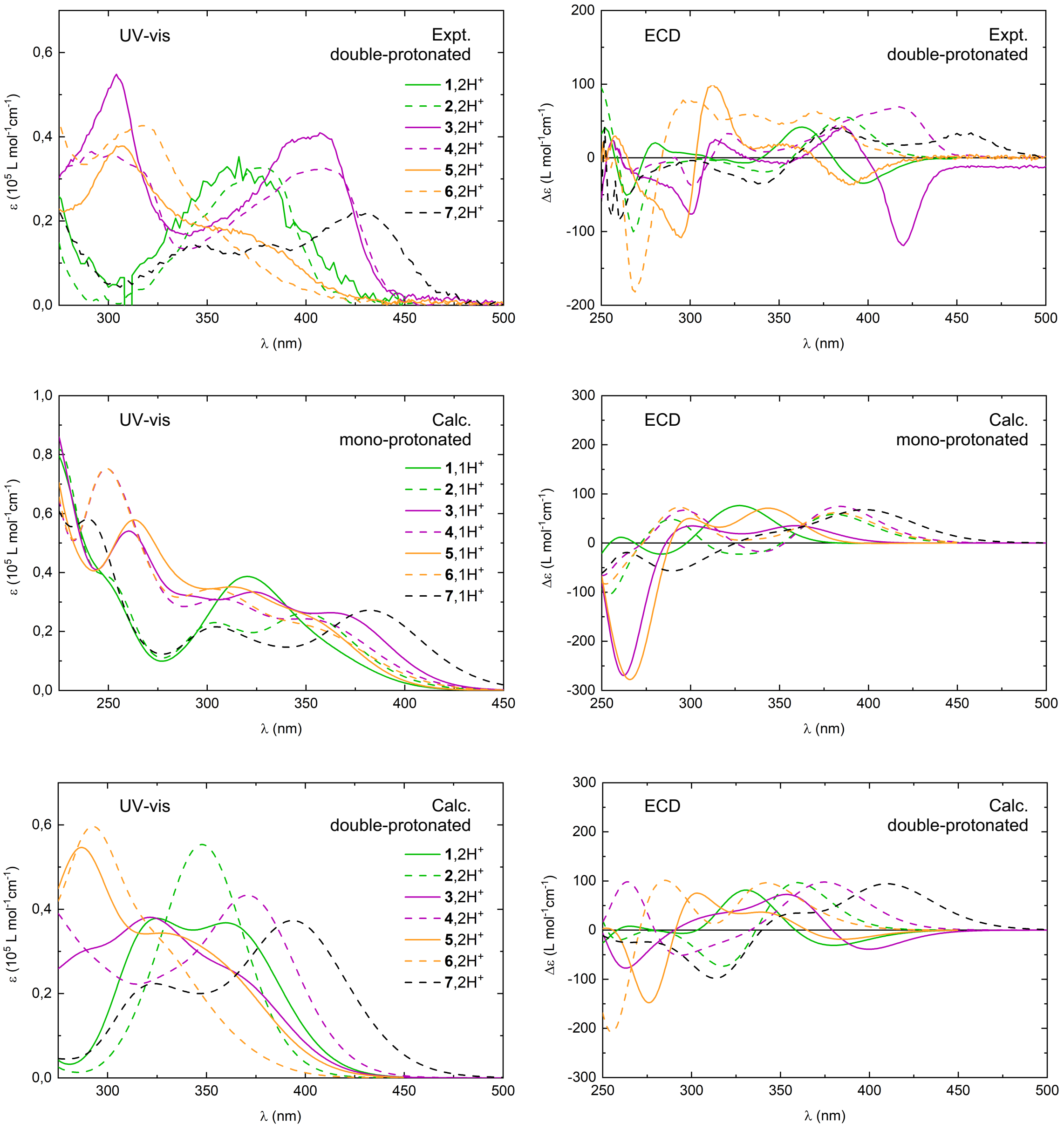

Figure S2.12. Comparison of experimental and TDDFT-simulated Boltzmann-averaged (at 298 K) UV-vis and ECD spectra for (+)-enantiomers of protonated species of **1**–**7**. No spectral shifts were applied. LC-PBE0*/SV(P)/PCM(CH_3CN) calculations.

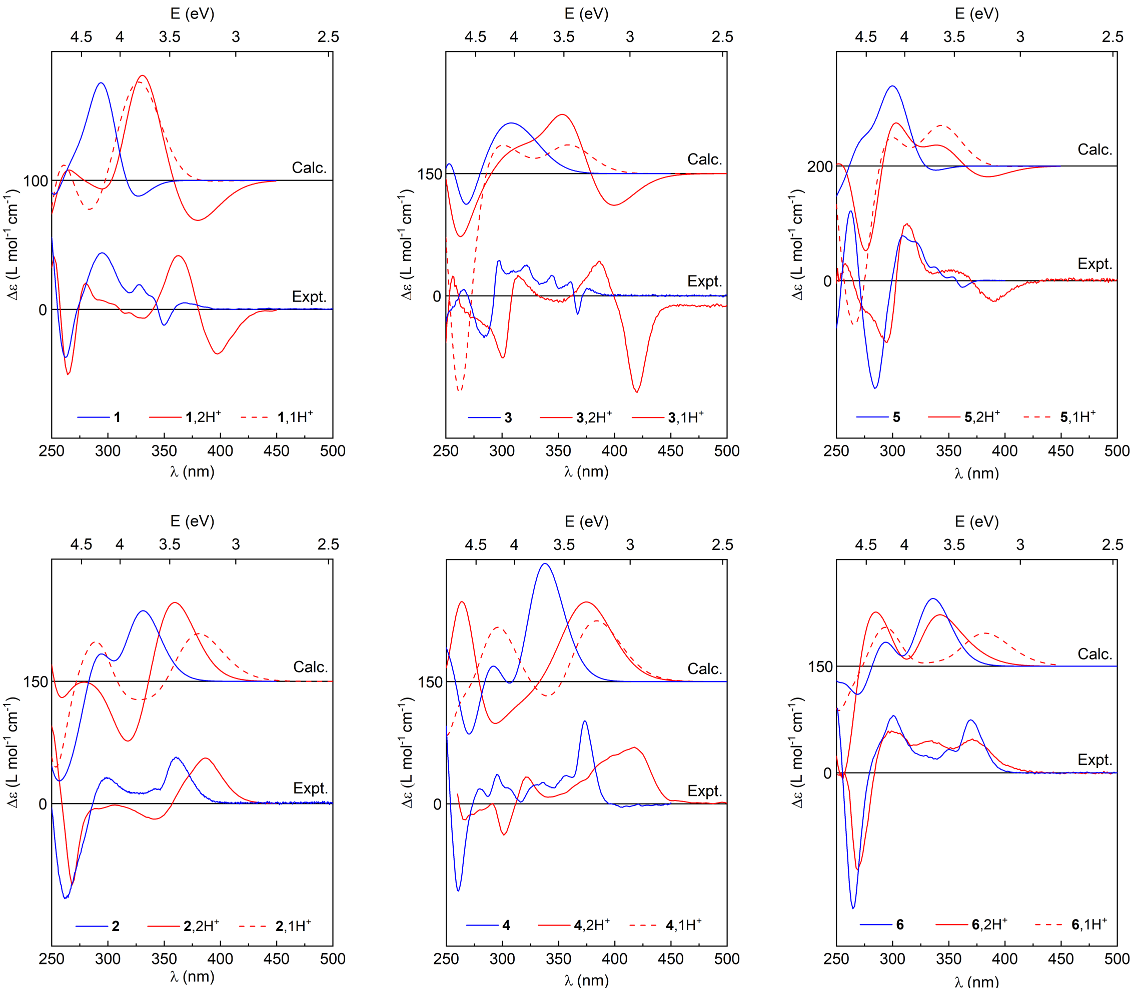

Figure S2.13. Comparison of experimental and TDDFT-simulated Boltzmann-averaged (at 298 K) ECD spectra for (+)-enantiomers of neutral and protonated forms of **1**–**6**. No spectral shifts were applied. LC-PBE0*/SV(P)/PCM(CH_3CN) calculations. Data for the neutral species of **1**–**6** taken from ref. [20].

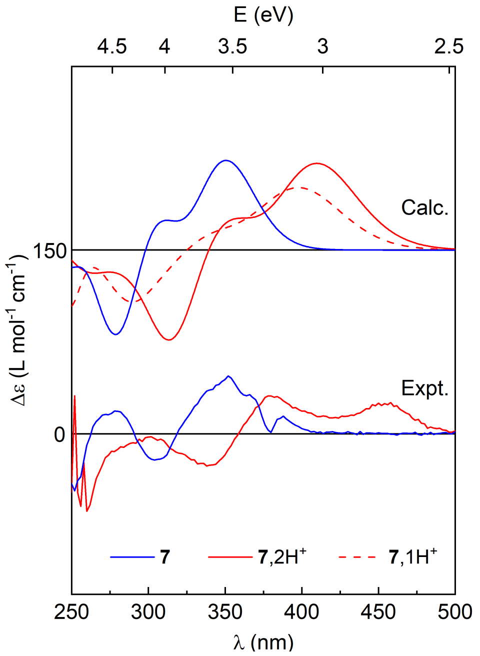

Figure S2.14. Comparison of experimental and TDDFT-simulated Boltzmann-averaged (at 298 K) ECD spectra for (+)-enantiomer of neutral and protonated forms of **7**. No spectral shifts were applied. LC-PBE0*/SV(P)/PCM(CH_3CN) calculations.

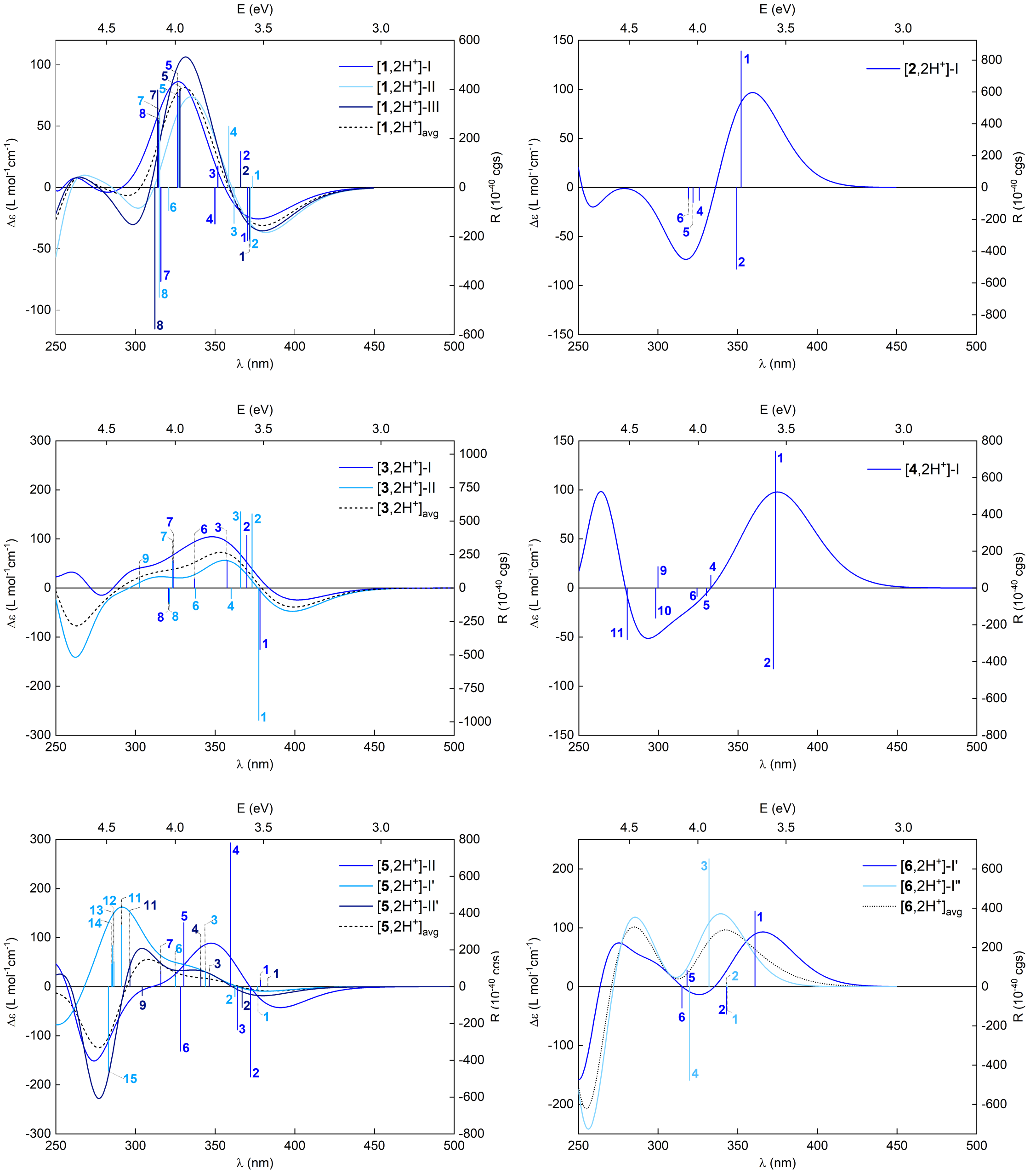

Figure S2.15. TDDFT-simulated ECD spectra for the lowest-energy conformers (>10 %) of double-protonated forms of (+)-**1**–(+)-**6** along with the corresponding Boltzmann-averaged (at 298 K) spectra. Selected, analyzed in detail, calculated excitations (energies and rotatory strengths) indicated as 'stick' spectra. No spectral shifts were applied. LC-PBE0*/SV(P)/PCM(CH_3CN) calculations.

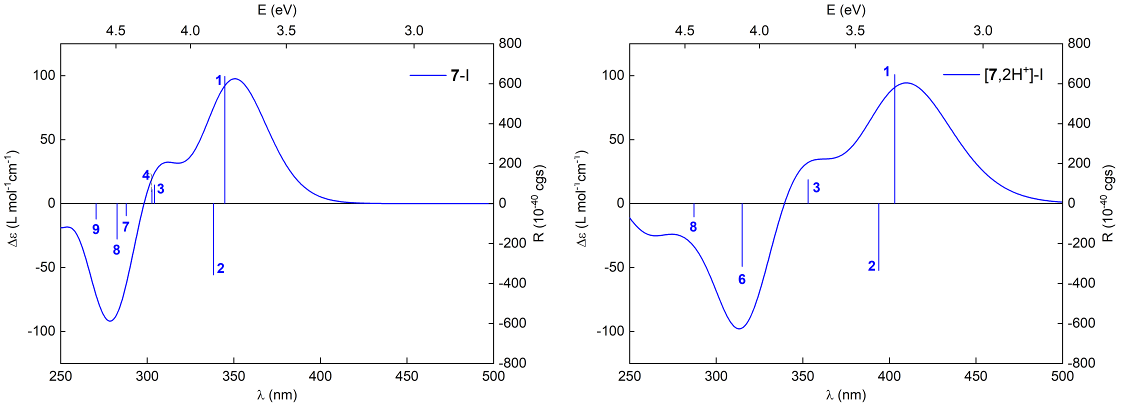

Figure S2.16. TDDFT-simulated ECD spectra for the lowest-energy conformers (>10 %) of (+)-**7** in its neutral and double-protonated forms. Selected, analyzed in detail, calculated excitations (energies and rotatory strengths) indicated as 'stick' spectra. No spectral shifts were applied. LC-PBE0*/SV(P)/PCM(CH_3CN) calculations.

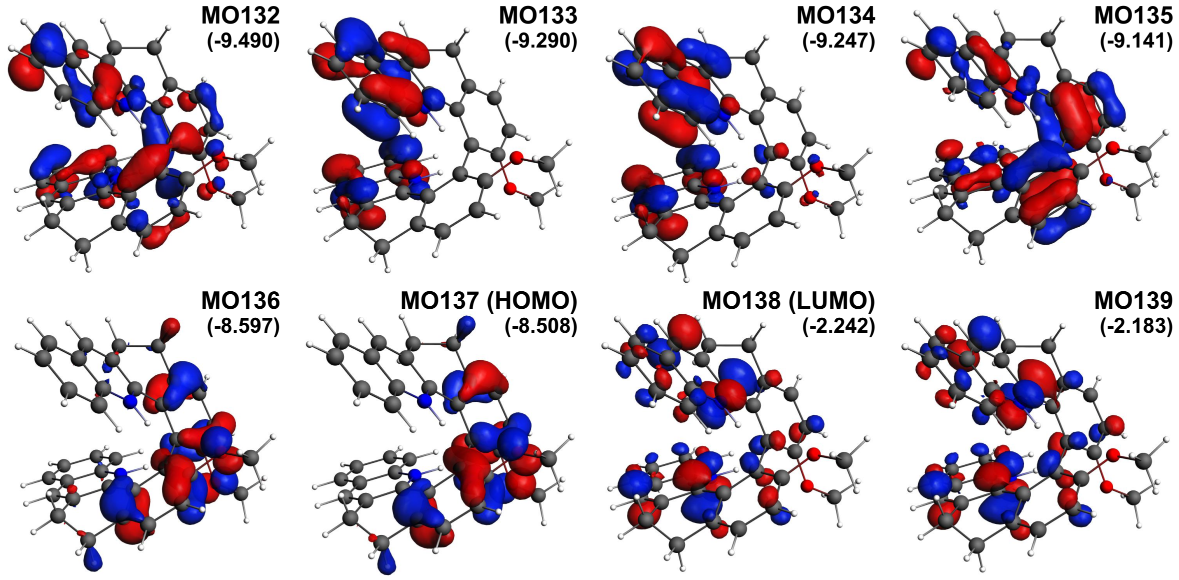

Figure S2.17. Isosurfaces (±0.04 au) of MOs involved in selected electronic transitions for (+)-[**1**,$2H^+$]-I. Values listed in parentheses are orbital energies (in eV). LC-PBE0*/SV(P)/PCM(CH_3CN) calculations.

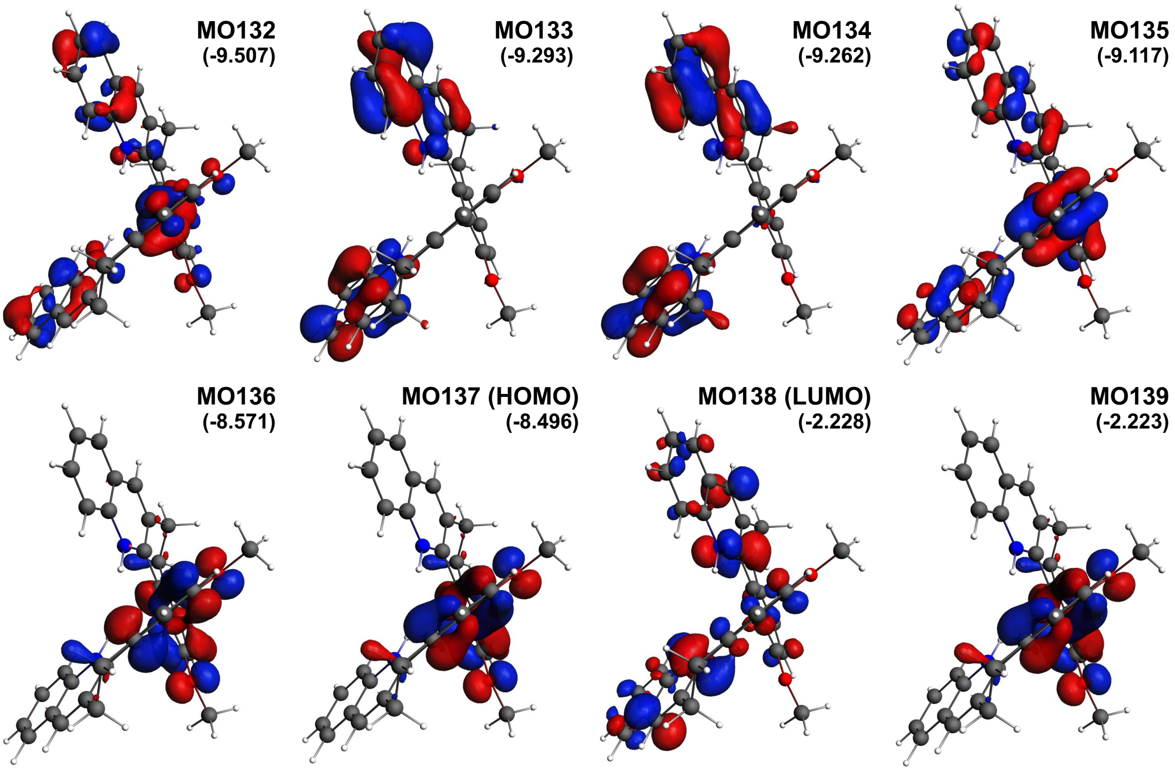

Figure S2.18. Isosurfaces (±0.04 au) of MOs involved in selected electronic transitions for (+)-[**1**,2H$^+$]-II. Values listed in parentheses are orbital energies (in eV). LC-PBE0*/SV(P)/PCM(CH_3CN) calculations.

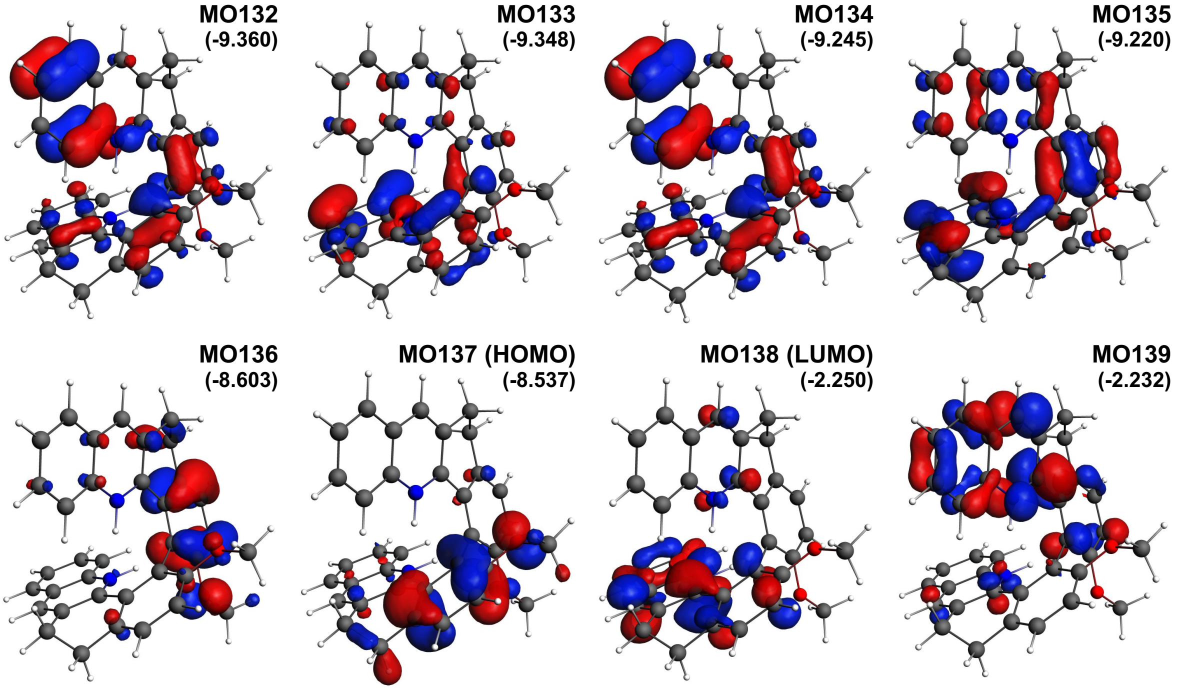

Figure S2.19. Isosurfaces (±0.04 au) of MOs involved in selected electronic transitions for (+)-[**1**,2H$^+$]-III. Values listed in parentheses are orbital energies (in eV). LC-PBE0*/SV(P)/PCM(CH_3CN) calculations.

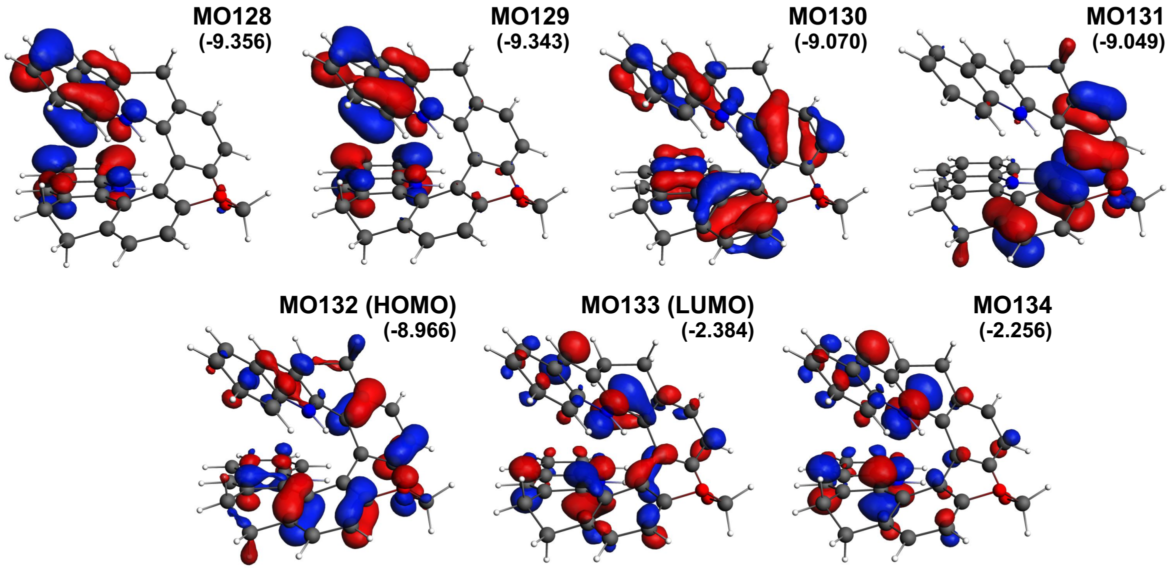

Figure S2.20. Isosurfaces (±0.04 au) of MOs involved in selected electronic transitions for (+)-[**2**,2H$^+$]-I. Values listed in parentheses are orbital energies (in eV). LC-PBE0*/SV(P)/PCM(CH_3CN) calculations.

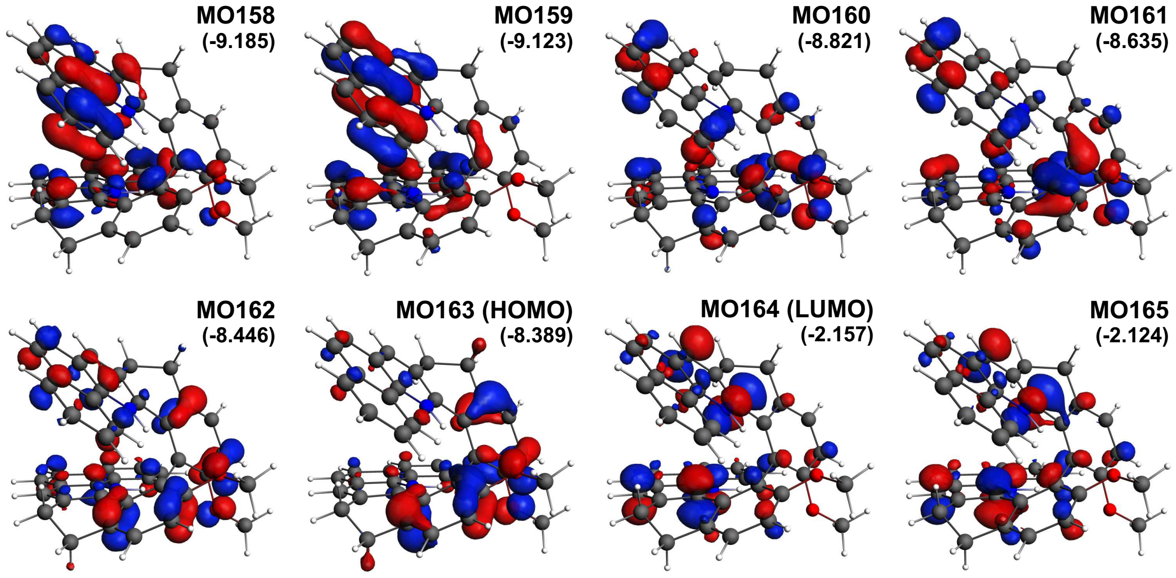

Figure S2.21. Isosurfaces (±0.04 au) of MOs involved in selected electronic transitions for (+)-[**3**,2H$^+$]-I. Values listed in parentheses are orbital energies (in eV). LC-PBE0*/SV(P)/PCM(CH_3CN) calculations.

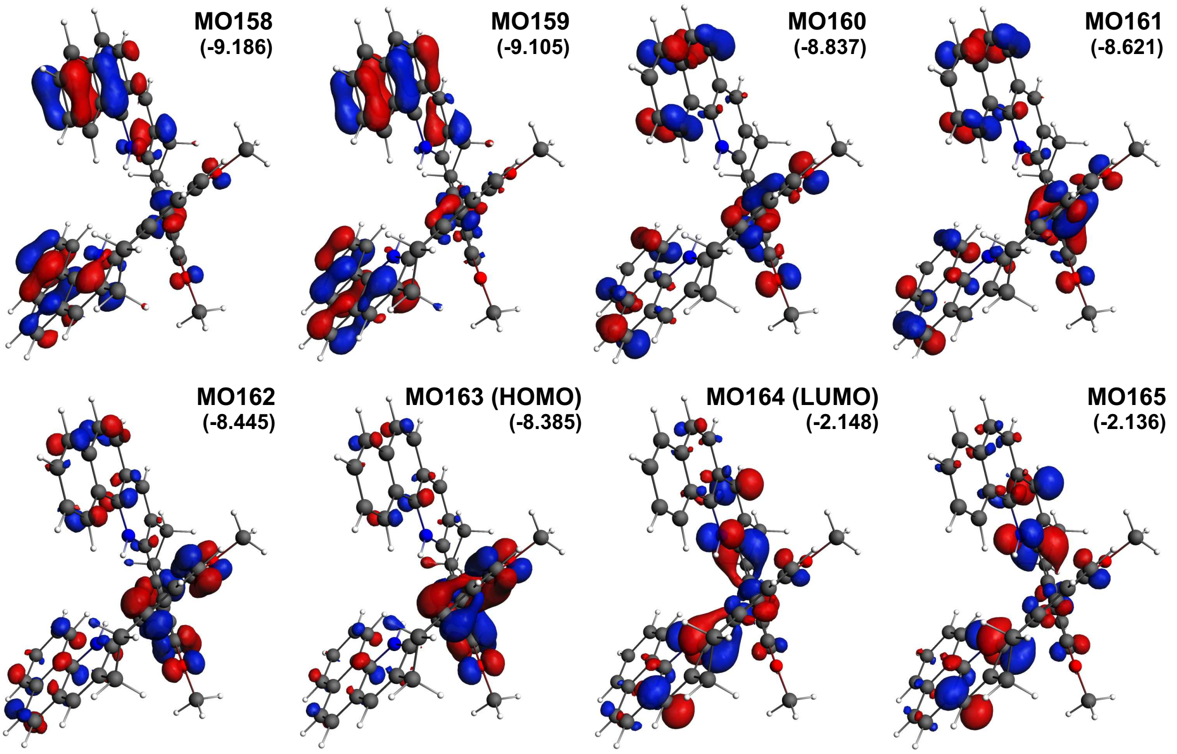

Figure S2.22. Isosurfaces (±0.04 au) of MOs involved in selected electronic transitions for (+)-[**3**,2H$^+$]-II. Values listed in parentheses are orbital energies (in eV). LC-PBE0*/SV(P)/PCM(CH_3CN) calculations.

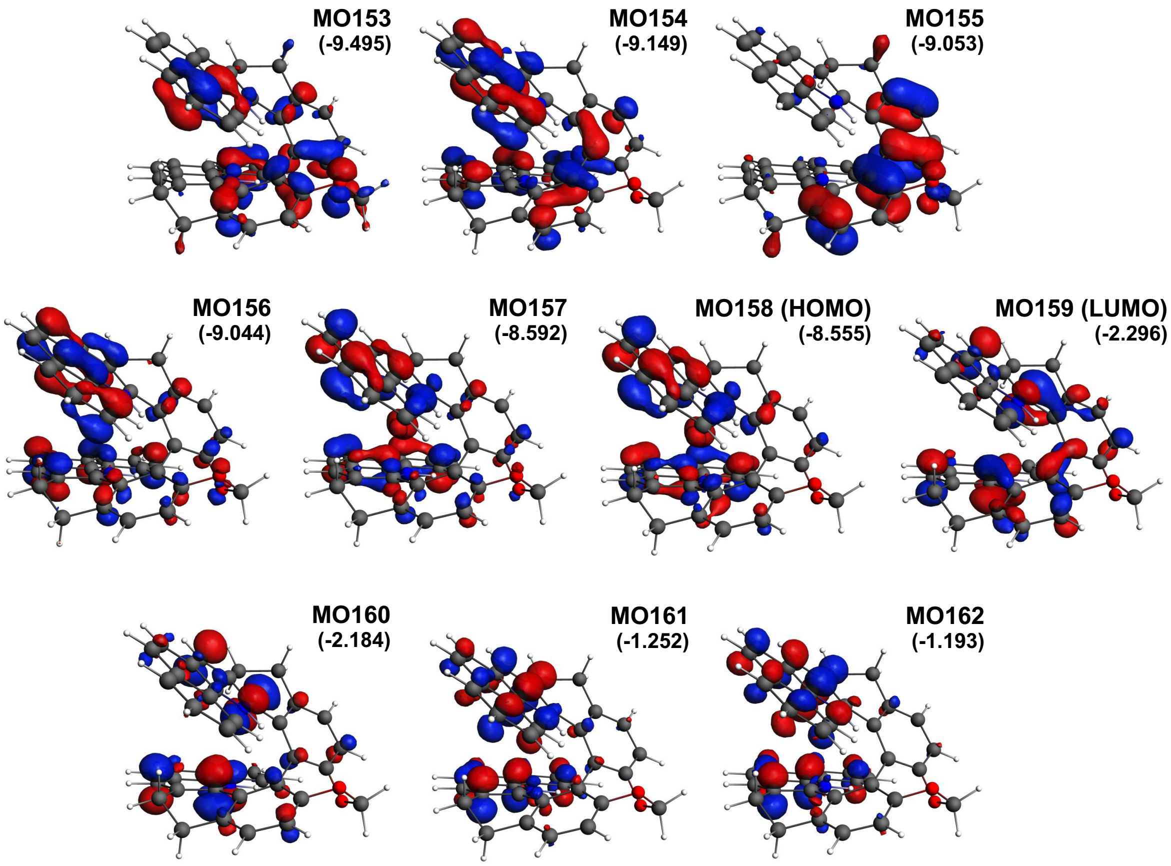

Figure S2.23. Isosurfaces (±0.04 au) of MOs involved in selected electronic transitions for (+)-[**4**,2H$^+$]-I. Values listed in parentheses are orbital energies (in eV). LC-PBE0*/SV(P)/PCM(CH_3CN) calculations.

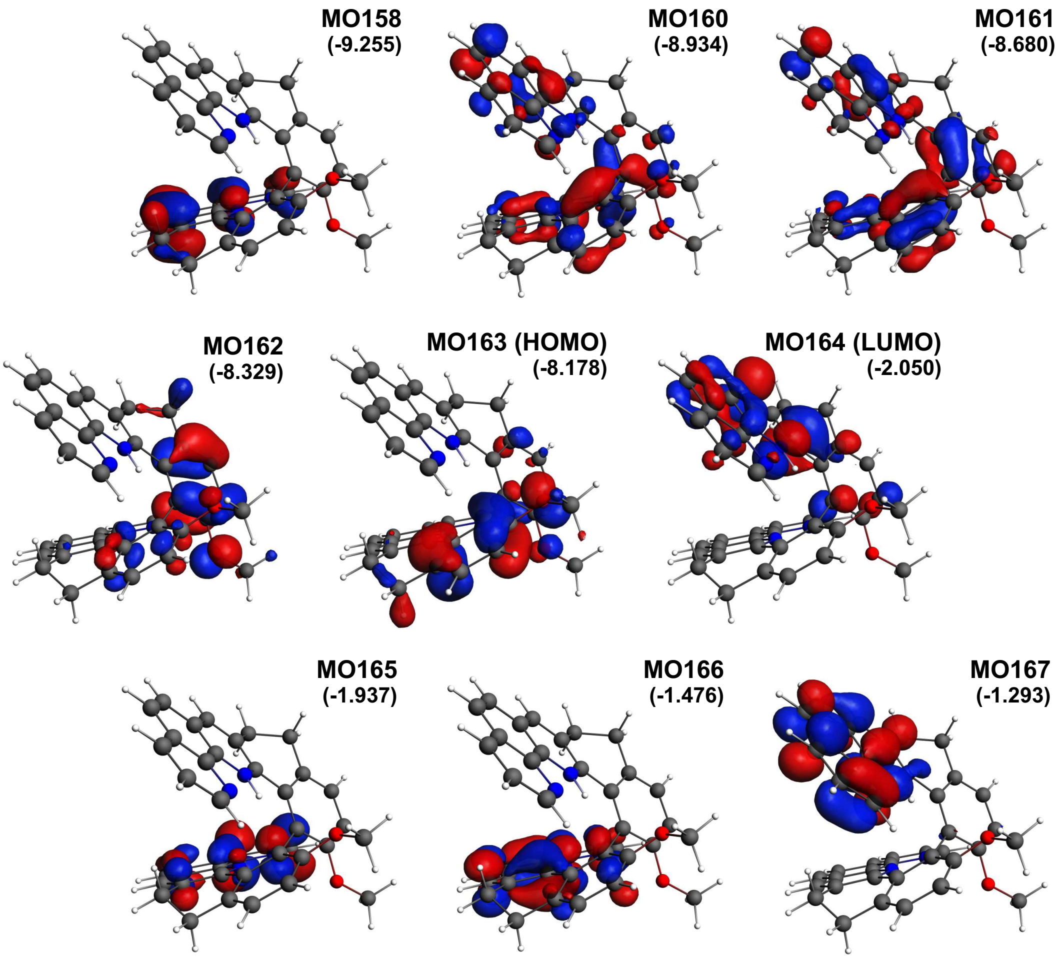

Figure S2.24. Isosurfaces (±0.04 au) of MOs involved in selected electronic transitions for (+)-[**5**,2H$^+$]-I′. Values listed in parentheses are orbital energies (in eV). LC-PBE0*/SV(P)/PCM(CH_3CN) calculations.

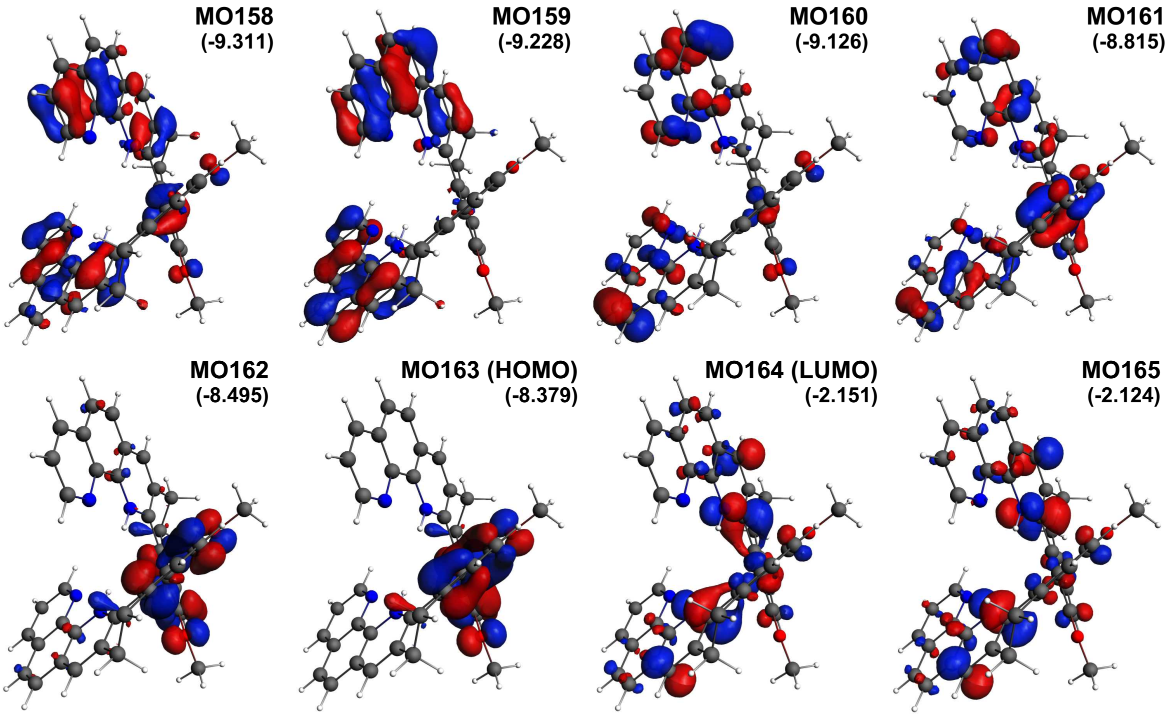

Figure S2.25. Isosurfaces (±0.04 au) of MOs involved in selected electronic transitions for (+)-[**5**,2H$^+$]-II. Values listed in parentheses are orbital energies (in eV). LC-PBE0*/SV(P)/PCM(CH_3CN) calculations.

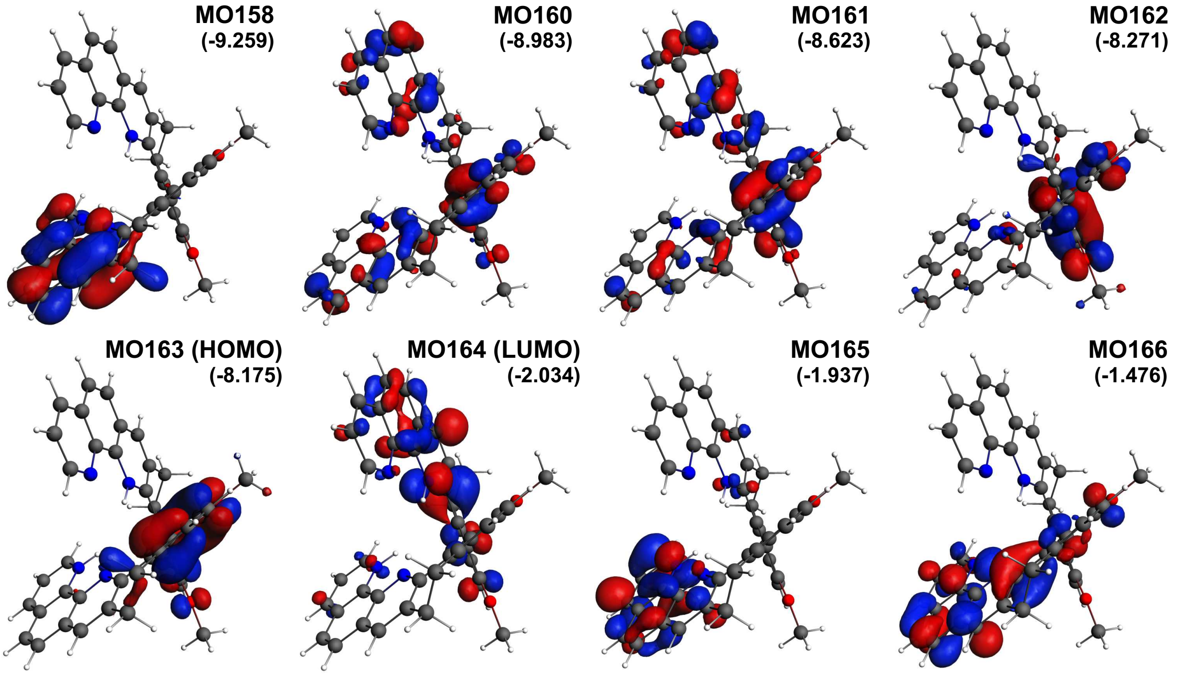

Figure S2.26. Isosurfaces (±0.04 au) of MOs involved in selected electronic transitions for (+)-[**5**,2H$^+$]-II′. Values listed in parentheses are orbital energies (in eV). LC-PBE0*/SV(P)/PCM(CH_3CN) calculations.

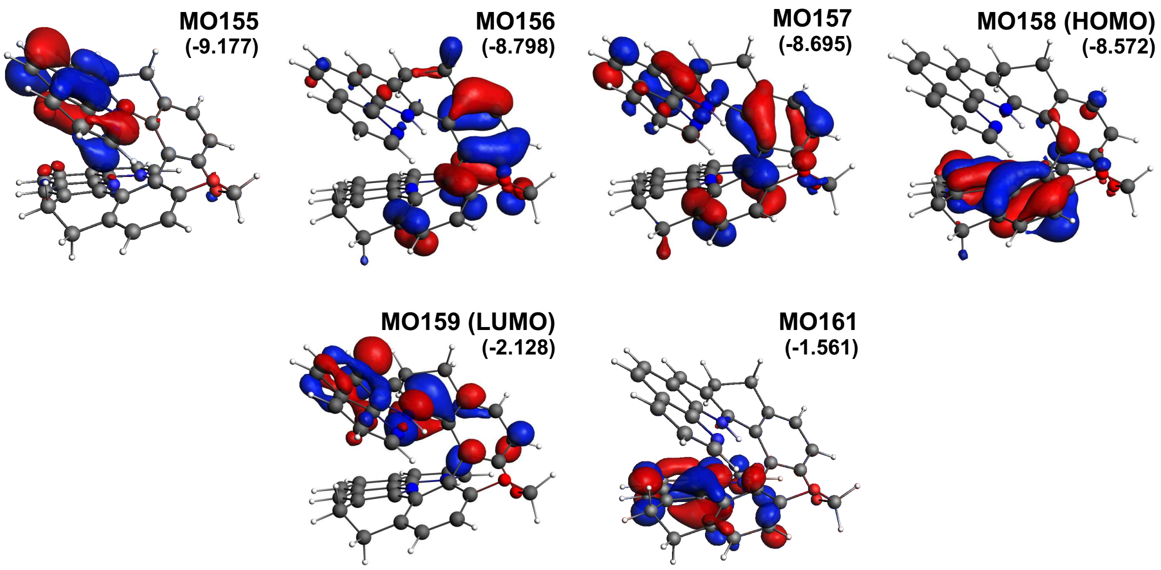

Figure S2.27. Isosurfaces (±0.04 au) of MOs involved in selected electronic transitions for (+)-[**6**,2H$^+$]-I′. Values listed in parentheses are orbital energies (in eV). LC-PBE0*/SV(P)/PCM(CH_3CN) calculations.

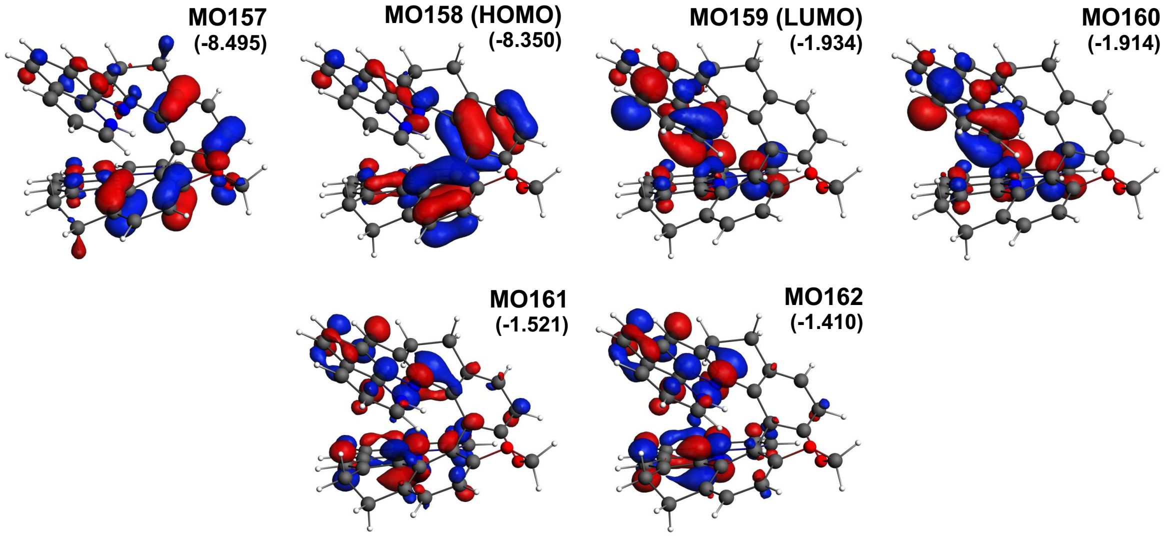

Figure S2.28. Isosurfaces (±0.04 au) of MOs involved in selected electronic transitions for (+)-[**6**,2H$^+$]-I″. Values listed in parentheses are orbital energies (in eV). LC-PBE0*/SV(P)/PCM(CH_3CN) calculations.

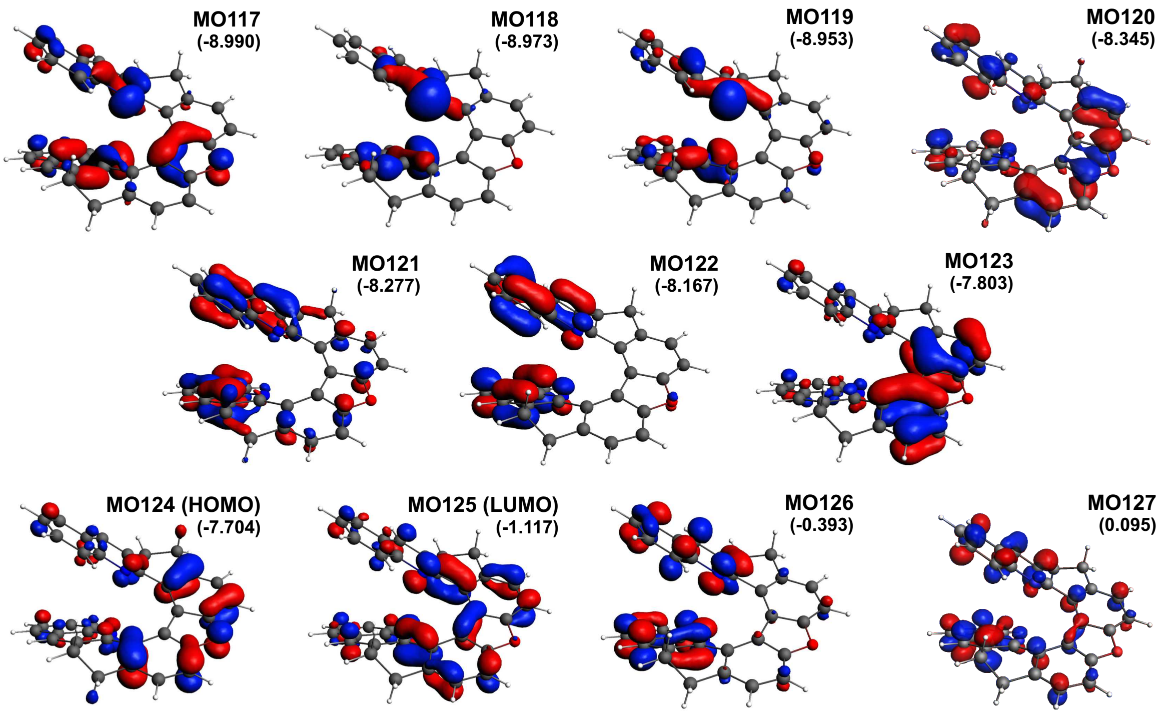

Figure S2.29. Isosurfaces (±0.04 au) of MOs involved in selected electronic transitions for (+)-**7**-I. Values listed in parentheses are orbital energies (in eV). LC-PBE0*/SV(P)/PCM(CH_3CN) calculations.

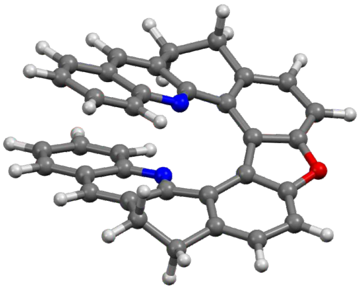

Figure S2.30. TDDFT-optimized structure of (+)-**7**-I in the S_1 excited state. LC-PBE0*/SV(P)/PCM(CH_2Cl_2) calculations.

Table S2.1. Experimental and calculated (LC-PBE0*/SV(P)/PCM(CH_2Cl_2)) photophysical data for (+)-enantiomers of double-protonated forms of **1**–**6** and of neutral and double-protonated species of **7** in the S_1 excited state. ΔE (n_B) – relative energy values calculated with respect to the lowest-energy conformer (in kcal mol^{-1}) when more than one conformer is populated along with the corresponding Boltzmann populations (at 298 K, in %) listed in parentheses. $\lambda^{calc.}$ – S_1-S_0 energy difference (in nm) at TDDFT-optimized S_1 geometry. f – oscillator strength. R – rotatory strength (in 10^{-40} cgs). $g_{lum}^{calc.}$ – emission dissymmetry factor computed as 4R/D, where D is dipole strength. LUMO→HOMO (L→H) MO-pair contributions to $S_1 \to S_0$ emission transition are also listed. For structures visualization, see Figures S2.10 and S2.30. For MOs isosurfaces, see Figures S2.9 and S2.7. For additional data, see Table S2.2.

Structure	ΔE	(n_B)	$\lambda^{calc.}$	$\lambda^{expt.}$	f	R	$g_{lum}^{calc.}$	$g_{lum}^{expt.}$	L→H %
S_1-[**1**,2H$^+$]-I	0.00	(94.3)	529		0.1149	-2.28	-0.0001		95.1
S_1-[**1**,2H$^+$]-II	1.66	(5.7)	502		0.0409	-41.68	-0.0038		95.3
S_1-[**1**,2H$^+$]-III	6.25	(0)	487		0.1528	-17.08	-0.0004		94.9
S_1-[**1**,2H$^+$]†				545			-0.0003	–0.001	
S_1-[**2**,2H$^+$]-I			489		0.1337	3.32	0.0001		92.0
S_1-[**2**,2H$^+$]†				550			0.0001	0.0025	
S_1-[**3**,2H$^+$]-I	0.00	(96.5)	506		0.1570	-22.87	-0.0005		92.7
S_1-[**3**,2H$^+$]-II	1.97	(3.4)	486		0.0326	-76.76	-0.0091		92.4
S_1-[**3**,2H$^+$]-III	5.70	(0)	432		0.5634	-164.22	-0.0013		91.5
S_1-[**3**,2H$^+$]†				480			-0.0008	–0.0016	
S_1-[**4**,2H$^+$]-I			480		0.3259	680.63	0.0082		90.7
S_1-[**4**,2H$^+$]†				480			0.0082	0.002	
S_1-[**5**,2H$^+$]-I	1.12	(12.5)	520		0.1191	-12.65	-0.0004		94.4
S_1-[**5**,2H$^+$]-II	2.82	(0.7)	491		0.0383	-55.67	-0.0056		94.7
S_1-[**5**,2H$^+$]-III	6.39	(0)	473		0.0734	-46.01	-0.0025		93.8
S_1-[**5**,2H$^+$]-I′	2.14	(2.2)	487		0.0493	27.67	0.0022		93.1
S_1-[**5**,2H$^+$]-II′	0.00	(82.8)	509		0.0082	-42.47	-0.0191		94.9
S_1-[**5**,2H$^+$]-IIIa′	3.02	(0.5)	491		0.0416	-72.79	-0.0067		93.6
S_1-[**5**,2H$^+$]-IIIb′	4.71	(0)	500		0.0101	-41.35	-0.0154		94.0
S_1-[**5**,2H$^+$]-I″	5.36	(0)	487		0.0112	6.96	0.0024		92.1
S_1-[**5**,2H$^+$]-II″	2.50	(1.2)	519		0.0062	3.93	0.0023		89.3
S_1-[**5**,2H$^+$]-III″	6.19	(0)	507		0.0036	-0.45	-0.0005		88.5
S_1-[**5**,2H$^+$]†				550			-0.0159	0	
S_1-[**6**,2H$^+$]-I	2.84	(0.8)	462		0.1099	652.95	0.0242		95.6
S_1-[**6**,2H$^+$]-I′	0.00	(96.9)	475		0.1055	462.24	0.0173		96.0
S_1-[**6**,2H$^+$]-I″	2.22	(2.3)	467		0.0637	435.58	0.0275		83.7<br>11.3‡
S_1-[**6**,2H$^+$]†				550			0.0176	0.002	
S_1-**7**-I			434		0.0923	517.19	0.0243		95.8
S_1-**7**†				440			0.0243	0.014	
S_1-[7,2H$^+$]-I			516		0.0888	459.98	0.0189		95.0
S_1-[7,2H$^+$]†				560			0.0189	0.014	

† Boltzmann-averaged values (at 298 K).
‡ LUMO+1→HOMO contribution.

Table S2.2. Parameters corresponding to the $S_1 \rightarrow S_0$ emission transition calculated (LC-PBE0*/SV(P)/PCM(CH_2Cl_2)) for (+)-enantiomers of selected neutral and double-protonated species of **3**–**7** in the S_1 excited state. E – emission energy (in eV). f – oscillator strength. R – rotatory strength (in 10^{-40} cgs). D – dipole strength (in 10^{-40} cgs). g_{lum} – emission dissymmetry factor computed as 4R/D. $|\mathbf{d}|$ and $|\mathbf{m}|$ – magnitudes of respectively electric and magnetic transition dipole moment vector (in au). φ – angle between **d** and **m** vectors (in deg). Data for the neutral structures of **1**–**6** taken from ref. [20].

Structure	E	f	R	D	g_{lum}	$\|\mathbf{m}\|$	$\|\mathbf{d}\|$	φ
S_1-**2**-I	2.447	0.031	281.44	33299	0.0338	1.753	0.718	18.48
S_1-[**2**,2H$^+$]-I	2.535	0.134	3.32	139089	0.0001	1.407	1.467	89.61
S_1-**4**-I	2.692	0.102	507.50	100024	0.0203	1.921	1.244	25.74
S_1-[**4**,2H$^+$]-I	2.582	0.326	680.63	332852	0.0082	1.707	2.270	41.80
S_1-**6**-I	2.609	0.069	416.17	69543	0.0239	1.849	1.038	23.06
S_1-[**6**,2H$^+$]-I′	2.609	0.106	462.24	106623	0.0173	1.961	1.285	38.90
S_1-**7**-I	2.858	0.092	517.19	85165	0.0243	2.492	1.148	39.94
S_1-[**7**,2H$^+$]-I	2.405	0.089	459.98	97386	0.0189	2.521	1.228	50.92
S_1-**1**-I	2.444	0.015	141.61	16613	0.0341	1.252	0.507	18.80
S_1-[**1**,2H$^+$]-I	2.343	0.115	-2.28	129295	-0.0001	0.888	1.415	90.44
S_1-**3**-I	2.718	0.054	373.38	52495	0.0285	1.783	0.901	9.59
S_1-[**3**,2H$^+$]-I	2.449	0.157	-22.87	169044	-0.0005	0.534	1.618	96.45
S_1-**3**-II	2.573	0.011	-44.26	11377	-0.0156	1.011	0.420	116.22
S_1-[**3**,2H$^+$]-II	2.552	0.033	-76.76	33682	-0.0091	1.050	0.722	115.44
S_1-**5**-I	2.630	0.040	312.96	40313	0.0311	1.692	0.790	6.55
S_1-[**5**,2H$^+$]-I	2.387	0.119	-12.65	131595	-0.0004	0.787	1.427	92.74
S_1-**5**-II	2.448	0.005	-20.04	5602	-0.0143	0.719	0.296	113.58
S_1-[**5**,2H$^+$]-II	2.435	0.008	-42.47	8880	-0.0191	0.948	0.372	120.73

Table S2.3. Experimental and calculated (LC-PBE0*/SV(P)/PCM(CH_2Cl_2)) molar rotations $[\Phi]_D$, in 10^{-1} deg mol^{-1} cm^2, for (+)-enantiomers of double-protonated forms of **1**–**7**. For structures visualization, see Figures S2.3 and S2.4. The corresponding values for the neutral species (for systems **1**–**6** taken from ref. [20]) are given in parentheses.

Structure	ΔE (kcal mol^{-1})		$[\Phi]_D^{calc.}$		$[\Phi]_D^{expt.}$	
[**1**,2H$^+$]-I	0.00	(0.00)	4039	(5495)		
[**1**,2H$^+$]-II	0.21	(0.33)	–2218	(–485)		
[**1**,2H$^+$]-III	0.76	(0.67)	1044	(2069)		
[**1**,2H$^+$]†			1407	(3097)	–2849	(4300)
[**2**,2H$^+$]-I	0.00	(0.00)	10313	(9156)		
[**2**,2H$^+$]†			10313	(9156)	10215	(8600)
[**3**,2H$^+$]-I	0.26	(0.01)	10827	(10853)		
[**3**,2H$^+$]-II	0.00	(0.00)	–6284	(–2939)		
[**3**,2H$^+$]-III	2.56	(3.23)	–241	(1321)		
[**3**,2H$^+$]†			450	(3885)	–6414	(1800)
[**4**,2H$^+$]-I	0.00	(0.00)	17472	(14649)		
[**4**,2H$^+$]†			17472	(14649)	22439	(13900)
[**5**,2H$^+$]-I	1.02	(0.00)	8536	(10198)		
[**5**,2H$^+$]-II	0.34	(0.57)	–4655	(-2961)		
[**5**,2H$^+$]-III	2.65	(3.02)	–842	(4816)		
[**5**,2H$^+$]-I′	0.94		9406			
[**5**,2H$^+$]-II′	0.00		–3725			
[**5**,2H$^+$]-IIIa′	3.68		1642			
[**5**,2H$^+$]-IIIb′	2.50		2838			
[**5**,2H$^+$]-I"	1.41		9282			
[**5**,2H$^+$]-II"	1.58		–4884			
[**5**,2H$^+$]-III"	4.25		3908			
[**5**,2H$^+$]†			–1097	(6543)	–3164	(5600)
[**6**,2H$^+$]-I	2.12	(0.00)	15586	(13475)		
[**6**,2H$^+$]-I′	0.55		15607			
[**6**,2H$^+$]-I"	0.00		14153			
[**6**,2H$^+$]†			14584	(13475)	12391	(14000)
[**7**,2H$^+$]-I	0.00	(0.00)	23817	(12535)		
[**7**,2H$^+$]†			23817	(12535)	18439	(7683)

† Boltzmann-averaged values (at 298 K).

Table S2.4. Selected dominant excitations and occupied (occ) - unoccupied (unocc) MO-pair contributions (greater than 10 %) for dominant double-protonated structures of (+)-**1**: [**1**,2H$^+$]-I, [**1**,2H$^+$]-II, and [**1**,2H$^+$]-III. For structures visualization, see Figure S2.3. For the corresponding simulated ECD spectra, see Figure S2.15. For MOs isosurfaces, see Figures S2.17–S2.19. LC-PBE0*/SV(P)/PCM(CH_3CN) calculations.

Excit. no.	E (eV)	λ (nm)	f	R (10^{-40} cgs)	occ no.	unocc no.	(%)
[**1**,2H$^+$]-I							
#1	3.35	370	0.1378	-216.28	137	138	75.2
					136	139	18.6
#2	3.39	366	0.3354	145.88	137	139	60.4
					136	138	32.7
#3	3.53	352	0.0550	86.70	136	138	57.4
					137	139	31.6
#4	3.54	350	0.0521	-149.12	136	139	68.6
					137	138	18.9
#5	3.80	326	0.2357	384.65	134	138	46.3
					133	139	28.9
					135	139	14.6
#7	3.92	316	0.0978	-382.88	135	138	72.7
					132	139	13.7
#8	3.94	314	0.1721	273.98	135	139	50.8
					132	138	20.5
					133	139	11.5
[**1**,2H$^+$]-II							
#1	3.32	373	0.1820	45.36	137	139	90.5
#2	3.33	372	0.1304	-240.13	137	138	63.8
					136	139	29.4
#3	3.43	362	0.0288	-146.18	136	139	59.7
					137	138	29.3
#4	3.46	359	0.2694	249.15	136	138	84.3
#5	3.79	327	0.3557	371.28	134	138	36.8
					135	139	27.7
					133	139	20.7
#6	3.86	321	0.0639	-94.18	134	139	43.4
					133	138	34.8
					135	138	14.8
#7	3.93	315	0.1079	306.93	135	139	35.8
					133	139	25.1
					132	138	17.6
					134	138	12.5
#8	3.94	315	0.1059	-447.42	135	138	53.9
					132	139	19.7
					133	138	13.8
[**1**,2H$^+$]-III							
#1	3.34	372	0.2267	-210.73	137	138	64.0
					137	139	20.8
#2	3.39	366	0.3378	75.43	136	139	64.4
					137	138	12.9
#5	3.78	328	0.2991	396.54	135	139	26.9
					134	138	21.5
					135	138	15.9
#7	3.95	314	0.1862	397.23	133	138	20.9
					132	138	17.6
					132	139	16.1
					133	139	14.5
					134	139	11.3
#8	3.97	312	0.1551	-576.92	133	138	28.3
					132	139	26.4
					135	139	13.1
					134	138	11.8

Table S2.5. Selected dominant excitations and occupied (occ) - unoccupied (unocc) MO-pair contributions (greater than 10 %) for dominant double-protonated structure of (+)-**2**: [**2**,2H$^+$]-I. For structure visualization, see Figure S2.3. For the corresponding simulated ECD spectrum, see Figure S2.15. For MOs isosurfaces, see Figure S2.20. LC-PBE0*/SV(P)/PCM(CH_3CN) calculations.

Excit. no.	E (eV)	λ (nm)	f	R (10^{-40} cgs)	occ no.	unocc no.	(%)
#1	3.52	352	0.4185	858.22	130	133	56.5
					132	134	26.7
#2	3.55	350	0.3331	-513.85	132	133	70.0
					130	134	17.4
#4	3.80	326	0.0094	-80.94	131	134	43.3
					130	134	23.5
					128	133	19.8
#5	3.85	322	0.0767	-94.49	129	133	46.8
					128	134	34.4
#6	3.89	319	0.0703	-66.97	129	134	34.2
					128	133	30.3
					130	134	13.8
					131	134	12.0

Table S2.6. Selected dominant excitations and occupied (occ) - unoccupied (unocc) MO-pair contributions (greater than 10 %) for dominant double-protonated structures of (+)-**3**: [**3**,2H$^+$]-I and [**3**,2H$^+$]-II. For structures visualization, see Figure S2.3. For the corresponding simulated ECD spectra, see Figure S2.15. For MOs isosurfaces, see Figures S2.21–S2.22. LC-PBE0*/SV(P)/PCM(CH_3CN) calculations.

Excit. no.	E (eV)	λ (nm)	f	R (10^{-40} cgs)	occ no.	unocc no.	(%)
[**3**,2H$^+$]-I							
#1	3.28	378	0.0965	-459.73	163	164	54.5
					162	165	37.3
#2	3.35	370	0.6928	394.54	163	165	46.0
					162	164	44.8
#3	3.47	357	0.0415	205.12	161	165	50.9
					163	165	26.6
					162	164	13.0
#6	3.68	337	0.0344	67.46	160	165	44.4
					162	165	19.6
					161	164	15.4
					163	164	10.1
#7	3.83	324	0.1070	210.90	159	165	42.7
					158	164	34.7
#8	3.87	321	0.0410	-104.54	159	164	46.3
					158	165	23.5
[**3**,2H$^+$]-II							
#1	3.29	377	0.2332	-989.16	162	164	45.9
					163	165	45.6
#2	3.32	373	0.4236	554.78	163	164	78.6
					162	165	11.7
#3	3.39	366	0.2054	569.45	161	164	47.5
					162	165	41.7
#4	3.44	360	0.0068	-79.91	161	165	37.5
					163	165	34.5
					162	164	21.2
#6	3.67	338	0.0131	-76.08	160	165	53.1
					161	164	16.7
					162	165	16.3
#7	3.83	323	0.1444	201.43	159	164	42.7
					158	165	36.5
#8	3.86	321	0.0348	-115.03	159	165	42.8
					158	164	27.0
#9	4.10	303	0.1494	68.44	161	164	15.2
					160	165	14.9
					162	165	11.1

Table S2.7. Selected dominant excitations and occupied (occ) - unoccupied (unocc) MO-pair contributions (greater than 10 %) for dominant double-protonated structure of (+)-**4**: [**4**,2H$^+$]-I. For structure visualization, see Figure S2.3. For the corresponding simulated ECD spectrum, see Figure S2.15. For MOs isosurfaces, see Figure S2.23. LC-PBE0*/SV(P)/PCM(CH_3CN) calculations.

Excit. no.	E (eV)	λ (nm)	f	R (10^{-40} cgs)	occ no.	unocc no.	(%)
#1	3.32	373	0.6042	743.83	158	159	57.5
					157	160	30.8
#2	3.33	372	0.0821	-439.87	157	159	48.4
					158	160	42.5
#4	3.72	333	0.1143	69.46	156	160	44.3
					155	159	28.2
					154	159	18.1
#5	3.76	330	0.1142	-42.48	155	159	42.3
					154	159	36.8
					157	160	10.0
#6	3.82	324	0.0126	-46.15	154	160	31.5
					157	159	27.7
					158	160	24.4
#9	4.14	300	0.1312	117.01	158	161	28.8
					157	162	20.4
					153	160	20.2
#10	4.16	298	0.1643	-163.74	153	159	27.6
					158	162	19.2
					157	161	18.8
#11	4.42	280	0.1298	-280.50	153	159	21.4
					154	160	13.3
					158	162	12.8
					157	161	12.6
					156	159	10.0

Table S2.8. Selected dominant excitations and occupied (occ) - unoccupied (unocc) MO-pair contributions (greater than 10 %) for dominant double-protonated structures of (+)-**5**: [**5**,2H$^+$]-II, [**5**,2H$^+$]-I′, and [**5**,2H$^+$]-II′. For structures visualization, see Figure S2.3. For the corresponding simulated ECD spectra, see Figure S2.15. For MOs isosurfaces, see Figures S2.24–S2.26. LC-PBE0*/SV(P)/PCM(CH_3CN) calculations.

Excit. no.	E (eV)	λ (nm)	f	R (10^{-40} cgs)	occ no.	unocc no.	(%)
[**5**,2H$^+$]-II							
#1	3.28	378	0.1114	34.56	163	164	90.5
#2	3.33	372	0.1811	-491.13	163	165	58.0
					162	164	34.6
#3	3.41	364	0.0414	-234.76	162	164	48.4
					163	165	34.7
					161	165	13.0
#4	3.45	360	0.4493	781.90	162	165	68.8
					161	164	23.6
#5	3.75	330	0.0837	348.03	161	164	45.4
					162	165	20.8
					160	165	19.4
#6	3.78	328	0.0672	-350.61	161	165	55.6
					160	164	21.7
#7	3.92	316	0.1050	87.26	159	164	44.3
					158	165	23.5
					160	165	19.2
[**5**,2H$^+$]-I′							
#1	3.29	377	0.0464	-16.24	163	164	71.7
					162	164	21.9
#2	3.42	362	0.1406	-54.72	162	164	62.3
					163	164	17.2
					161	164	15.3
#3	3.61	344	0.1852	86.33	161	164	51.9
					160	164	16.0
#6	3.81	325	0.3085	61.45	163	166	64.9
#11	4.26	291	0.1918	335.83	161	165	28.1
					163	165	12.1
					162	165	10.8
					160	166	10.0
#12	4.33	287	0.1233	135.39	163	167	20.9
					161	165	11.2
					160	165	10.3
#13	4.34	286	0.2084	224.00	160	165	18.0
					163	165	13.6
#14	4.35	285	0.1779	124.83	163	167	44.8
#15	4.38	283	0.1307	-458.07	158	165	17.8
					161	166	16.5
					160	166	12.2
[**5**,2H$^+$]-II′							
#1	3.24	383	0.0180	2.07	163	164	78.8
#2	3.38	367	0.1743	-114.08	162	164	73.0
					161	164	12.8
#3	3.58	346	0.1686	56.40	161	164	42.2
					162	165	19.5
					162	164	10.6
#4	3.64	341	0.1046	100.34	162	165	36.1
					161	164	16.7
					161	165	15.0
#9	4.08	304	0.0740	-48.99	158	165	29.4
					160	166	12.6
					161	166	10.8
					163	165	10.0
#11	4.18	296	0.0669	146.76	161	165	35.9

Table S2.9. Selected dominant excitations and occupied (occ) - unoccupied (unocc) MO-pair contributions (greater than 10 %) for dominant double-protonated structures of (+)-**6**: [**6**,2H$^+$]-I′ and [**6**,2H$^+$]-I″. For structures visualization, see Figure S2.3. For the corresponding simulated ECD spectra, see Figure S2.15. For MOs isosurfaces, see Figures S2.27–S2.28. LC-PBE0*/SV(P)/PCM(CH_3CN) calculations.

Excit. no.	E (eV)	λ (nm)	f	R (10^{-40} cgs)	occ no.	unocc no.	(%)
[**6**,2H$^+$]-I′							
#1	3.44	361	0.3293	385.98	157	159	63.7
					158	159	28.1
#2	3.62	343	0.0973	-140.13	156	159	48.4
					158	159	28.9
#5	3.90	318	0.1716	84.87	158	161	46.6
					155	159	28.9
#6	3.94	315	0.0888	-109.45	155	159	47.4
					158	161	21.6
[**6**,2H$^+$]-I″							
#1	3.61	344	0.0086	-13.09	158	159	53.9
					157	160	17.4
#2	3.61	343	0.0088	17.74	158	160	50.8
					157	159	18.4
#3	3.73	332	0.3052	651.98	158	161	75.6
#4	3.88	320	0.1725	-477.13	158	162	41.8
					157	161	28.4

Table S2.10. Selected dominant excitations and occupied (occ) - unoccupied (unocc) MO-pair contributions (greater than 10 %) for dominant neutral structure of (+)-**7**: **7**-I. For structure visualization, see Figure S2.4. For the corresponding simulated ECD spectrum, see Figure S2.16. For MOs isosurfaces, see Figure S2.29. LC-PBE0*/SV(P)/PCM(CH_3CN) calculations.

Excit. no.	E (eV)	λ (nm)	f	R (10^{-40} cgs)	occ no.	unocc no.	(%)
#1	3.60	345	0.1435	636.27	123	125	91.0
#2	3.66	338	0.3686	-357.18	124	125	90.1
#3	4.08	304	0.0653	93.44	121	125	46.3
					118	125	24.7
#4	4.10	303	0.0196	70.34	122	125	35.5
					119	125	23.2
					117	125	16.7
					118	126	10.2
#7	4.31	288	0.1944	-60.96	120	125	75.8
#8	4.39	283	0.0504	-177.67	123	126	42.5
					124	127	13.4
					117	125	12.1
					122	125	10.5
#9	4.58	271	0.0355	-77.82	124	126	64.1
					121	125	11.9

Table S2.11. Selected dominant excitations and occupied (occ) - unoccupied (unocc) MO-pair contributions (greater than 10 %) for dominant double-protonated structure of (+)-**7**: [**7**,2H$^+$]-I. For structure visualization, see Figure S2.4. For the corresponding simulated ECD spectrum, see Figure S2.16. For MOs isosurfaces, see Figure S2.8. LC-PBE0*/SV(P)/PCM(CH_3CN) calculations.

Excit. no.	E (eV)	λ (nm)	f	R (10^{-40} cgs)	occ no.	unocc no.	(%)
#1	3.08	403	0.1465	645.93	123	125	87.9
#2	3.15	394	0.4559	-334.65	124	125	93.9
#3	3.51	353	0.1801	119.69	122	125	63.2
					124	126	21.1
#6	3.94	315	0.2337	-314.80	123	126	84.0
#8	4.32	287	0.0215	-66.30	122	126	66.9

2.3. Cartesian coordinates for optimized structures

Optimized (BLYP/TZVP/COSMO(CH_3CN)) geometries of the considered species (mono- and double-protonated structures of **1**–**6**, neutral and protonated structures of **7**) along with the corresponding absolute energies: the atomic symbol followed by three Cartesian coordinates, in Å.

[**1**,1H$^+$]-I
Total energy: –1649.0737773047 au
C -2.1554648 -3.4374182 -1.2535853
C -0.8884186 -3.1641077 -0.6619826
C -0.3166988 -4.1310960 0.2372498
C -1.0310783 -5.3297928 0.5170205
C -2.2616038 -5.5652308 -0.0714886
C -2.8254794 -4.6127428 -0.9630429
N -0.2498607 -1.9944586 -0.9695740
C 0.9363040 -1.7297718 -0.4259622
C 1.5996220 -2.6482042 0.4682202
C 0.9578538 -3.8282385 0.7856488
C 1.6218993 -0.4479036 -0.7831295
C 3.0376877 -0.4027114 -0.6472924
C 3.7891383 -1.6068230 -0.1093080
C 2.9741543 -2.3021719 0.9948062
C 3.7281329 0.7541859 -1.0203325
C 3.0627577 1.8791800 -1.5112437
C 1.6633977 1.8576762 -1.6206209
C 0.9215777 0.7008094 -1.2506439
C -0.5759144 0.7828120 -1.4108825
C -1.0838950 0.6287775 -2.7245671
C -2.4572867 0.7324683 -2.9797168
C -3.3444236 0.9717191 -1.9264953
C -2.8954601 1.1251217 -0.6169691
C -1.4907594 1.0556328 -0.3534497
C -3.8799313 1.3954823 0.5038344
C -3.4303671 0.7017348 1.7987158
C -2.0150947 1.1108253 2.1277608
C -1.0905178 1.2797945 1.0478880
N 0.1657660 1.6787221 1.3638620
C 0.6410969 1.9136704 2.6388313
C -0.2608948 1.7348731 3.7289368
C -1.5892496 1.3340932 3.4249172
C 0.2159573 1.9721171 5.0462212

C 1.5264583 2.3678032 5.2515807
C 2.4060590 2.5422412 4.1528531
C 1.9756539 2.3194718 2.8539468
O -0.1540449 0.3534474 -3.6914190
C -0.5975027 0.2147184 -5.0614396
O 0.9201493 2.9312430 -2.0488001
C 1.6126666 4.1243856 -2.4895287
H 0.3097966 0.0253118 -5.6408781
H -1.0784272 1.1361639 -5.4169490
H -1.2888063 -0.6320988 -5.1704264
H -2.5758579 -2.6981214 -1.9329095
H -3.7941291 -4.8137503 -1.4188690
H -2.8036348 -6.4845698 0.1456642
H -0.5942071 -6.0591608 1.1995444
H 1.4337628 -4.5452217 1.4565793
H 2.8854939 -1.6231531 1.8595272
H 3.4825368 -3.2070611 1.3507139
H 4.7688375 -1.2927075 0.2713637
H 3.9798548 -2.3257438 -0.9238595
H 4.8127791 0.7838022 -0.9206736
H 3.6309133 2.7616104 -1.7898086
H 2.2762669 3.9033255 -3.3359568
H 2.1894029 4.5743722 -1.6701437
H 0.8251152 4.8124513 -2.8067109
H -3.9596145 2.4807943 0.6828551
H -4.8778416 1.0488275 0.2107731
H -4.0930514 0.9502052 2.6356296
H -3.4733720 -0.3912829 1.6632965
H -2.2931838 1.1989622 4.2459005
H -0.4652506 1.8378073 5.8846157
H 1.8894666 2.5484352 6.2612771
H 3.4330205 2.8547944 4.3306515
H 2.6510344 2.4555863 2.0107095
H -4.4115334 1.0357608 -2.1356963
H -2.8467395 0.6093023 -3.9860752
H 0.8249818 1.7930235 0.5890358

[**1**,1H$^+$]-II
Total energy: −1649.0820931282 au
C 2.9225573 -1.5033274 -1.5366939
C 2.1832697 -0.4122581 -2.0468585
C 2.6270818 0.2947671 -3.2084292
C 3.8435181 -0.1095787 -3.8219442
C 4.5668584 -1.1713393 -3.3071165
C 4.1013744 -1.8718886 -2.1654662
C 1.7978615 1.3326008 -3.7180712
C 0.6222054 1.6955671 -3.0880602
C 0.2622845 1.0286907 -1.8797590
N 1.0100838 -0.0066775 -1.4446920
C -0.3580082 2.7125692 -3.6256694
C -1.7981942 2.1925332 -3.4108833
C -2.0342295 1.8706685 -1.9454489
C -0.9594514 1.3854098 -1.1503119
C -3.2753207 2.0581402 -1.3355600

C -3.4539877 1.8110032 0.0286997
C -2.3621165 1.4323142 0.8305671
C -1.0815656 1.2306284 0.2562213
O -2.4438962 1.2778098 2.1915689
C -3.7262388 1.4630927 2.8368963
C 0.0955259 1.1347263 1.2010076
C 0.7785892 2.3510882 1.4740440
C 1.7396482 2.4192976 2.4959314
C 1.9925693 1.2971608 3.2891858
C 1.3450657 0.0824881 3.0536640
C 0.4322583 -0.0098773 1.9689868
C -0.1199138 -1.3554272 1.6707802
C -0.2568488 -2.3096163 2.7368343
C 0.2625495 -1.9272822 4.1048431
C 1.5731739 -1.1263479 3.9447897
C -0.7955190 -3.5441840 2.4385326
C -1.1795024 -3.8671094 1.1078632
C -0.9349376 -2.8823107 0.0891536
N -0.3991481 -1.6561233 0.3987992
C -1.7488763 -5.1177152 0.7399326
C -2.0575441 -5.3868673 -0.5823511
C -1.8047065 -4.4174660 -1.5883298
C -1.2526791 -3.1902283 -1.2625683
O 0.4123570 3.4349791 0.7109792
C 1.0588305 4.7068830 0.9532179
H 0.6020569 5.4015877 0.2434047
H 2.1394789 4.6438094 0.7667638
H 0.8775725 5.0547044 1.9793351
H 2.2590257 3.3494180 2.7066142
H 2.7036047 1.3781292 4.1111757
H -0.9169480 -4.2927120 3.2227555
H 0.4210529 -2.8260781 4.7133788
H -0.4783121 -1.3035811 4.6315437
H 2.3471778 -1.7847261 3.5157174
H 1.9445803 -0.7978072 4.9234569
H -1.9355408 -5.8602433 1.5156576
H -2.4943894 -6.3455758 -0.8572908
H -2.0505950 -4.6426469 -2.6250198
H -1.0660473 -2.4427162 -2.0316355
H 2.0972541 1.8372259 -4.6367429
H -0.1597008 2.8981013 -4.6875348
H -0.2348732 3.6708406 -3.0962631
H -1.9579136 1.2994563 -4.0372868
H -2.5252062 2.9441086 -3.7417473
H -4.1191498 2.4133397 -1.9260668
H -4.4332288 1.9688935 0.4710147
H 4.1902963 0.4258588 -4.7042664
H 5.4970728 -1.4780105 -3.7811002
H 4.6781033 -2.7085738 -1.7761509
H 2.5682191 -2.0388000 -0.6583371
H -3.5427903 1.2793418 3.8985784
H -4.4657144 0.7442888 2.4589297
H -4.0958673 2.4879979 2.6968223

H 0.5658036 -0.6224240 -0.6999915

[**1**,1H^{+}]-IIIa
Total energy: –1649.0739866668 au
C 1.9090649 -5.0990684 -1.1138394
C 1.9322408 -3.7665270 -0.6150001
C 0.7918399 -2.9146506 -0.8251396
C -0.3366615 -3.4379859 -1.5195927
C -0.3300808 -4.7379460 -1.9941154
C 0.8005065 -5.5746389 -1.7926022
C 3.0233103 -3.2190698 0.1109557
C 2.9846461 -1.9165206 0.5645339
C 1.8148517 -1.1255901 0.2666621
N 0.7597034 -1.6211181 -0.3781002
C 1.7807733 0.2986712 0.7180236
C 2.6057822 0.6581149 1.8184006
C 3.5018967 -0.3841810 2.4614144
C 4.1001716 -1.3054136 1.3828080
C 2.5974165 1.9764839 2.2834651
C 1.8172982 2.9623705 1.6763940
C 1.0281015 2.6272044 0.5650726
C 0.9788241 1.2884366 0.0816118
C 0.1946708 1.0684332 -1.1908548
C -1.2097029 0.8318057 -1.2729442
C -1.8538737 0.7846080 -2.5470486
C -1.0896789 0.9452459 -3.7021901
C 0.2897428 1.1484757 -3.6408083
C 0.9281172 1.2184150 -2.3935676
C -2.0702371 0.5759998 -0.1030254
C -3.3643096 -0.0252022 -0.2330307
C -3.8534766 -0.4084988 -1.6090674
C -3.3549798 0.6018541 -2.6494485
C -4.1214591 -0.2557644 0.9017771
C -3.6639720 0.0819477 2.2028303
C -2.3706859 0.6714462 2.3124458
N -1.6577558 0.8833608 1.1500182
C -1.8372478 1.0329042 3.5685525
C -2.5948137 0.8068924 4.7073685
C -3.8838948 0.2217561 4.6203287
C -4.4116076 -0.1353071 3.3914106
O 2.2752285 1.4360832 -2.2474836
C 3.0906084 1.5634961 -3.4360745
O 0.2594033 3.5375061 -0.1213497
C 0.2827709 4.9244706 0.2984103
H 3.0519864 0.6473379 -4.0408500
H 4.1085632 1.7211641 -3.0707782
H 2.7774520 2.4243237 -4.0423798
H 0.8546220 1.2611042 -4.5615011
H -1.5786702 0.9092153 -4.6748043
H -5.1037968 -0.7166212 0.7989998
H -0.8483769 1.4839453 3.6364386
H -2.1944389 1.0833149 5.6806798
H -4.4577206 0.0541006 5.5294933
H -5.3999352 -0.5856578 3.3168266

H -0.3839622 5.4445027 -0.3939081
H -0.0904867 5.0348827 1.3253465
H 1.2963009 5.3396349 0.2223488
H 1.8482448 3.9806285 2.0521485
H 3.2243187 2.2474434 3.1325460
H 3.8935610 -3.8440150 0.3175542
H -1.1958991 -2.7875077 -1.6711076
H -1.1962334 -5.1268032 -2.5279413
H 0.7894839 -6.5945889 -2.1740761
H 2.7769802 -5.7387476 -0.9523068
H -3.8582875 1.5692891 -2.4856944
H -3.6212780 0.2732044 -3.6606525
H -3.4713914 -1.4117349 -1.8599230
H -4.9477150 -0.4744172 -1.6097832
H 2.9250967 -0.9937354 3.1770147
H 4.2963130 0.1109357 3.0330955
H 4.7639929 -0.7130879 0.7309618
H 4.7141287 -2.0950405 1.8341746
H -0.7153848 1.2784301 1.2408770

[**1**,1H$^+$]-IIIb
Total energy: –1649.0731025216 au
C -4.7563199 -2.4178647 -1.7010614
C -3.4934812 -2.2875658 -1.0581449
C -2.4000514 -1.6809884 -1.7691999
C -2.6121634 -1.2253675 -3.1026532
C -3.8523836 -1.3640485 -3.7004393
C -4.9318483 -1.9638550 -2.9967487
N -1.1640738 -1.5332870 -1.2044815
C -0.9493633 -1.9504447 0.0412077
C -1.9853181 -2.5717836 0.8328918
C -3.2340368 -2.7292748 0.2658908
C -1.6790166 -3.0274506 2.2418291
C -0.2438937 -3.5691031 2.3198051
C 0.7457499 -2.5542505 1.7785043
C 0.4125614 -1.7636869 0.6400918
C 1.3665418 -0.8320093 0.1291133
C 2.6307897 -0.7166567 0.7693887
C 2.9445666 -1.5020749 1.8879043
C 2.0013747 -2.4092223 2.3739439
C 1.1690390 0.0092293 -1.1089756
C 0.7215857 1.3621626 -1.1167486
C 0.6353682 2.0809678 -2.3522979
C 1.0303707 1.4527838 -3.5298682
C 1.5205148 0.1430903 -3.5309181
C 1.5943420 -0.5719185 -2.3303606
C 0.1060039 3.5007172 -2.3832139
C 0.6108145 4.2904149 -1.1677550
C 0.2830005 3.5444645 0.1032307
C 0.3418192 2.1127150 0.0939418
N 0.0388836 1.4745709 1.2512428
C -0.2994520 2.0828655 2.4441765
C -0.3609450 3.5068185 2.4727193
C -0.0625846 4.2009158 1.2703798

C -0.5777447 1.3189341 3.5978917
C -0.9151942 1.9749159 4.7717360
C -0.9835365 3.3904775 4.8206431
C -0.7122982 4.1452578 3.6923978
O 2.0614880 -1.8570634 -2.2427174
C 2.5060998 -2.5167382 -3.4509995
O 3.4907356 0.2041826 0.2254755
C 4.8025450 0.3646907 0.8176269
H 4.7271738 0.6996989 1.8608943
H 5.2960437 1.1338006 0.2181278
H 5.3754328 -0.5708768 0.7659290
H 3.9080532 -1.4131945 2.3806848
H 2.2518414 -3.0172838 3.2427810
H -4.0357376 -3.1994227 0.8375564
H -1.7770095 -0.7678881 -3.6297127
H -4.0074081 -1.0119862 -4.7194380
H -5.9006338 -2.0646900 -3.4838337
H -5.5818364 -2.8785018 -1.1579794
H 1.6867379 -2.6074079 -4.1770179
H 3.3501201 -1.9814618 -3.9068285
H 2.8287220 -3.5118908 -3.1341813
H 1.8365099 -0.3068162 -4.4675036
H 0.9691237 1.9970719 -4.4714214
H -0.1046558 5.2899754 1.2738669
H -0.5237266 0.2321894 3.5632366
H -1.1301842 1.3945818 5.6666989
H -1.2509578 3.8815997 5.7540272
H -0.7613367 5.2323901 3.7219654
H -0.1786111 -4.5002101 1.7318211
H 0.0173689 -3.8285849 3.3530400
H -1.7842933 -2.1772563 2.9373982
H -2.4054470 -3.7856047 2.5605052
H -0.9967446 3.4913791 -2.3768682
H 0.4139146 3.9916366 -3.3136788
H 1.7035361 4.4173245 -1.2408778
H 0.1709626 5.2939260 -1.1327675
H 0.1025527 0.4519826 1.2481270

[**2**,1H$^+$]-I
Total energy: –1608.581548357 au
C -0.0826900 -2.0643044 3.5521199
C 0.3404608 -2.1542706 2.1945168
C 1.6148042 -2.7557756 1.9000737
C 2.4218024 -3.2346104 2.9699320
C 1.9855696 -3.1269815 4.2789447
C 0.7246002 -2.5396529 4.5701901
C 1.9829969 -2.8579264 0.5320250
C 1.1504617 -2.3751701 -0.4575696
C -0.0879886 -1.7543397 -0.0619585
N -0.4778806 -1.6709416 1.2101552
C 1.4527408 -2.5151779 -1.9323825
C 0.1607493 -2.8796045 -2.6866601
C -0.9378844 -1.8709425 -2.4001756
C -1.0140880 -1.2536619 -1.1228742

C -2.0057360 -0.2509897 -0.8874326
C -2.9835684 -0.0391350 -1.8840814
C -2.9316116 -0.6769698 -3.1252081
C -1.8873610 -1.5637883 -3.3860085
O -3.9974956 0.8948582 -1.6475241
C -4.9878451 0.4690430 -0.6959674
O -4.4064427 -0.1079012 0.4863064
C -3.3384321 0.6180016 1.0189457
C -3.5093511 1.2640040 2.2455701
C -2.4309651 1.9318549 2.8254413
C -1.2032145 2.0228947 2.1590823
C -1.0482490 1.3996663 0.8877989
C -2.1024979 0.5983213 0.3477078
C -0.0560256 2.8026577 2.7734624
C 1.3008063 2.1913811 2.3811655
C 1.3861821 2.0846578 0.8777833
C 0.2047246 1.6955361 0.1764720
C 2.5323976 2.3754584 0.1590848
C 2.5682405 2.3137936 -1.2596654
C 1.3644259 1.9610083 -1.9418356
N 0.2503172 1.6777754 -1.1771119
C 3.7227341 2.6035248 -2.0360795
C 3.6657887 2.5442612 -3.4176286
C 2.4575865 2.1965075 -4.0744596
C 1.3114650 1.9062593 -3.3507906
H -4.4770494 1.2181714 2.7417799
H -2.5492888 2.4089047 3.7970165
H -0.0957588 3.8516986 2.4369017
H -0.1664360 2.8175806 3.8640678
H 2.1317184 2.7963976 2.7605382
H 1.3944783 1.1900995 2.8308069
H 3.4371539 2.6673365 0.6919200
H 0.3823529 1.6423932 -3.8531367
H 2.4288832 2.1569215 -5.1614509
H 4.5511232 2.7670365 -4.0095177
H 4.6455618 2.8729552 -1.5255433
H -5.6258179 -0.3218269 -1.1092855
H -3.6942172 -0.4616282 -3.8710906
H -1.8198564 -2.0445061 -4.3612064
H -0.1683588 -3.8855182 -2.3767935
H 0.3437421 -2.9267851 -3.7672213
H 2.2281397 -3.2744311 -2.0916114
H 1.8478886 -1.5665038 -2.3324933
H 2.9272683 -3.3362725 0.2676205
H -1.0503789 -1.6116192 3.7619718
H 0.3947540 -2.4649603 5.6054791
H 2.6077453 -3.4949323 5.0934619
H 3.3862249 -3.6889842 2.7424151
H -5.5573048 1.3762924 -0.4592909
H -0.6120947 1.4528588 -1.6767023

[**3**,1H^{+}]-I
Total energy: –1956.3428058666 au
C -2.3716784 5.7998845 1.9884379

C -1.4839785 4.7426434 2.3154509
C -1.7156489 3.4388310 1.7543688
C -2.8312101 3.2543682 0.8978287
C -3.6824892 4.3078459 0.5994960
C -3.4519593 5.5901118 1.1456403
C -0.7900500 2.3886417 2.1106819
C 0.2895305 2.6300879 3.0016426
C 0.4806235 3.9464872 3.5346040
C -0.3722205 4.9612442 3.1980932
C 1.1243922 1.5412405 3.3481340
C 0.9305449 0.2673062 2.8397046
C -0.1247246 0.0496455 1.9096885
N -0.9225423 1.1119876 1.6113094
C -0.3682763 -1.2870609 1.3311283
C 0.0960028 -2.3865302 2.1198298
C 0.8589101 -2.1439392 3.4077422
C 1.7696329 -0.9138956 3.2602322
C -1.0122537 -1.5197814 0.0838333
C -1.2230836 -2.8623065 -0.3267872
C -0.8308511 -3.9337750 0.4861817
C -0.1693731 -3.6848047 1.6912696
C -1.5457968 -0.4473989 -0.8298629
C -0.7439711 0.3868440 -1.6592601
C -1.3675052 1.3693627 -2.4815162
C -2.7612439 1.4409199 -2.5335361
C -3.5664568 0.6062375 -1.7560307
C -2.9636220 -0.3137288 -0.8855480
C -0.5167888 2.3347126 -3.2864596
C 0.7337664 2.7291981 -2.4805400
C 1.4846439 1.4736256 -2.0995731
C 0.7499732 0.2962996 -1.7355366
C 2.8652903 1.3908046 -2.1389486
C 3.5245867 0.1687209 -1.8729962
C 2.7072047 -0.9714982 -1.5889379
N 1.3526284 -0.8784999 -1.5107978
C 4.9538437 0.0400890 -1.9175578
C 5.5551077 -1.1718382 -1.7113488
C 4.7783666 -2.3554624 -1.4561040
C 3.3474280 -2.2688834 -1.3941995
C 5.4032013 -3.6177674 -1.2710314
C 4.6496851 -4.7596111 -1.0401219
C 3.2398663 -4.6731923 -0.9844140
C 2.6040239 -3.4504678 -1.1566433
O -3.6751902 -1.1175773 -0.0278896
C -5.1211036 -1.0807060 -0.0789925
O -1.8014512 -3.0242580 -1.5585120
C -2.1211052 -4.3613626 -2.0084218
H -2.5965295 -4.2310131 -2.9839221
H -2.8187407 -4.8545560 -1.3181249
H -1.2142789 -4.9713867 -2.1199398
H 6.6407314 -1.2614802 -1.7480986
H 5.5509849 0.9280965 -2.1251594
H 3.4568939 2.2695075 -2.4001033
H 0.4241531 3.2819037 -1.5777056

H 1.3839078 3.3985286 -3.0575558
H -1.1084714 3.2207640 -3.5472781
H -0.2013815 1.8698262 -4.2356171
H -3.2340538 2.1769868 -3.1831142
H -4.6470982 0.6935454 -1.8153917
H -5.4536130 -1.8095745 0.6644397
H -5.4877307 -1.3696817 -1.0729554
H -5.5037090 -0.0851282 0.1842939
H 0.1596673 -1.9787386 4.2442778
H 1.4505708 -3.0319269 3.6588415
H 2.2905140 -0.6854791 4.1969802
H 2.5399501 -1.1151679 2.4984757
H 1.9398144 1.7150815 4.0497976
H 1.3154464 4.1157024 4.2120195
H -0.2241401 5.9608442 3.6041265
H 0.1609599 -4.5257861 2.2995093
H -1.0069641 -4.9598902 0.1770609
H -2.1899236 6.7849771 2.4159002
H -4.1255237 6.4105218 0.9046431
H -4.5337412 4.1450025 -0.0586525
H -3.0468472 2.2826785 0.4590161
H 6.4906613 -3.6763560 -1.3174799
H 5.1418985 -5.7215462 -0.9031374
H 2.6511206 -5.5716458 -0.8037322
H 1.5217773 -3.3789171 -1.1081121
H -1.6909838 0.9315130 0.9629677

[**3**,1H$^+$]-II
Total energy: −1956.3474337923 au
C -2.5164896 -0.3168141 -1.9318234
C -2.1594229 -1.6798700 -2.0780174
C -3.0523705 -2.5443870 -2.7973487
C -4.2651230 -2.0147470 -3.3137849
C -4.5936602 -0.6787466 -3.1398735
C -3.7077533 0.1766776 -2.4470777
C -2.7080483 -3.9261161 -2.9937817
C -1.5378375 -4.4390087 -2.5057502
C -0.6220661 -3.6137124 -1.7706436
C -0.9191856 -2.2318826 -1.5428733
C 0.5700553 -4.1339987 -1.2134157
C 1.4315024 -3.3205431 -0.5010555
C 1.0824655 -1.9405204 -0.3603817
N -0.0718154 -1.4351185 -0.8278378
C 2.6751973 -3.8317902 0.1898801
C 2.7980468 -3.1598249 1.5723772
C 2.7874543 -1.6488808 1.4221939
C 1.9927929 -1.0479904 0.4085040
C 3.5664908 -0.8333790 2.2459215
C 3.5971320 0.5531667 2.0779972
C 2.8711419 1.1436707 1.0317325
C 2.0658789 0.3492133 0.1702653
C 1.5033952 1.0282672 -1.0528115
C 0.4805613 2.0123395 -1.0064192
C 0.2175921 2.8623938 -2.1185652

C 0.9064982 2.6311113 -3.3091008
C 1.8615378 1.6152494 -3.4070977
C 2.1839609 0.8364415 -2.2826123
C -0.3709635 2.2203112 0.1718436
C -0.9395758 3.4902587 0.4504917
C -0.7136902 4.5940656 -0.5554861
C -0.7743952 4.0026915 -1.9796675
C -1.7031914 3.6343950 1.5969565
C -1.9575726 2.5491440 2.4714685
C -1.4536964 1.2638074 2.1254665
N -0.6885001 1.1735218 0.9824009
C -1.7439399 0.1055789 2.9393832
C -2.5316556 0.3087976 4.1254577
C -3.0088698 1.6213715 4.4589270
C -2.7392373 2.7000709 3.6631675
C -2.8296455 -0.8038343 4.9538285
C -2.3783854 -2.0743586 4.6327253
C -1.6105076 -2.2752007 3.4642116
C -1.2987251 -1.2071340 2.6358441
O 3.1769440 -0.1100481 -2.2819841
C 3.9212721 -0.3392108 -3.5021357
O 2.9115153 2.4897892 0.7532392
C 3.7304755 3.3463733 1.5840672
H 4.7883289 3.0560082 1.5289709
H 3.6024235 4.3517544 1.1749070
H 3.3926259 3.3257943 2.6289609
H 4.2149475 1.1576990 2.7352348
H 4.1705222 -1.2853156 3.0325451
H 3.7216715 -3.4745715 2.0738283
H 1.9625230 -3.4927008 2.2111312
H 2.6342359 -4.9241538 0.2827098
H 3.5684637 -3.5892865 -0.4089581
H 0.7955882 -5.1939146 -1.3383677
H -1.2832279 -5.4873525 -2.6599289
H -3.4010941 -4.5607174 -3.5455838
H 4.4509856 0.5694091 -3.8189458
H 3.2620906 -0.6874088 -4.3086826
H 4.6449650 -1.1199944 -3.2547789
H 2.3836734 1.4680734 -4.3479215
H 0.7093045 3.2605766 -4.1760349
H -1.8007071 3.6556407 -2.1851756
H -0.5505597 4.7773001 -2.7226082
H 0.2745565 5.0526730 -0.3922388
H -1.4650698 5.3802476 -0.4196219
H -2.1339367 4.6063466 1.8363859
H -3.1134223 3.6906391 3.9147854
H -3.6017726 1.7409108 5.3645954
H -4.9370159 -2.6800666 -3.8557660
H -5.5271235 -0.2868203 -3.5409839
H -3.9611159 1.2284510 -2.3215462
H -1.8295856 0.3481965 -1.4206390
H -3.4248175 -0.6413026 5.8513954
H -2.6155243 -2.9187717 5.2773841
H -1.2626129 -3.2750966 3.2118608

H -0.7116164 -1.3997310 1.7419890
H -0.3932902 0.2458941 0.6422920

[**3**,1H$^+$]-IIIa
Total energy: –1956.3406574026 au
C 1.7934089 -1.2012045 3.0752132
C 0.6184375 -0.8613548 3.7932880
C 0.6279845 -0.9820703 5.2266936
C 1.8101023 -1.4267838 5.8726631
C 2.9445704 -1.7485262 5.1439742
C 2.9328055 -1.6362943 3.7362175
C -0.5475739 -0.6616067 5.9870101
C -1.6984163 -0.2467286 5.3762102
C -1.7543492 -0.1111529 3.9511283
C -0.6037505 -0.4025912 3.1728773
N -0.7019621 -0.2278406 1.8108110
C -1.8072718 0.1806448 1.1234112
C -2.9845698 0.4221001 1.8930190
C -2.9267755 0.2888572 3.2708690
C -4.2817231 0.7685100 1.2026897
C -4.0243920 1.5444720 -0.0890379
C -2.9682031 0.8711423 -0.9418288
C -1.7963173 0.3018947 -0.3504803
C -3.0990085 0.8805191 -2.3289366
C -2.1001696 0.3716928 -3.1596018
C -0.9101004 -0.1095095 -2.5967362
C -0.7271982 -0.1423064 -1.1885417
C 0.6796160 -0.4489091 -0.7338681
C 1.3010229 -1.7277176 -0.8288701
C 2.7061296 -1.8456203 -0.6407710
C 3.4581692 -0.7098172 -0.3285908
C 2.8678422 0.5489357 -0.2059804
C 1.4894382 0.6863550 -0.4258482
C 3.3820387 -3.1918308 -0.8204673
C 2.7488640 -3.9368674 -2.0080858
C 1.2583076 -4.0528504 -1.7844630
C 0.5473913 -2.9751264 -1.1597528
N -0.7533954 -3.0595662 -0.8498436
C -1.4527510 -4.1821629 -1.1711727
C -0.8370043 -5.2840723 -1.8463861
C 0.5440702 -5.1854189 -2.1302224
C -2.8643459 -4.2776787 -0.8115712
C -3.5956205 -5.4613483 -1.1639595
C -2.9322407 -6.5310848 -1.8596476
C -1.6059004 -6.4481865 -2.1846633
C -4.9688958 -5.5570010 -0.8135268
C -5.6056403 -4.5285683 -0.1348105
C -4.8840463 -3.3643382 0.2134220
C -3.5416324 -3.2432233 -0.1215115
O 0.8373213 1.8936761 -0.3818571
C 1.5985783 3.0883074 -0.0757247
O 0.1505789 -0.5452724 -3.3505956
C 0.0293744 -0.5379829 -4.7924979
H -0.7894435 -1.1903937 -5.1243845

H 0.9820201 -0.9252395 -5.1625501
H -0.1281692 0.4808943 -5.1716252
H -2.2399500 0.3883813 -4.2363368
H -3.9978955 1.3028411 -2.7760432
H -3.8232810 0.4880519 3.8576768
H -0.5054941 -0.7630267 7.0705076
H -2.5904202 -0.0148491 5.9551319
H 2.0547983 3.0223616 0.9209779
H 2.3750829 3.2642441 -0.8317717
H 0.8709851 3.9033869 -0.0957865
H 3.4840362 1.4119192 0.0270227
H 4.5340456 -0.8050470 -0.1846801
H 1.0493192 -6.0226098 -2.6140089
H -3.5093834 -7.4183241 -2.1195059
H -1.1086801 -7.2676217 -2.7037536
H -3.6836661 2.5630630 0.1622560
H -4.9525778 1.6551356 -0.6613832
H -4.8161636 -0.1674248 0.9705147
H -4.9254426 1.3345220 1.8863473
H 3.2699541 -3.8042470 0.0899320
H 4.4578768 -3.0483691 -0.9784046
H 2.9502509 -3.3764391 -2.9362515
H 3.1907511 -4.9335906 -2.1317132
H -5.5176269 -6.4583535 -1.0864226
H -6.6583759 -4.6160115 0.1300245
H -5.3888593 -2.5605170 0.7474833
H -2.9827834 -2.3488492 0.1363829
H 1.8100843 -1.5142392 6.9583204
H 3.8439216 -2.0900233 5.6533116
H 3.8232012 -1.8928243 3.1655206
H 1.8269287 -1.1333806 1.9910114
H 0.1236262 -0.4344866 1.2372227

[**3**,1H$^+$]-IIIb
Total energy: –1956.3382894643 au
C 2.8111688 1.1649291 3.0128981
C 1.6316046 0.9000380 3.7481601
C 1.7488861 0.5821389 5.1431685
C 3.0386817 0.5349292 5.7364823
C 4.1781788 0.7971123 4.9897090
C 4.0604668 1.1160418 3.6180535
C 0.5665639 0.3265576 5.9210589
C -0.6782776 0.4000230 5.3589228
C -0.8387849 0.7230637 3.9694049
C 0.3043853 0.9544442 3.1402682
N 0.1808502 1.2342586 1.8135551
C -1.0342999 1.3369953 1.2566109
C -2.2352969 1.1811472 2.0314279
C -2.1097566 0.8563957 3.3700032
C -3.5914200 1.3953700 1.3991207
C -3.4915800 2.5111721 0.3505968
C -2.4029360 2.1845309 -0.6536810
C -1.1681800 1.6171007 -0.2153904
C -2.6074132 2.4454670 -2.0102419

C -1.6429462 2.1415441 -2.9717756
C -0.4244288 1.5864549 -2.5616736
C -0.1600502 1.3268240 -1.1850962
C 1.2667402 0.9431850 -0.8711977
C 1.8143603 -0.3723529 -0.8746650
C 3.2125328 -0.5530955 -0.6090519
C 4.0327378 0.5620358 -0.4715302
C 3.5222277 1.8601305 -0.5465179
C 2.1470693 2.0495649 -0.7202302
C 3.8014154 -1.9382119 -0.4441052
C 3.2179490 -2.8999675 -1.4803755
C 1.7107821 -2.8388422 -1.4596409
C 1.0528460 -1.6020898 -1.1867375
N -0.3105125 -1.6032353 -1.2629152
C -1.1083301 -2.6848408 -1.5729828
C -0.4631321 -3.9319070 -1.7787943
C 0.9481996 -3.9636491 -1.7236233
C -2.5439787 -2.5664240 -1.6910548
C -3.2895375 -3.7669683 -1.9614621
C -2.6081261 -5.0200650 -2.1250655
C -1.2456309 -5.1009456 -2.0499983
C -4.7009721 -3.6898661 -2.0821271
C -5.3635967 -2.4786498 -1.9602381
C -4.6297539 -1.2974001 -1.7148546
C -3.2496979 -1.3418663 -1.5808005
O 1.5593114 3.2864701 -0.7702318
C 2.4005897 4.4594170 -0.6787503
O 0.5896826 1.2672274 -3.4283576
C 0.4109329 1.5401494 -4.8392942
H -0.4312722 0.9670845 -5.2497597
H 1.3415725 1.2190908 -5.3139273
H 0.2543571 2.6126622 -5.0157486
H -1.8425965 2.3449513 -4.0194379
H -3.5517107 2.8852586 -2.3297844
H -3.0032289 0.7183551 3.9806701
H 0.6816601 0.0818940 6.9769039
H -1.5708564 0.2195929 5.9579550
H 2.9286515 4.4960324 0.2838756
H 3.1262663 4.4910540 -1.5028469
H 1.7181061 5.3096910 -0.7556356
H 4.1954418 2.7069174 -0.4516159
H 5.0982157 0.4196229 -0.2967011
H 1.4523443 -4.9117604 -1.9093326
H -3.2027147 -5.9098478 -2.3269008
H -0.7294972 -6.0481562 -2.1946984
H -3.2616266 3.4625130 0.8591418
H -4.4480840 2.6511127 -0.1674180
H -3.9352220 0.4650099 0.9178088
H -4.3302655 1.6420970 2.1720160
H 3.5778892 -2.3153149 0.5680158
H 4.8929097 -1.8920902 -0.5319134
H 3.5685067 -2.6159169 -2.4866227
H 3.5496560 -3.9299494 -1.3037728
H -5.2577525 -4.6043829 -2.2821810

H -6.4465186 -2.4337050 -2.0605879
H -5.1505165 -0.3451489 -1.6363356
H -2.7224541 -0.4077128 -1.4058933
H 3.1190997 0.2919657 6.7960372
H 5.1606119 0.7598105 5.4585009
H 4.9555080 1.3250490 3.0334153
H 2.7196106 1.4090506 1.9603894
H -0.7656148 -0.6991714 -1.1005955

[**4**,1H$^+$]-I
Total energy: –1915.8519900121 au
C -1.3323043 -1.6032170 4.0013033
C -0.0519846 -2.0856520 3.6346187
C 0.8297542 -2.5569641 4.6635225
C 0.3957792 -2.5195725 6.0155337
C -0.8639159 -2.0413173 6.3467687
C -1.7332039 -1.5821281 5.3309591
C 2.1298460 -3.0637238 4.3123061
C 2.5425186 -3.1208343 3.0084783
C 1.6894569 -2.6661019 1.9470411
C 0.3964173 -2.1279023 2.2463771
C 2.0566174 -2.7591383 0.5841522
C 1.1986597 -2.3220426 -0.4103839
C -0.0471040 -1.7386700 -0.0120964
N -0.4291948 -1.6689001 1.2699833
C 1.4873914 -2.4935785 -1.8842475
C 0.1942414 -2.9266500 -2.5996805
C -0.9261371 -1.9375870 -2.3289359
C -0.9948293 -1.2756720 -1.0722670
C -1.8958901 -1.6870801 -3.3095921
C -2.9450904 -0.7994412 -3.0711104
C -2.9806628 -0.1038712 -1.8614971
C -1.9912192 -0.2710185 -0.8648336
O -3.9883262 0.8464488 -1.6643567
C -4.9788398 0.4722415 -0.6930735
O -4.3938049 -0.0515155 0.5104110
C -3.3190441 0.6929191 1.0032654
C -3.4881754 1.4060541 2.1919326
C -2.4000599 2.0757528 2.7509501
C -1.1628680 2.1004355 2.0976110
C -1.0144256 1.4260403 0.8516763
C -2.0803087 0.6251894 0.3363964
C 0.2521884 1.6765217 0.1433775
C 1.4342709 2.0336597 0.8443527
C 1.3424401 2.1754432 2.3440273
C 0.0073565 2.8543693 2.7020950
C 2.5921627 2.2749786 0.1210459
C 2.6247385 2.2162959 -1.2936364
C 1.4157131 1.9215720 -1.9813466
N 0.3043583 1.6586435 -1.2158165
C 3.8137881 2.4746204 -2.0506620
C 3.7832809 2.4535733 -3.4181450
C 2.5693233 2.1855702 -4.1380282
C 1.3516635 1.9138826 -3.4236033

C 0.1651259 1.6678923 -4.1617995
C 0.1753053 1.6873267 -5.5486658
C 1.3725610 1.9494056 -6.2516143
C 2.5467759 2.1930458 -5.5562690
H -3.7193330 -0.6218111 -3.8149166
H -1.8363893 -2.2069782 -4.2648894
H -0.0973785 -3.9293511 -2.2450917
H 0.3576763 -3.0091017 -3.6812318
H 2.2859050 -3.2306315 -2.0329933
H 1.8424218 -1.5452511 -2.3206927
H 3.0181163 -3.2017822 0.3202297
H 2.7832359 -3.4131025 5.1116837
H 3.5249051 -3.5186703 2.7541336
H -5.5420911 1.3930156 -0.4967800
H -4.4607778 1.4041966 2.6803108
H -2.5151841 2.5982905 3.6991712
H 0.0155123 3.8938292 2.3349398
H -0.1149359 2.9054495 3.7903099
H 2.1942305 2.7536532 2.7187981
H 1.3873669 1.1822724 2.8180558
H 3.5081624 2.5334504 0.6516534
H 4.6887719 2.6508574 -3.9901816
H 4.7349064 2.6909844 -1.5130718
H -5.6236483 -0.3327828 -1.0668735
H 3.4723754 2.3987938 -6.0921395
H 1.3690642 1.9625276 -7.3400172
H -0.7458178 1.5026063 -6.0976253
H -0.7806555 1.4709571 -3.6600994
H 1.0703050 -2.8776401 6.7933195
H -1.1846949 -2.0204447 7.3874344
H -2.7224608 -1.2098310 5.5941175
H -1.9974263 -1.2491726 3.2186523
H -0.5665081 1.4463629 -1.7027300

[**5**,1H^{+}]-I
Total energy: –1988.4570331434 au
N 2.5119101 1.5827449 -3.1093740
C 1.4736884 1.1031025 -3.8428542
C 1.1615194 1.5501756 -5.1633755
C 1.9921576 2.5625911 -5.7074128
C 3.0448304 3.0539468 -4.9539822
C 3.2692714 2.5309548 -3.6576519
C 0.6457746 0.0798399 -3.2595841
C -0.4236921 -0.4982174 -3.9805963
C -0.7050007 -0.0229562 -5.3051403
C 0.0551314 0.9679023 -5.8708874
N 0.9052681 -0.3538108 -1.9857150
C 0.2210519 -1.3334890 -1.3408283
C -0.8273964 -1.9792015 -2.0689386
C -1.1397167 -1.5452249 -3.3470560
C 0.5644231 -1.7513609 0.0296410
C 0.2361478 -3.1050874 0.3560032
C -0.5017499 -3.9797579 -0.6387849
C -1.5321310 -3.1424398 -1.4153989

C 0.6072377 -3.6067961 1.6005602
C 1.2421294 -2.8026596 2.5489322
C 1.4980042 -1.4556374 2.2635300
C 1.1748153 -0.9049659 0.9981344
C 1.5283516 0.5421262 0.7754354
C 0.5828608 1.6043211 0.7719891
C 1.0157027 2.9382251 0.5308325
C 2.3804344 3.2017106 0.3941241
C 3.3286559 2.1783672 0.4365614
C 2.9040793 0.8514124 0.5946072
C -0.0077696 4.0541448 0.4197590
C -1.2731932 3.5425987 -0.2948102
C -1.8042906 2.3347593 0.4436890
C -0.8777509 1.4039857 1.0226590
N -1.2723827 0.3953771 1.8072447
C -2.5956769 0.1926433 2.0294228
C -3.5982261 1.0052237 1.4083716
C -3.1547190 2.0994203 0.6272863
C -4.9918723 0.7240108 1.6076945
C -5.3907639 -0.3128002 2.4063697
C -4.4258527 -1.1293248 3.0899232
C -3.0214688 -0.8803348 2.9296639
N -2.0807231 -1.6017580 3.5979135
C -2.4895628 -2.5825681 4.4022723
C -3.8455783 -2.9236257 4.6077701
C -4.8152117 -2.1865758 3.9500080
O 3.7593519 -0.2258591 0.5598475
C 5.1786929 0.0208228 0.4350306
O 2.0426790 -0.5850150 3.1701068
C 2.4353500 -1.0894323 4.4661985
H 5.6465721 -0.9668968 0.4557189
H 5.5498437 0.6245011 1.2742742
H 5.4137589 0.5206377 -0.5146320
H -0.1662519 1.3245262 -6.8758167
H -1.5343657 -0.4684461 -5.8511060
H -1.9515572 -2.0287628 -3.8896059
H -2.3003568 -2.7694708 -0.7191355
H -2.0455153 -3.7413713 -2.1763089
H -0.9978077 -4.8018053 -0.1096089
H 0.2038567 -4.4383658 -1.3514924
H 0.3791138 -4.6426995 1.8484989
H 1.4952145 -3.2220841 3.5179769
H 2.8508878 -0.2291204 4.9975190
H 3.2018284 -1.8710231 4.3722830
H 1.5701608 -1.4813527 5.0179949
H -0.2858323 4.4234350 1.4213404
H 0.4267493 4.9032097 -0.1222453
H -2.0428839 4.3229931 -0.3470183
H -1.0177467 3.2728535 -1.3332415
H -3.8921853 2.7697018 0.1833808
H -5.7275156 1.3546190 1.1084618
H -6.4496321 -0.5276327 2.5496991
H 2.7136581 4.2267207 0.2322978
H 4.3807483 2.4156109 0.3109810

H -5.8747486 -2.4022789 4.0872176
H -4.1093212 -3.7409876 5.2765538
H -1.7070826 -3.1376038 4.9247310
H 1.7952617 2.9403402 -6.7098028
H 3.7006861 3.8300095 -5.3425304
H 4.0980736 2.9035241 -3.0545920
H 1.6915994 0.1157113 -1.5209839

[**5**,1H$^+$]-II
Total energy: −1988.4650719166 au
N 2.4987163 1.6342056 0.1801353
C 2.2914814 1.9007113 1.4962512
C 3.1990221 2.6716836 2.2905284
C 4.3642630 3.1608105 1.6477554
C 4.5706893 2.8798697 0.3083807
C 3.6059528 2.1111687 -0.3848164
C 2.9100497 2.9234448 3.6739448
C 1.7721841 2.4320971 4.2558565
C 0.8355285 1.6523970 3.4981441
C 1.0933486 1.3955550 2.1260333
N 0.1886874 0.6548745 1.4098925
C -0.9668304 0.1745574 1.9265454
C -1.2191815 0.3389586 3.3186359
C -0.3267322 1.0792034 4.0740888
C -2.4215080 -0.3685289 3.8982107
C -2.5668751 -1.7506945 3.2260335
C -2.6545797 -1.5905084 1.7198768
C -1.9095128 -0.5627531 1.0756158
C -3.4726569 -2.4091275 0.9419914
C -3.5946201 -2.2084195 -0.4361024
C -2.9321298 -1.1343845 -1.0536498
C -2.0830963 -0.2794389 -0.3036258
C -1.6094455 0.9821599 -0.9837819
C -0.6025081 1.0049676 -1.9799372
C -0.4274937 2.1385612 -2.8149868
C -1.1894371 3.2819374 -2.5652475
C -2.1282388 3.3163655 -1.5292694
C -2.3590327 2.1662138 -0.7566523
C 0.3308399 -0.1309277 -2.1746104
C 0.9039553 -0.3481439 -3.4665366
C 0.5984257 0.6473155 -4.5632302
C 0.5632049 2.0711588 -3.9646878
N 0.6626387 -0.8620146 -1.1034317
C 1.5129669 -1.9133771 -1.2415591
C 2.0644770 -2.2625786 -2.5153563
C 1.7508474 -1.4301804 -3.6192434
C 1.8893827 -2.7006395 -0.0702194
C 2.7666388 -3.8223477 -0.2456824
C 3.2715920 -4.1503485 -1.5498408
C 2.9376589 -3.3951869 -2.6408947
C 3.1145981 -4.5743590 0.9043462
C 2.6107832 -4.2054942 2.1395097
C 1.7594082 -3.0798804 2.2084374
N 1.4036095 -2.3484338 1.1523468

O -3.3237899 2.0951120 0.2230501
C -4.1051723 3.2777332 0.5106237
O -3.0826754 -0.8223490 -2.3831006
C -3.9466454 -1.6495353 -3.1962692
H -4.6872349 3.5929451 -0.3662800
H -4.7834687 2.9870377 1.3170514
H -3.4637665 4.1041211 0.8464829
H -2.7030174 4.2222154 -1.3604830
H -1.0571847 4.1653211 -3.1900953
H 0.2873878 2.8029227 -4.7345041
H 1.5752098 2.3409223 -3.6186531
H 1.3465665 0.5730809 -5.3623556
H -0.3824287 0.4231492 -5.0140463
H 2.2016062 -1.6431518 -4.5895254
H 3.3341447 -3.6394448 -3.6261806
H 3.9353206 -5.0087598 -1.6509889
H -4.9804712 -1.6280573 -2.8249999
H -3.5854076 -2.6864121 -3.2315478
H -3.9061836 -1.2119080 -4.1970854
H -4.2439337 -2.8615607 -1.0117705
H -4.0387767 -3.2110640 1.4148088
H -1.7051447 -2.3802585 3.5023221
H -3.4640478 -2.2612569 3.5960736
H -3.3319512 0.2237040 3.7131169
H -2.3091764 -0.4639661 4.9844587
H -0.5083028 1.2204704 5.1393233
H 1.5586288 2.6190712 5.3067255
H 3.6168535 3.5137601 4.2556268
H 3.7784210 -5.4325887 0.8017765
H 2.8592632 -4.7587926 3.0432398
H 1.3550378 -2.7670708 3.1735974
H 5.0821401 3.7519603 2.2149811
H 5.4540976 3.2401801 -0.2147381
H 3.7505669 1.8833367 -1.4420868
H 0.4664634 0.2986837 0.4622533

[**5**,1H^{+}]-IIIa
Total energy: –1988.4564032149 au
N 0.7951859 -1.4546317 3.1952522
C 0.3863541 -2.6393492 2.6710915
C 0.7801127 -3.9118272 3.1894435
C 1.6450401 -3.9047949 4.3129243
C 2.0581172 -2.6945930 4.8444809
C 1.6066479 -1.4917473 4.2505946
C 0.2976768 -5.1156046 2.5716002
C -0.5389851 -5.0671947 1.4869439
C -0.9566114 -3.8086582 0.9383094
C -0.4954463 -2.6127838 1.5319828
N -0.9056940 -1.4110815 1.0186720
C -1.7313151 -1.2499725 -0.0467246
C -2.1916313 -2.4466246 -0.6861549
C -1.8099181 -3.6801299 -0.1846776
C -3.0507677 -2.3465075 -1.9226217
C -3.9405587 -1.1036182 -1.8682314

C -3.1290672 0.1317752 -1.5364077
C -2.0997626 0.0890287 -0.5451799
C -3.4312714 1.3415470 -2.1577997
C -2.7627510 2.5193559 -1.8221848
C -1.8043498 2.5039219 -0.7990903
C -1.4671505 1.2977963 -0.1282095
C -0.5900475 1.4755825 1.0878472
C 0.7993554 1.7648297 1.0276665
C 1.4793675 2.2496041 2.1750064
C 0.7845920 2.3711974 3.3808800
C -0.5685322 2.0420143 3.4789466
C -1.2608780 1.6174258 2.3352727
C 2.9321966 2.6721404 2.0560984
C 3.1598114 3.3838590 0.7067280
C 2.7269810 2.4688489 -0.4170877
C 1.5938236 1.6114693 -0.2229671
N 1.2472296 0.6611422 -1.0991782
C 1.9460535 0.5339172 -2.2572422
C 3.0097380 1.4316597 -2.5970917
C 3.3944829 2.3860305 -1.6244986
C 1.6307086 -0.5562408 -3.1821463
C 2.3349709 -0.6254567 -4.4319290
C 3.3441607 0.3450159 -4.7535775
C 3.6800567 1.3261639 -3.8619705
C 2.0188055 -1.6888037 -5.3137588
C 1.0739862 -2.6279012 -4.9394160
C 0.4538276 -2.4853529 -3.6785132
N 0.7050187 -1.4905391 -2.8273199
O -2.6085744 1.3390733 2.3263267
C -3.3518474 1.4668009 3.5616878
O -1.1577519 3.6355423 -0.3647493
C -1.4540697 4.8954423 -1.0097377
H -1.2010884 4.8636065 -2.0782367
H -0.8242741 5.6320874 -0.5042874
H -2.5116678 5.1661800 -0.8857744
H -3.0153282 3.4427195 -2.3349352
H -4.2086733 1.3698978 -2.9202945
H -2.1709384 -4.5848159 -0.6733360
H 0.6108288 -6.0742124 2.9829760
H -0.9003474 -5.9823052 1.0213898
H -2.9636415 0.7851636 4.3304732
H -3.3260951 2.5002173 3.9330392
H -4.3789533 1.1908642 3.3093068
H -1.0802543 2.1516919 4.4303738
H 1.3053298 2.7396031 4.2647592
H 4.2415021 3.0421370 -1.8299992
H 3.8528749 0.2686460 -5.7143487
H 4.4676467 2.0425697 -4.0953550
H -4.7185322 -1.2451140 -1.0992050
H -4.4612168 -0.9636142 -2.8226870
H -2.3898615 -2.2792975 -2.8013843
H -3.6459357 -3.2602364 -2.0372161
H 3.5977575 1.7946500 2.1166099
H 3.1973907 3.3319335 2.8913592

H 2.5697265 4.3151472 0.6870088
H 4.2127441 3.6660606 0.5796673
H 2.5332188 -1.7597274 -6.2719400
H 0.8128327 -3.4629678 -5.5868241
H -0.2782245 -3.2282179 -3.3542281
H 1.9739063 -4.8489646 4.7451818
H 2.7201996 -2.6550561 5.7068487
H 1.9213526 -0.5295644 4.6565052
H -0.5195983 -0.5768532 1.4852631

[**5**,1H$^+$]-IIIb
Total energy: –1988.4530863323 au
N -0.0947738 -0.1323017 -3.7006360
C 1.2169681 0.2006765 -3.8132796
C 1.9618708 0.0689653 -5.0273836
C 1.2684042 -0.4403222 -6.1540693
C -0.0710589 -0.7687481 -6.0356031
C -0.7118655 -0.5953522 -4.7862012
C 3.3430280 0.4580852 -5.0675680
C 3.9600811 0.9773719 -3.9601210
C 3.2422093 1.1368291 -2.7285412
C 1.8905083 0.7331545 -2.6558437
C 3.8076156 1.7043443 -1.5618622
C 3.0882235 1.8550735 -0.3892646
C 1.7309208 1.3945203 -0.3218735
N 1.2162094 0.8641655 -1.4665624
C 0.9368968 1.5189141 0.9171233
C 1.6754087 1.8440226 2.1038849
C 3.1891688 1.8721756 2.0988423
C 3.7084681 2.5200001 0.8142690
C 0.9905434 2.1080801 3.2849161
C -0.4009660 2.0397055 3.3535271
C -1.1292177 1.6590381 2.2227702
C -0.4810167 1.3737650 0.9935095
C -1.4092574 1.0647426 -0.1552240
C -1.9148393 -0.2328698 -0.4570077
C -2.8990260 -0.3818554 -1.4755025
C -3.3776487 0.7459193 -2.1432717
C -2.8888912 2.0239941 -1.8656772
C -1.9041850 2.1825949 -0.8820326
C -1.4451493 -1.4845326 0.2216207
C -1.5520387 -2.7288825 -0.4902467
C -2.2544501 -2.7760693 -1.8276539
C -3.4128052 -1.7643924 -1.8322193
C -1.0136655 -3.8587162 0.0977541
C -0.4352105 -3.7998817 1.3870452
C -0.4735313 -2.5389003 2.0637207
N -0.9462150 -1.4177943 1.4609574
C 0.0067048 -2.4744067 3.4453053
C 0.5798087 -3.6520988 4.0335415
C 0.6450837 -4.8788342 3.2880173
C 0.1413755 -4.9533311 2.0183194
C 1.0524532 -3.5614913 5.3667528
C 0.9340236 -2.3654038 6.0535964

C 0.3310281 -1.2709322 5.3934767
N -0.1184853 -1.3116818 4.1398636
O -1.3586814 3.3991031 -0.5456144
C -1.8223530 4.5802885 -1.2406139
O -2.4951642 1.5425190 2.2147693
C -3.2181771 1.7970379 3.4398071
H -1.6156683 4.5132673 -2.3174905
H -1.2572853 5.4102196 -0.8084564
H -2.8970680 4.7392227 -1.0778979
H -3.2734440 2.8780581 -2.4150996
H -4.1394947 0.6273676 -2.9136303
H -1.0475769 -4.8148029 -0.4267450
H 1.0880445 -5.7545430 3.7623118
H 0.1679568 -5.8916502 1.4642592
H -2.9052571 1.1055627 4.2335965
H -3.0814963 2.8349021 3.7743982
H -4.2700845 1.6266158 3.1948296
H -0.9017266 2.2604963 4.2910712
H 1.5549391 2.3708600 4.1787099
H 4.8417920 2.0459113 -1.5950656
H 3.8908418 0.3421204 -6.0016355
H 5.0036952 1.2843143 -3.9952986
H -4.1751067 -2.0882835 -1.1036155
H -3.9039701 -1.7391177 -2.8129082
H -1.5446834 -2.5218230 -2.6307523
H -2.6152066 -3.7932952 -2.0272755
H 3.5814921 0.8438484 2.1720160
H 3.5546107 2.4145245 2.9785592
H 3.4383137 3.5891893 0.8063468
H 4.8013945 2.4638972 0.7468853
H 1.7948372 -0.5611823 -7.0998895
H -0.6343679 -1.1551625 -6.8822790
H -1.7681207 -0.8437442 -4.6786706
H 1.4974888 -4.4382050 5.8371281
H 1.2839699 -2.2618158 7.0791930
H 0.2094772 -0.3213824 5.9197436
H 0.2333739 0.5583418 -1.4818551

[**5**,1H$^+$]-I′
Total energy: –1988.4539011923 au
C 3.6871782 -3.2113463 4.2933554
C 3.7635409 -2.6408612 3.0003002
C 2.5457747 -2.4413431 2.2894373
N 1.3689567 -2.7990137 2.8871638
C 1.2911418 -3.3423349 4.1146925
C 2.4595506 -3.5628075 4.8470960
C 2.5238324 -1.8743815 0.9674209
C 3.7711500 -1.5145835 0.3829617
C 4.9928666 -1.7124990 1.1112822
C 4.9944927 -2.2546256 2.3708694
N 1.3267362 -1.7257437 0.3512358
C 1.2847911 -1.2059310 -0.8830052
C 2.4992883 -0.8424339 -1.5771910
C 3.7137947 -0.9932406 -0.9319783

C -0.0222696 -1.0340299 -1.5774180
C 0.0056590 -1.0518418 -3.0045873
C 1.3273839 -1.1551812 -3.7424315
C 2.4078971 -0.3500107 -3.0002965
C -1.1920142 -0.9723986 -3.7129977
C -2.4172340 -0.8343861 -3.0567808
C -2.4475360 -0.7573944 -1.6600198
C -1.2579771 -0.8629464 -0.8968976
C -1.4051293 -0.8285370 0.5999105
C -1.0969399 0.2923791 1.4082348
C -1.1940774 0.1983933 2.8285717
C -1.6850819 -0.9726038 3.4043207
C -2.0504942 -2.0739612 2.6167167
C -1.8945461 -2.0079268 1.2247545
C -0.7473667 1.3656222 3.6909809
C 0.5032267 2.0249512 3.0772171
C 0.1940532 2.4406253 1.6566346
C -0.6505529 1.6058558 0.8501799
N -1.0851861 1.9829731 -0.3571700
C -0.6734252 3.1641627 -0.8824733
C 0.2542431 4.0134732 -0.1974275
C 0.6530932 3.6220092 1.1032707
C -1.1890012 3.5956080 -2.1832928
C -0.6811745 4.8088020 -2.7587736
C 0.2838691 5.6016277 -2.0478816
C 0.7232098 5.2252527 -0.8079927
N -2.1402880 2.8467275 -2.8050594
C -2.5835596 3.2488571 -3.9957567
C -2.1275728 4.4106666 -4.6587729
C -1.1756909 5.1946202 -4.0298483
O -2.1634214 -3.0632978 0.3851634
C -2.7526098 -4.2580694 0.9427618
O -3.6015746 -0.5605001 -0.9407262
C -4.8512164 -0.4705031 -1.6557537
H 4.6046486 -3.3743469 4.8563402
H 2.3898078 -4.0018826 5.8376335
H 0.2990084 -3.5856403 4.4816054
H 5.9254100 -2.4037920 2.9140124
H 5.9310158 -1.4252827 0.6381602
H -3.3294324 -0.7512633 -3.6398605
H -1.1720596 -1.0004677 -4.8024590
H 1.6513043 -2.2070752 -3.8178493
H 1.2100097 -0.7857671 -4.7685810
H 3.3842292 -0.4340394 -3.4933139
H 2.1299511 0.7170695 -3.0063508
H 4.6387674 -0.7213476 -1.4406549
H -5.6171008 -0.3281258 -0.8881348
H -5.0586711 -1.3937517 -2.2152598
H -4.8547985 0.3867502 -2.3430976
H -0.8036308 6.1079530 -4.4940548
H -2.5294937 4.6777181 -5.6345415
H -3.3487709 2.6247047 -4.4631264
H 0.6479826 6.5185103 -2.5113643
H 1.4386390 5.8407658 -0.2625206

H -2.4410471 -2.9683834 3.0941595
H -1.7829764 -1.0395038 4.4879057
H -1.5484528 2.1202276 3.7665594
H -0.5419893 1.0177833 4.7111031
H 0.8240141 2.8942674 3.6653059
H 1.3366326 1.3026920 3.0902365
H 1.3152808 4.2735591 1.6755079
H -2.9129678 -4.9267109 0.0927368
H -3.7138315 -4.0376951 1.4273074
H -2.0771079 -4.7392145 1.6644220
H 0.5158388 -2.6255844 2.3381729

[**5**,1H$^+$]-II′
Total energy: −1988.4546339008 au
N 0.8253661 -3.2423509 0.6359324
C 1.4431172 -3.4390291 -0.5672954
C 2.1581937 -4.6493645 -0.7874915
C 2.1879658 -5.5951615 0.2647786
C 1.5391542 -5.3411832 1.4703498
C 0.8526257 -4.1365507 1.6396990
C 2.7992741 -4.8395550 -2.0584547
C 2.7182455 -3.8770013 -3.0329189
C 1.9925986 -2.6553262 -2.8216902
C 1.3427842 -2.4220422 -1.5776235
C 1.8742742 -1.6231092 -3.7839054
C 1.1460785 -0.4795326 -3.5033784
C 0.5041274 -0.3534389 -2.2151198
N 0.6341053 -1.3077463 -1.2870890
C 1.0145383 0.6459963 -4.5002632
C 1.0392758 1.9947288 -3.7623838
C -0.0254927 2.0254061 -2.6829730
C -0.3031557 0.8579048 -1.9170468
C -0.7351751 3.1982006 -2.4270707
C -1.7312765 3.2432564 -1.4486013
C -2.0280470 2.0904824 -0.7092281
C -1.3100269 0.8835266 -0.9151482
C -1.7739847 -0.3189186 -0.1374975
C -1.6127624 -0.4468473 1.2670500
C -2.2481086 -1.5112855 1.9691152
C -2.9779983 -2.4629794 1.2535526
C -3.1258572 -2.3693071 -0.1343130
C -2.5507084 -1.2910265 -0.8217600
C -0.8178596 0.5230000 2.0791150
C -1.1793085 0.7215439 3.4542717
C -2.2656168 -0.1362745 4.0628190
C -2.1693464 -1.5623048 3.4861013
C -0.4886678 1.6807881 4.1715298
C 0.5656459 2.4118335 3.5717161
C 0.9108009 2.0785542 2.2224235
N 0.2127516 1.1577017 1.5114786
C 2.0481636 2.7600743 1.6010048
C 2.7278845 3.7845641 2.3426838
C 2.3189197 4.1037054 3.6826654
C 1.2855617 3.4331699 4.2784810

C 3.8083436 4.4511967 1.7122361
C 4.1777060 4.0901131 0.4281213
C 3.4609649 3.0512207 -0.2076488
N 2.4342269 2.4050551 0.3443988
O -2.7073273 -1.0932044 -2.1763848
C -3.4777060 -2.0542857 -2.9311365
O -3.0296933 2.0460320 0.2382977
C -3.7740656 3.2522532 0.5130209
H -4.3165993 3.5990591 -0.3778122
H -4.4894795 2.9803773 1.2939729
H -3.1159318 4.0528786 0.8785485
H -2.2796407 4.1664866 -1.2855167
H -0.5192065 4.0946729 -3.0082476
H 0.8814321 2.8202718 -4.4673850
H 2.0372490 2.1427889 -3.3168518
H 1.8127167 0.5827950 -5.2503437
H 0.0565803 0.5467616 -5.0384146
H 2.3627685 -1.7345308 -4.7521280
H 3.2084151 -4.0302561 -3.9936387
H 3.3484248 -5.7629401 -2.2309139
H -4.5160963 -2.1022215 -2.5743075
H -3.0246270 -3.0543355 -2.8828875
H -3.4608878 -1.6929883 -3.9629316
H -3.7132186 -3.1161790 -0.6603955
H -3.4616332 -3.2822817 1.7854121
H -1.2196873 -2.0149217 3.8192344
H -2.9752739 -2.1948186 3.8783727
H -3.2567438 0.2826075 3.8205625
H -2.1771020 -0.1428043 5.1567355
H -0.7461878 1.8765495 5.2135962
H 0.9888438 3.6676511 5.3009105
H 2.8561596 4.8859442 4.2187909
H 2.7258974 -6.5311563 0.1235805
H 1.5555487 -6.0603874 2.2835666
H 0.3312986 -3.8687694 2.5531004
H 4.3380504 5.2379234 2.2493264
H 5.0025795 4.5804832 -0.0857170
H 3.7470815 2.7349379 -1.2135407
H 0.3287606 -2.3460798 0.7309020

[**5**,1H$^+$]-IIIa′
Total energy: –1988.451555587 au
N 0.7141488 -1.3559185 -3.1773638
C 1.5783029 -0.3557736 -3.5049335
C 2.2030493 -0.2915496 -4.7978034
C 1.8643875 -1.2844818 -5.7502409
C 0.9802654 -2.2901997 -5.4024464
C 0.4446011 -2.2840022 -4.0958197
C 3.1644352 0.7335186 -5.0940096
C 3.5330581 1.6399740 -4.1392168
C 2.9382145 1.6148190 -2.8332503
C 1.9151215 0.6655156 -2.5093330
C 3.3647006 2.4869793 -1.8040969
C 2.7663222 2.4532712 -0.5594495

C 1.6506240 1.5668502 -0.3760371
N 1.2760244 0.6833721 -1.3094589
C 0.9109874 1.6377267 0.9214361
C 1.6601261 2.0455752 2.0654719
C 3.1144486 2.4447137 1.9003972
C 3.2684848 3.2718086 0.6087445
C 1.0272096 2.1403666 3.3031844
C -0.3439143 1.8772238 3.4403991
C -1.0960800 1.5376830 2.3074869
C -0.4775013 1.3879851 1.0344578
C -1.4040514 1.2008472 -0.1382814
C -2.0298421 -0.0185693 -0.5191532
C -3.0337698 -0.0062712 -1.5320944
C -3.3622830 1.1907131 -2.1677759
C -2.7300646 2.3892501 -1.8327743
C -1.7661199 2.3954487 -0.8164428
C -1.6683209 -1.3423720 0.0647344
C -2.0372236 -2.5511920 -0.6388821
C -2.7946395 -2.4623898 -1.9395155
C -3.7684264 -1.2794469 -1.9034513
C -1.6843544 -3.7769435 -0.1029275
C -0.9684273 -3.8553070 1.1141332
C -0.6534167 -2.6121167 1.7311348
N -0.9996320 -1.4062741 1.2226703
C 0.0687227 -2.6509370 2.9748716
C 0.4596514 -3.8813830 3.5772665
C 0.1160300 -5.1081377 2.9156929
C -0.5720031 -5.0904353 1.7300683
C 1.1678451 -3.8233318 4.8006707
C 1.4716925 -2.6006406 5.3924760
C 1.0672530 -1.4204763 4.7659642
N 0.3936301 -1.4804823 3.6033624
O -1.1274218 3.5443840 -0.3990212
C -1.4397359 4.7877965 -1.0635308
O -2.4574740 1.3418501 2.3399206
C -3.1511479 1.5056774 3.5969993
H -1.2004287 4.7389035 -2.1350118
H -0.8100444 5.5403397 -0.5810898
H -2.4979144 5.0555215 -0.9337821
H -3.0027273 3.3036307 -2.3516019
H -4.1265070 1.1911992 -2.9448209
H -1.9599106 -4.6960848 -0.6203882
H 0.4128200 -6.0482285 3.3761516
H -0.8310546 -6.0247153 1.2333962
H -2.7952705 0.7856144 4.3471405
H -3.0380203 2.5278453 3.9841731
H -4.2036746 1.3106780 3.3745116
H -0.8163517 1.9901052 4.4120358
H 1.5980306 2.4529459 4.1775355
H 4.1912615 3.1726118 -1.9969830
H 3.6136136 0.7558860 -6.0867911
H 4.2896161 2.3952202 -4.3523903
H -4.5637884 -1.4851211 -1.1670590
H -4.2612181 -1.1571003 -2.8756907

H -2.0748098 -2.3025429 -2.7571344
H -3.3136895 -3.4079996 -2.1407558
H 3.7625572 1.5546005 1.8357596
H 3.4449870 3.0201768 2.7738292
H 2.6845545 4.2024380 0.7041739
H 4.3139952 3.5624320 0.4432960
H 2.3166354 -1.2504260 -6.7413768
H 0.7055904 -3.0759527 -6.1037736
H -0.2330629 -3.0850217 -3.7920480
H 1.4755251 -4.7514205 5.2796614
H 2.0149424 -2.5445177 6.3309185
H 1.2644179 -0.4308156 5.1660348
H 0.0864391 -0.6149202 3.1378470

[**5**,1H$^+$]-IIIb′
Total energy: –1988.4486593171 au
N -0.4045681 -1.3165664 4.3111184
C -0.1983828 -2.4767739 3.6310901
C 0.3406984 -3.6440668 4.2707666
C 0.7012359 -3.5421524 5.6376400
C 0.5066473 -2.3469538 6.3082800
C -0.0619015 -1.2659786 5.5979970
C 0.4812993 -4.8739739 3.5411455
C 0.0807041 -4.9620453 2.2362651
C -0.4534285 -3.8181637 1.5526088
C -0.5550213 -2.5516080 2.2123202
N -0.9778409 -1.4369891 1.5626954
C -1.3744404 -1.5152966 0.2872126
C -1.4336355 -2.7709724 -0.4111255
C -0.9361899 -3.8928871 0.2255940
C -2.0664147 -2.8452919 -1.7823124
C -3.2281515 -1.8395558 -1.8580324
C -2.7380024 -0.4468645 -1.5027604
C -1.8029950 -0.2734708 -0.4365651
C -3.2030443 0.6693039 -2.2026585
C -2.7518777 1.9585927 -1.9003017
C -1.8095634 2.1330435 -0.8782473
C -1.3157410 1.0266538 -0.1369567
C -0.3644863 1.3400825 0.9883776
C 1.0464831 1.4974517 0.8645992
C 1.8158199 1.7982350 2.0328379
C 1.1721557 2.0202019 3.2482506
C -0.2156020 1.9420824 3.3624305
C -0.9755806 1.5932672 2.2429253
C 3.3282283 1.8680819 1.9674428
C 3.7685691 2.5941265 0.6932349
C 3.1309775 1.9498491 -0.5123949
C 1.7852708 1.4171784 -0.4311657
N 1.2001619 0.8892483 -1.5171072
C 1.8730670 0.8571718 -2.6931530
C 3.2122375 1.3101962 -2.8568703
C 3.8194176 1.8669895 -1.7087120
C 1.2027368 0.3479246 -3.8605386
C 1.8577123 0.2555605 -5.1232388

C 3.2215806 0.6898353 -5.2233835
C 3.8675594 1.2041347 -4.1295917
C 1.1175397 -0.2475079 -6.2186988
C -0.2133865 -0.6252904 -6.0677018
C -0.8174870 -0.5031451 -4.8159317
N -0.1065508 -0.0358409 -3.7736172
O -2.3455566 1.4832799 2.2731649
C -3.0133263 1.6020438 3.5478829
O -1.2952428 3.3607013 -0.5286313
C -1.7427099 4.5285064 -1.2528175
H -1.4993211 4.4509730 -2.3216297
H -1.1991066 5.3686887 -0.8123887
H -2.8237155 4.6833284 -1.1299661
H -3.1313347 2.8050976 -2.4649052
H -3.9326100 0.5358640 -3.0014981
H -0.9353328 -4.8557778 -0.2874476
H 0.8941505 -5.7414875 4.0559011
H 0.1596156 -5.9039481 1.6933442
H -2.6362893 0.8528633 4.2563202
H -2.8943741 2.6101752 3.9703211
H -4.0713854 1.4181970 3.3400860
H -0.6870705 2.1284357 4.3220915
H 1.7665084 2.2632912 4.1288779
H 4.8391703 2.2464570 -1.7746543
H 3.7230160 0.6133849 -6.1858831
H 4.8977090 1.5483297 -4.2125004
H -4.0179821 -2.1556762 -1.1561730
H -3.6803176 -1.8366489 -2.8579189
H -1.3192006 -2.6128267 -2.5595881
H -2.4134559 -3.8672576 -1.9812007
H 3.7554017 0.8508572 1.9694098
H 3.7180494 2.3773013 2.8571433
H 3.4471286 3.6484109 0.7468380
H 4.8605075 2.5975429 0.5844660
H 1.6004643 -0.3307179 -7.1908706
H -0.7937638 -1.0062038 -6.9024735
H -1.8543541 -0.7601497 -4.6264204
H 1.1202623 -4.4102492 6.1463172
H 0.7702741 -2.2347846 7.3584539
H -0.2461238 -0.3191178 6.1109929
H -0.5454942 0.0633004 -2.8484107

[**6**,1H^{+}]-I
Total energy: –1988.4486593171 au
N -1.3114381 -1.5852107 3.8750569
C -0.0550790 -2.0150040 3.5770122
C 0.8752707 -2.4105422 4.5950422
C 0.4559861 -2.3164902 5.9458156
C -0.8199216 -1.8639624 6.2338529
C -1.6679056 -1.5172513 5.1579537
C 2.1802873 -2.8944228 4.2372706
C 2.5565639 -2.9969648 2.9258169
C 1.6652023 -2.6025826 1.8719531
C 0.3648063 -2.0836155 2.1762117

C 2.0109371 -2.7301997 0.5049444
C 1.1334604 -2.3264117 -0.4854459
C -0.1098340 -1.7406036 -0.0781874
N -0.4798687 -1.6566668 1.2048972
C 1.4039225 -2.5196398 -1.9601016
C 0.0980796 -2.9342374 -2.6637208
C -1.0044124 -1.9259892 -2.3898612
C -1.0617412 -1.2711152 -1.1298058
C -1.9687259 -1.6520017 -3.3688534
C -3.0005306 -0.7471046 -3.1232440
C -3.0273962 -0.0647945 -1.9065449
C -2.0455952 -0.2581036 -0.9088665
O -4.0237421 0.8968120 -1.6991918
C -5.0262130 0.5125202 -0.7446176
O -4.4565079 -0.0538823 0.4465861
C -3.3800130 0.6693493 0.9689288
C -3.5586141 1.3515958 2.1735747
C -2.4776077 2.0106660 2.7558850
C -1.2363517 2.0512270 2.1128254
C -1.0745057 1.4008429 0.8555733
C -2.1370516 0.6139134 0.3105919
C 0.2025087 1.6525766 0.1709999
C 1.3816826 1.9958859 0.8980397
C 1.2628560 2.1189571 2.3972599
C -0.0749381 2.7975541 2.7423323
C 2.5606761 2.2364708 0.2091334
C 2.6254514 2.1812915 -1.2069338
C 1.4254664 1.8936583 -1.8983208
N 0.2868807 1.6422453 -1.1825651
C 3.8179490 2.4258228 -1.9671261
C 3.7959819 2.3881436 -3.3377959
C 2.5819487 2.1140777 -4.0562034
C 1.3731539 1.8697631 -3.3365095
N 0.1763594 1.6181860 -3.9297637
C 0.1420624 1.5923806 -5.2612815
C 1.2813750 1.8108103 -6.0729609
C 2.5006940 2.0729772 -5.4714230
H -3.7704288 -0.5471392 -3.8660052
H -1.9186926 -2.1658218 -4.3280955
H -0.2064475 -3.9306139 -2.3022040
H 0.2533204 -3.0248581 -3.7458426
H 2.1880785 -3.2724032 -2.1079390
H 1.7731280 -1.5815830 -2.4069333
H 2.9746027 -3.1661489 0.2380815
H 2.8624657 -3.1892809 5.0344883
H 3.5416737 -3.3798000 2.6596532
H -5.5764987 1.4364355 -0.5260927
H -4.5350552 1.3350485 2.6541684
H -2.6008656 2.5119173 3.7144511
H -0.0580613 3.8405594 2.3855642
H -0.2122480 2.8382578 3.8292484
H 2.1113934 2.6859730 2.7955884
H 1.2939087 1.1183178 2.8565942
H 3.4631830 2.4855683 0.7665839

H 4.7062133 2.5731950 -3.9063137
H 4.7400084 2.6436829 -1.4317126
H -5.6815666 -0.2715908 -1.1441533
H 3.3945219 2.2482871 -6.0684772
H 1.1854204 1.7723002 -7.1557492
H -0.8258673 1.3911165 -5.7214617
H 1.1429125 -2.6055864 6.7409795
H -1.1750514 -1.7800888 7.2593396
H -2.6845733 -1.1724976 5.3599265
H -0.5470431 1.4558227 -1.7519199

[**6**,1H^+]-I′
Total energy: –1947.9651642976 au
C -1.311438 -1.585211 3.875057
C -0.002210 -2.033090 3.564470
C 0.829371 -2.398597 4.666156
N 0.438647 -2.327413 5.966089
C -0.797497 -1.897705 6.215247
C -1.708695 -1.519147 5.199874
C 2.160238 -2.867808 4.382735
C 2.652737 -2.938726 3.058677
C 1.786922 -2.557861 1.979305
C 0.508250 -2.127111 2.224437
N 2.971899 -3.244848 5.417597
C 4.247296 -3.686008 5.283052
C 4.794627 -3.715000 3.965293
C 3.996371 -3.360773 2.888350
C 6.247648 -4.096818 3.824316
C 7.049517 -3.459513 4.973277
C 6.461604 -3.842647 6.318539
C 5.057586 -4.040532 6.458007
C 4.515470 -4.499075 7.699424
C 5.391793 -4.559988 8.799325
C 6.760450 -4.312960 8.679804
C 7.293051 -3.988464 7.433582
O 4.893608 -4.965292 10.041213
C 4.081856 -3.980927 10.701840
O 3.117937 -3.386497 9.817898
C 2.422460 -4.305308 9.022608
C 1.072911 -4.534878 9.292125
C 0.352812 -5.390752 8.459827
C 0.981446 -6.064285 7.404096
C 2.368609 -5.872399 7.161517
C 3.090451 -4.911140 7.934758
C 3.005231 -6.752560 6.135615
C 2.193917 -7.273520 5.074869
C 0.711199 -6.979167 5.074472
C 0.188337 -7.013668 6.522789
N 4.295604 -7.075610 6.280021
C 4.892628 -7.879236 5.365165
C 4.186573 -8.377514 4.222302
C 2.809406 -8.064814 4.121500
C 6.297278 -8.252085 5.541786
C 6.930449 -9.049691 4.531190

C 6.184943 -9.497391 3.387076
C 4.861129 -9.182718 3.243878
C 8.296632 -9.381823 4.711373
C 8.961735 -8.942458 5.842869
C 8.243001 -8.178291 6.789608
N 6.960565 -7.839136 6.656018
H 0.601109 -4.027888 10.131642
H -0.709305 -5.551713 8.640547
H 0.269244 -8.042471 6.911653
H -0.874880 -6.745904 6.557018
H 0.179359 -7.703705 4.445504
H 0.519806 -5.981836 4.644797
H 2.231548 -8.468168 3.288852
H 6.694751 -10.103943 2.638707
H 4.296437 -9.538453 2.382238
H 4.686680 -3.140706 11.065713
H 7.395934 -4.404721 9.558679
H 8.365190 -3.832564 7.326307
H 7.045215 -2.363166 4.857376
H 8.097809 -3.777248 4.928262
H 6.626938 -3.775673 2.848000
H 6.350897 -5.192451 3.870784
H 4.411883 -3.396097 1.881662
H -0.142248 -1.841868 1.398927
H 2.168211 -2.616717 0.961720
H 3.594188 -4.522135 11.522341
H -1.988205 -1.297979 3.071552
H -2.705875 -1.180099 5.471533
H -1.094605 -1.844714 7.263140
H 8.805409 -9.982702 3.957835
H 10.010218 -9.179666 6.013720
H 8.742419 -7.833699 7.697957
H 1.074280 -2.600760 6.699109

[**1**,2H$^+$]-I
Total energy: –1649.5145808462 au
C -0.1413914 -2.6237275 -3.3834514
C 0.7741578 -2.3493895 -2.3453172
C 1.6379897 -3.3641957 -1.8319688
C 1.5500946 -4.6696524 -2.3876984
C 0.6464316 -4.9336701 -3.4010606
C -0.1984981 -3.9082885 -3.8998397
N 0.8649327 -1.0881005 -1.7913720
C 1.7026641 -0.7270681 -0.7894881
C 2.5980336 -1.7169404 -0.2856997
C 2.5452980 -3.0010258 -0.8045197
C 1.7117345 0.6562531 -0.2640022
C 2.9286159 1.0762392 0.3492832
C 4.0915308 0.1151711 0.4930545
C 3.5864198 -1.3080862 0.7799231
C 3.0391697 2.3941237 0.8020724
C 1.9847018 3.2988110 0.6910716
C 0.7653171 2.8829886 0.1271965
C 0.6055392 1.5554386 -0.3527590

C -0.7384758 1.2084925 -0.9480586
C -0.9901052 1.6756365 -2.2658246
C -2.2165678 1.4065320 -2.8990050
C -3.1864845 0.6561855 -2.2369127
C -2.9842838 0.1715193 -0.9414937
C -1.7613742 0.4742756 -0.2734588
C -4.0581274 -0.6470134 -0.2533948
C -3.4280168 -1.7294207 0.6374108
C -2.4536399 -1.0888168 1.5962024
C -1.6594461 -0.0020216 1.1238247
N -0.8328337 0.6044080 2.0097614
C -0.6655094 0.2388140 3.3304234
C -1.4299955 -0.8651834 3.8160700
C -2.3217326 -1.4982856 2.9135962
C -1.2658224 -1.2546455 5.1732676
C -0.3844300 -0.5702176 5.9904907
C 0.3629314 0.5263542 5.4874449
C 0.2294850 0.9351675 4.1702177
O 0.0378717 2.3528440 -2.8603407
C -0.1624264 2.9097875 -4.1874531
O -0.3392431 3.6828278 0.0323261
C -0.2456169 5.0665397 0.4659623
H -0.9899652 3.6302457 -4.1853573
H -0.3526590 2.1165844 -4.9218459
H 0.7743522 3.4183633 -4.4256043
H -0.7878223 -1.8377263 -3.7707471
H -0.9013492 -4.1331960 -4.6992442
H 0.5793032 -5.9335853 -3.8244423
H 2.2034133 -5.4503837 -2.0028254
H 3.2247292 -3.7591899 -0.4161074
H 3.0992694 -1.3360384 1.7681527
H 4.4156560 -2.0234352 0.8111902
H 4.7535816 0.4560499 1.2970263
H 4.6894252 0.1111168 -0.4330042
H 3.9709356 2.7217630 1.2603335
H 2.1090346 4.3137204 1.0565826
H 0.5164031 5.6044231 -0.1118265
H -0.0209638 5.1276443 1.5385869
H -1.2321960 5.4919481 0.2694064
H -4.6913502 0.0112431 0.3635375
H -4.7120668 -1.1027201 -1.0055483
H -4.1942046 -2.2767525 1.1973969
H -2.9007301 -2.4654194 0.0087426
H -2.9245814 -2.3315011 3.2735890
H -1.8444225 -2.0946860 5.5529592
H -0.2600446 -0.8696053 7.0289698
H 1.0503885 1.0546130 6.1447603
H 0.8011508 1.7793333 3.7876931
H -4.1235627 0.4364679 -2.7458705
H -2.4105045 1.7627272 -3.9062365
H 0.2497132 -0.3678746 -2.1737643
H -0.2898312 1.4032096 1.6770167

[**1**,2H^{+}]-II
Total energy: −1649.5143800786 au
C -1.5819313 -4.8044956 -3.0142604
C -0.8557624 -3.9945264 -2.0991975
C -1.1522562 -2.5992518 -2.0457311
C -2.1417260 -2.0289153 -2.8738556
C -2.8279832 -2.8493779 -3.7546849
C -2.5480745 -4.2388437 -3.8265190
N -0.4354033 -1.8236520 -1.1563855
C 0.5432064 -2.2686302 -0.3310589
C 0.8378231 -3.6651159 -0.3519636
C 0.1460474 -4.4884189 -1.2263183
C 1.8936890 -4.1941490 0.5885551
C 1.8293898 -3.4343792 1.9216784
C 1.9214702 -1.9406133 1.6840883
C 1.2738742 -1.3420462 0.5638056
C 2.6459327 -1.1296620 2.5621260
C 2.7709264 0.2429303 2.3586761
C 2.1515610 0.8438369 1.2496745
C 1.3748157 0.0694701 0.3462497
C 0.8126200 0.7857931 -0.8577749
C -0.2530903 1.7403349 -0.8032345
C -0.6511783 2.4351639 -1.9827956
C 0.0086252 2.1650654 -3.1848506
C 1.0595613 1.2531400 -3.2557885
C 1.4733977 0.5731621 -2.0978316
C -0.9854695 2.0955517 0.4340644
C -1.6887197 3.3321907 0.5495646
C -1.7045870 4.2668617 -0.6360441
C -1.7720631 3.4539258 -1.9372595
N -1.0186588 1.2556808 1.4972595
C -1.6455730 1.5066270 2.7016624
C -2.3334253 2.7490723 2.8461478
C -2.3354173 3.6330156 1.7379085
C -1.6036861 0.5665224 3.7526724
C -2.2422044 0.8699183 4.9444554
C -2.9297651 2.1001858 5.1112926
C -2.9780511 3.0240399 4.0829076
O 2.5103603 -0.3157874 -2.0744769
C 3.2499354 -0.5663261 -3.3006619
O 2.2455713 2.1759906 0.9624993
C 3.0447898 3.0273343 1.8282494
H 3.7197514 0.3551543 -3.6663090
H 4.0179674 -1.2922346 -3.0249507
H 2.5959547 -0.9902618 -4.0733807
H 1.5613166 1.0885252 -4.2044182
H -0.2960817 2.6919861 -4.0875691
H -1.7034120 4.1163938 -2.8072831
H -2.7473629 2.9440582 -1.9998208
H -2.5540163 4.9541494 -0.5542384
H -0.7889397 4.8801635 -0.6311755
H -2.8656938 4.5807320 1.8275666
H -3.5048109 3.9688870 4.2031119
H -3.4217215 2.3124915 6.0581248

H -2.2158774 0.1540008 5.7633066
H -1.0810753 -0.3801160 3.6245124
H 4.0916003 2.6991983 1.8367192
H 2.6424222 3.0364545 2.8492373
H 2.9705929 4.0253520 1.3907938
H 3.3614360 0.8344734 3.0515768
H 3.1386146 -1.5840559 3.4201014
H 0.8847171 -3.6766393 2.4355653
H 2.6420825 -3.7527628 2.5840909
H 2.8889456 -4.0594557 0.1348729
H 1.7500501 -5.2700238 0.7388239
H 0.3769082 -5.5531602 -1.2463514
H -1.3629606 -5.8695462 -3.0624853
H -3.1018723 -4.8593308 -4.5279071
H -3.5924520 -2.4215427 -4.3999185
H -2.3612651 -0.9639579 -2.8163389
H -0.6432137 -0.8232856 -1.1498383
H -0.5101756 0.3727304 1.4207367

[**1**,2H^{+}]-III
Total energy: –1649.5133816897 au
C -4.3089481 -0.1675678 1.4213871
C -2.9564243 -0.2464190 2.0872823
C -1.7884494 0.0215604 1.3102497
C -1.8804100 0.3811543 -0.1236340
C -3.1135437 0.0712407 -0.7691179
C -4.2120759 -0.6617898 -0.0275497
C -0.8392672 1.0311012 -0.8572200
C -1.0915224 1.4254294 -2.2000053
C -2.3143439 1.1188487 -2.8214777
C -3.2971684 0.4396485 -2.1048572
C -2.8176182 -0.5610158 3.4297675
C -1.5535409 -0.6245904 4.0674351
C -0.3988532 -0.3537687 3.2739446
N -0.5905801 -0.0541475 1.9402581
C 0.8963382 -0.3907641 3.8329032
C 1.0356260 -0.6967867 5.1770208
C -0.0991119 -0.9713025 5.9838123
C -1.3714004 -0.9376451 5.4420652
O -0.0698419 2.0909231 -2.8213652
C -0.2576693 2.5484022 -4.1885514
C 0.4910259 1.4649905 -0.2842578
C 0.5049581 2.7393223 0.3422847
C 1.7007095 3.2770599 0.8463979
C 2.8855138 2.5539105 0.7286192
C 2.9254082 1.2948591 0.1237855
C 1.7193732 0.7342870 -0.3975679
C 1.8494186 -0.6038157 -1.0257303
C 2.9699018 -1.4477830 -0.7573504
C 4.0461269 -0.9513062 0.1776651
C 4.2429680 0.5587427 -0.0071956
C 3.0378985 -2.6938562 -1.3606807
C 2.0336100 -3.1651323 -2.2424343
C 0.9270329 -2.3028630 -2.5045645

N 0.9076728 -1.0728117 -1.8799928
C -0.1127849 -2.6912020 -3.3764112
C -0.0467308 -3.9366381 -3.9795705
C 1.0432985 -4.8112297 -3.7310683
C 2.0666223 -4.4354314 -2.8797440
O -0.7017021 3.3717398 0.4173418
C -0.7683310 4.6906848 1.0235529
H -0.4582957 4.6536727 2.0755612
H -1.8192767 4.9803632 0.9557989
H -0.1466653 5.4052706 0.4695344
H 1.7129992 4.2505308 1.3271542
H 3.8064727 2.9811601 1.1219581
H 3.8928473 -3.3364396 -1.1522686
H -0.9502182 -2.0224332 -3.5686889
H -0.8424073 -4.2480902 -4.6531042
H 1.0684335 -5.7841834 -4.2172929
H 2.9060627 -5.1003496 -2.6852078
H -0.4180505 1.7010716 -4.8674298
H -1.0995395 3.2489005 -4.2511142
H 0.6729223 3.0589148 -4.4460017
H -2.5052697 1.4137594 -3.8488826
H -4.2395706 0.2018157 -2.5953469
H -3.7104811 -0.7650202 4.0201064
H 1.7694201 -0.1791258 3.2176564
H 2.0292831 -0.7267390 5.6190477
H 0.0391459 -1.2088532 7.0364957
H -2.2464609 -1.1461868 6.0547555
H 4.6736095 0.7515599 -1.0035462
H 4.9573517 0.9458384 0.7278769
H 3.7525172 -1.1631216 1.2189959
H 4.9780004 -1.4973773 -0.0067138
H -4.0029151 -1.7441066 -0.0323388
H -5.1668311 -0.5197327 -0.5458370
H -4.6563958 0.8784371 1.4327693
H -5.0376809 -0.7548969 1.9913513
H 0.2442004 0.1596861 1.3896289
H 0.1036073 -0.4703636 -2.0703436

[**2**,2H$^+$]-I
Total energy: –1609.0206084642 au
C -0.1211716 -2.1123274 3.5850724
C 0.3352083 -2.1979484 2.2528891
C 1.6012345 -2.7826520 1.9384094
C 2.4011054 -3.2727413 3.0065554
C 1.9498456 -3.1815248 4.3108050
C 0.6868039 -2.6018094 4.5988209
N -0.4297087 -1.7203538 1.2074775
C -0.0717825 -1.7526632 -0.0987094
C 1.1647801 -2.3725544 -0.4401702
C 1.9739655 -2.8634330 0.5722534
C -0.9812415 -1.2343091 -1.1386513
C -0.8814041 -1.8558500 -2.4154960
C 0.2248814 -2.8546687 -2.6930418
C 1.5001804 -2.5017377 -1.9069705

C -1.8302231 -1.5568751 -3.4035330
C -2.8836768 -0.6822020 -3.1459408
C -2.9546840 -0.0374395 -1.9065110
C -1.9801737 -0.2333427 -0.9068081
O -3.9658713 0.8922620 -1.6857596
C -4.9586892 0.4919075 -0.7222156
O -4.3769535 -0.0660472 0.4712939
C -3.3095218 0.6555407 0.9957190
C -3.4683059 1.3074292 2.2233542
C -2.3822291 1.9680308 2.7937996
C -1.1540077 2.0523083 2.1225444
C -1.0078165 1.4283511 0.8514106
C -2.0733819 0.6322234 0.3182327
C 0.2384383 1.7355945 0.1239849
C 1.4243533 2.1156032 0.8160631
C 1.3530834 2.2166471 2.3212770
C -0.0001025 2.8263686 2.7292882
N 0.2611205 1.7392964 -1.2305114
C 1.3648322 2.0338371 -2.0056879
C 2.5798455 2.3717133 -1.3324837
C 2.5631839 2.4154164 0.0852387
C 3.7256705 2.6697064 -2.1194349
C 3.6493802 2.6316489 -3.4999684
C 2.4304839 2.2993745 -4.1467158
C 1.2921888 2.0029038 -3.4142882
H -3.6420459 -0.4746784 -3.8980706
H -1.7529463 -2.0350874 -4.3783658
H -0.1122494 -3.8656415 -2.4120487
H 0.4334795 -2.8795539 -3.7687296
H 2.2727530 -3.2653623 -2.0468397
H 1.9148178 -1.5529442 -2.2839113
H 2.9227065 -3.3341371 0.3161512
H -1.0922109 -1.6718770 3.8046285
H 0.3469245 -2.5410453 5.6305316
H 2.5629478 -3.5578736 5.1268640
H 3.3667706 -3.7197238 2.7781440
H -5.5189044 1.4075059 -0.5014662
H -4.4333646 1.2708900 2.7245721
H -2.4927486 2.4472876 3.7648577
H -0.0454594 3.8761424 2.3968071
H -0.1007360 2.8369411 3.8206309
H 2.1857840 2.8228423 2.6937075
H 1.4576946 1.2165356 2.7718131
H 3.4738800 2.7022034 0.6102777
H 0.3555627 1.7526286 -3.9094822
H 2.3882648 2.2771502 -5.2336315
H 4.5274383 2.8592208 -4.1005960
H 4.6556596 2.9283106 -1.6167868
H -5.6001965 -0.3032985 -1.1185201
H -1.3515531 -1.3485126 1.4419598
H -0.6119089 1.5463935 -1.7240882

[**3**,2H^{+}]-I

Total energy: –1956.7817104919 au

C -1.1664180 -1.6104419 -4.0320482
C -0.3598904 -2.5852538 -3.3910043
C -0.4555975 -3.9535812 -3.8222633
C -1.3580660 -4.2843165 -4.8650330
C -2.1380374 -3.3103070 -5.4692289
C -2.0384065 -1.9648967 -5.0506347
C 0.5680192 -2.2908441 -2.3244470
C 1.3782740 -3.3084972 -1.7477932
C 1.2475492 -4.6575126 -2.2128282
C 0.3599485 -4.9636643 -3.2068491
N 0.7110715 -1.0154442 -1.8269440
C 1.5756276 -0.6355715 -0.8459770
C 2.4392580 -1.6269259 -0.3103240
C 2.3116582 -2.9354291 -0.7539731
C 1.6513679 0.7687660 -0.3828661
C 2.9234311 1.1748235 0.1197901
C 4.0508176 0.1707911 0.2571214
C 3.4929359 -1.1985476 0.6819662
C 3.1127194 2.5100637 0.4827913
C 2.0760640 3.4400730 0.4176030
C 0.7976183 3.0321283 0.0006839
C 0.5637424 1.6920198 -0.4170457
C -0.8285711 1.3673868 -0.8969835
C -1.2226993 1.9361612 -2.1403302
C -2.5192017 1.7330373 -2.6426873
C -3.4101051 0.9151800 -1.9490650
C -3.0571649 0.3030807 -0.7445183
C -1.7719554 0.5680030 -0.1845856
C -4.0234419 -0.6289914 -0.0409989
C -3.2563281 -1.7874349 0.6193223
C -2.2086367 -1.2167363 1.5440190
C -1.5260922 -0.0379619 1.1438343
N -0.6733746 0.5321256 2.0393368
C -0.3770492 0.0442676 3.2918089
C -0.9982451 -1.1762983 3.6775939
C -1.9191085 -1.7685428 2.7836788
C -0.6992930 -1.7414610 4.9597273
C 0.1676599 -1.1110266 5.8084982
C 0.7920445 0.1336697 5.4552812
C 0.5218609 0.7420805 4.1807699
C 1.1397456 1.9809474 3.8722881
C 1.9960446 2.5942737 4.7745334
C 2.2682851 1.9923279 6.0229734
C 1.6739192 0.7847104 6.3549911
O -0.2611941 2.6480931 -2.8004062
C -0.6076542 3.3091438 -4.0453381
O -0.2915825 3.8572950 -0.0079634
C -0.1177761 5.2537520 0.3482025
H 0.3026470 3.8271211 -4.3553408
H -1.4166985 4.0339680 -3.8901487
H -0.8974612 2.5783514 -4.8116685
H 0.2623103 -5.9897459 -3.5581868
H 1.8713513 -5.4254144 -1.7598932
H 2.9604344 -3.7040200 -0.3351071

H 3.0561221 -1.1242308 1.6910836
H 4.2850516 -1.9538281 0.7307438
H 4.7791432 0.5330316 0.9914088
H 4.5864290 0.0649678 -0.7003014
H 4.0897450 2.8305851 0.8409723
H 2.2605110 4.4679746 0.7150167
H -1.1079122 5.7011799 0.2364671
H 0.5928484 5.7440215 -0.3291779
H 0.2198050 5.3558523 1.3878849
H -4.5897544 -0.0808013 0.7294016
H -4.7547544 -1.0135626 -0.7608190
H -3.9307113 -2.4418803 1.1825569
H -2.7797918 -2.4074318 -0.1572438
H -2.4262496 -2.6830327 3.0897193
H -1.1796233 -2.6770752 5.2389491
H 0.3946853 -1.5436178 6.7816830
H -4.4000278 0.7371250 -2.3661928
H -2.8280116 2.1862171 -3.5798340
H 1.8747778 0.3184978 7.3184630
H 2.9416519 2.4815421 6.7242975
H 2.4578809 3.5464656 4.5211562
H 0.9451474 2.4839852 2.9275198
H -1.4258267 -5.3223201 -5.1873202
H -2.8250164 -3.5785085 -6.2696437
H -2.6465370 -1.2019365 -5.5324790
H -1.1100114 -0.5618570 -3.7477690
H -0.2512323 1.4205279 1.7678898
H 0.1553049 -0.2766656 -2.2587660

[**3**,2H$^+$]-II
Total energy: –1956.7822038212 au
C -0.1249956 -0.7288095 6.2844164
C 0.6482139 0.0513090 5.3873459
C 0.4326253 -0.0938895 3.9734543
C -0.5609510 -1.0010719 3.5244109
C -1.3047570 -1.7464515 4.4271745
C -1.0825191 -1.6150847 5.8158553
C 1.6291189 0.9804031 5.8763753
C 2.3741936 1.7478324 5.0244419
C 2.2052629 1.6286618 3.6066144
C 1.2519689 0.7065941 3.0950176
C 2.9432917 2.4029044 2.6828872
C 2.7857678 2.2740502 1.3101504
C 1.8680086 1.3106401 0.8136783
N 1.1462030 0.6056514 1.7273591
C 3.5455533 3.1284460 0.3251059
C 2.6396553 3.4550099 -0.8719706
C 2.0940024 2.1768778 -1.4772119
C 1.7024797 1.0900498 -0.6420381
C 1.2319793 -0.1304388 -1.2229560
C 1.1758538 -0.2275032 -2.6419059
C 1.5327037 0.8632659 -3.4510094
C 1.9852420 2.0427041 -2.8633245
C 0.9518534 -1.3886094 -0.4394073

C -0.3149148 -2.0540230 -0.4113502
C -0.4442448 -3.3216924 0.2278958
C 0.6723491 -3.8882445 0.8468903
C 1.9159216 -3.2602733 0.8294150
C 2.0660589 -2.0273539 0.1744352
C -1.7704194 -4.0557212 0.2210840
C -2.4637419 -3.8823694 -1.1388441
C -2.5893577 -2.4118249 -1.4533743
C -1.5432478 -1.5362883 -1.0582483
C -3.6948492 -1.8826987 -2.1043103
C -3.8304620 -0.5006606 -2.3668736
C -2.7992726 0.3692944 -1.9187805
N -1.7185203 -0.2069202 -1.2927505
C -4.9637264 0.0489932 -3.0500448
C -5.0507354 1.3955009 -3.2721369
C -4.0302524 2.2993908 -2.8178495
C -2.8806057 1.7970159 -2.1159286
C -4.1411328 3.6947830 -3.0454611
C -3.1653572 4.5707683 -2.5953354
C -2.0427450 4.0783872 -1.8935725
C -1.9032326 2.7182789 -1.6598984
O 3.2635397 -1.3765774 0.0668471
C 4.4543908 -1.9957290 0.6235782
O 0.7759581 -1.4345563 -3.1441176
C 0.7444316 -1.6223769 -4.5846948
H 0.4183929 -2.6548373 -4.7289200
H 1.7429998 -1.4822862 -5.0169669
H 0.0291579 -0.9359094 -5.0552705
H -1.6696067 -2.2064622 6.5159952
H 2.2782706 2.8741318 -3.5023528
H 4.4386807 2.5852090 -0.0239873
H 3.8953846 4.0415375 0.8197229
H 1.4859561 0.7889205 -4.5331003
H 1.8135979 4.1030038 -0.5362753
H 3.1943630 4.0152670 -1.6328171
H 1.7652193 1.0662577 6.9533767
H -2.0677367 -2.4320414 4.0641018
H 3.1084758 2.4566014 5.4020050
H -0.7781201 -1.1188193 2.4642507
H 0.0445395 -0.6164731 7.3542958
H 3.6593360 3.1282214 3.0681383
H 5.2673892 -1.3032183 0.3943679
H 4.6458586 -2.9663883 0.1492716
H 4.3630813 -2.1176136 1.7103522
H -3.2646220 5.6395289 -2.7757637
H 0.5736377 -4.8536580 1.3405279
H -1.8697465 -4.3787529 -1.9232434
H -3.4558138 -4.3477187 -1.1415119
H 2.7659800 -3.7441901 1.3003929
H -2.4273537 -3.6666510 1.0160142
H -1.6050916 -5.1172839 0.4362407
H -5.9087786 1.8102673 -3.7989868
H -1.2861322 4.7705942 -1.5297046
H -5.7451813 -0.6285593 -3.3881793

H -1.0319076 2.3835133 -1.0998690
H -5.0129351 4.0694289 -3.5798760
H -4.4919863 -2.5529790 -2.4244578
H -0.9705681 0.4181930 -0.9930267
H 0.4786727 -0.0744777 1.3642180

[**3**,2H$^+$]-III
Total energy: −1956.7778562814 au
C 1.8436519 -0.2949035 3.1665959
C 0.6111609 -0.3701145 3.8634092
C 0.6296045 -0.7038406 5.2618305
C 1.8742948 -0.9535228 5.8939261
C 3.0631228 -0.8744378 5.1856439
C 3.0454014 -0.5405598 3.8136955
C -0.5982613 -0.7770743 6.0040336
C -1.8052436 -0.5305517 5.4117018
C -1.8744179 -0.1965458 4.0202533
C -0.6765306 -0.1239538 3.2577422
C -3.0973666 0.0588350 3.3624487
C -3.1665549 0.3734682 2.0129477
C -1.9619768 0.4468593 1.2626115
N -0.7985325 0.1878888 1.9226764
C -1.9751850 0.8023602 -0.1761369
C -3.2292162 0.6618037 -0.8398793
C -4.4306821 0.0958016 -0.1118466
C -4.4831804 0.6337598 1.3233569
C -3.3466108 1.0618382 -2.1740446
C -2.2691079 1.5913771 -2.8796106
C -1.0229722 1.7237249 -2.2445826
C -0.8465925 1.3162625 -0.8888767
C 0.4762284 1.7102400 -0.2710010
C 1.7416597 1.0856166 -0.5314312
C 2.9409269 1.7464644 -0.1206428
C 2.8588556 2.9457715 0.5899002
C 1.6364357 3.5307501 0.9075689
C 0.4492351 2.9295633 0.4635807
C 1.9250229 -0.2417424 -1.1772233
C 3.1659967 -0.9353442 -1.1067401
C 4.3347604 -0.3238774 -0.3740377
C 4.3022912 1.1997055 -0.4965944
C 3.2741258 -2.1858736 -1.6974527
C 2.1967969 -2.8122550 -2.3578348
C 0.9554444 -2.1221139 -2.4075501
N 0.9023809 -0.8764686 -1.8223285
C -0.1976804 -2.7120208 -3.0481012
C -0.0320299 -4.0112581 -3.6425341
C 1.2393054 -4.6772822 -3.5840161
C 2.3145067 -4.1048654 -2.9641345
C -1.1407317 -4.6236664 -4.2803338
C -2.3731869 -3.9910607 -4.3339596
C -2.5374157 -2.7176873 -3.7462919
C -1.4707477 -2.0927257 -3.1171517
O -0.7889976 3.4661961 0.6789571
C -0.8947528 4.7267250 1.3939404

O 0.0786141 2.2547285 -2.8534444
C -0.0314015 2.7279751 -4.2236562
H -0.4999740 4.6334465 2.4135994
H -1.9646010 4.9439761 1.4284899
H -0.3684983 5.5247424 0.8553068
H 1.6142689 4.4594161 1.4693886
H 3.7781736 3.4366780 0.9050380
H 4.2271445 -2.7114293 -1.6456530
H 1.3289366 -5.6596685 -4.0451283
H 3.2747333 -4.6144859 -2.9164262
H -0.2939410 1.9068306 -4.9024482
H -0.7728731 3.5331022 -4.2977815
H 0.9612752 3.1111582 -4.4697433
H -2.4049373 1.9112993 -3.9081457
H -4.3095227 0.9659081 -2.6728669
H -4.0192315 0.0021972 3.9403724
H -0.5474898 -1.0329633 7.0611779
H -2.7320884 -0.5833660 5.9794533
H 4.5305031 1.4858262 -1.5366489
H 5.0739771 1.6560023 0.1331923
H 4.2845517 -0.6190594 0.6871477
H 5.2719629 -0.7341071 -0.7669287
H -4.3702317 -1.0044502 -0.0871487
H -5.3469865 0.3535907 -0.6542932
H -4.6778387 1.7185549 1.3066179
H -5.2965255 0.1692768 1.8920667
H 1.8803071 -1.2075806 6.9528742
H 4.0115032 -1.0670719 5.6835435
H 3.9821956 -0.4745747 3.2641545
H 1.8722699 -0.0378213 2.1099745
H -1.0070316 -5.6069611 -4.7289371
H -3.2153407 -4.4740016 -4.8260745
H -3.5061031 -2.2236000 -3.7870344
H -1.6414860 -1.1135065 -2.6782193
H 0.0115182 -0.3747638 -1.8592725
H 0.0609326 0.2434359 1.3749700

[**4**,2H$^+$]-I
Total energy: –1916.2885227085 au
C -1.3346496 -1.6034862 4.0329822
C -0.0556239 -2.0819098 3.6483651
C 0.8474858 -2.5445976 4.6668648
C 0.4349936 -2.4988357 6.0226112
C -0.8201921 -2.0229669 6.3698734
C -1.7100986 -1.5758422 5.3680632
C 2.1428784 -3.0518548 4.3069701
C 2.5476605 -3.1214644 3.0024455
C 1.6845883 -2.6721674 1.9510478
C 0.4045208 -2.1429419 2.2818288
C 2.0391771 -2.7616578 0.5850380
C 1.1976304 -2.3226033 -0.4277856
C -0.0425216 -1.7336405 -0.0754000
N -0.3782717 -1.6979340 1.2431054
C 1.5070822 -2.4863702 -1.8961022

C 0.2226221 -2.9007789 -2.6365114
C -0.8993036 -1.9166698 -2.3669011
C -0.9829753 -1.2548719 -1.1084700
C -1.8711060 -1.6658764 -3.3446108
C -2.9238567 -0.7860190 -3.1005942
C -2.9712990 -0.0906545 -1.8888871
C -1.9827040 -0.2513990 -0.8935917
O -3.9755447 0.8533931 -1.6955363
C -4.9676230 0.4895286 -0.7187012
O -4.3790479 -0.0316826 0.4863786
C -3.3078482 0.7067168 0.9805825
C -3.4702010 1.4128879 2.1760108
C -2.3799859 2.0805486 2.7306322
C -1.1434287 2.1137066 2.0723986
C -0.9957449 1.4463274 0.8227659
C -2.0678567 0.6475264 0.3077488
C 0.2699108 1.7087580 0.1083820
C 1.4500343 2.0604522 0.8103340
C 1.3613559 2.2011545 2.3106107
C 0.0242187 2.8719003 2.6737798
C 2.6089357 2.3048889 0.0863731
C 2.6408659 2.2484407 -1.3262655
C 1.4285930 1.9628990 -2.0168932
N 0.3166388 1.7019739 -1.2518573
C 3.8326400 2.4983174 -2.0809057
C 3.8039422 2.4723863 -3.4480207
C 2.5895837 2.2105486 -4.1703213
C 1.3667893 1.9543580 -3.4588003
C 0.1801957 1.7169728 -4.1993759
C 0.1955461 1.7257660 -5.5864174
C 1.3982363 1.9697306 -6.2860942
C 2.5723507 2.2082153 -5.5881008
H -3.6968195 -0.6094033 -3.8456874
H -1.8100281 -2.1813302 -4.3015048
H -0.0803558 -3.9085769 -2.3094251
H 0.4053840 -2.9606141 -3.7154858
H 2.2984728 -3.2314608 -2.0303433
H 1.8842273 -1.5387240 -2.3127783
H 3.0009073 -3.2024194 0.3245925
H 2.8027268 -3.3921357 5.1035729
H 3.5254588 -3.5183409 2.7373877
H -5.5233160 1.4142392 -0.5252513
H -4.4402468 1.4084556 2.6687926
H -2.4923966 2.6004775 3.6803778
H 0.0225598 3.9102472 2.3046508
H -0.0946908 2.9235157 3.7620522
H 2.2100836 2.7853622 2.6818449
H 1.4196921 1.2104552 2.7890144
H 3.5245751 2.5619479 0.6179731
H 4.7124371 2.6603376 -4.0181816
H 4.7537468 2.7094917 -1.5416489
H -5.6145613 -0.3153356 -1.0860564
H 3.5011856 2.4019412 -6.1226106
H 1.3994223 1.9744686 -7.3745087

H -0.7257178 1.5481221 -6.1374797
H -0.7710378 1.5397851 -3.7005002
H 1.1229076 -2.8494835 6.7905567
H -1.1246293 -1.9969724 7.4145431
H -2.6977721 -1.2117149 5.6434904
H -2.0567400 -1.2635270 3.2925616
H -0.5588873 1.5203084 -1.7420204
H -1.3051814 -1.3402892 1.4730233

[**5**,2H^{+}]-I
Total energy: −1988.9075446382 au
C 1.5929521 1.0659769 6.3326301
C 0.6935393 0.3245121 5.5252348
C 0.4540735 0.8213785 4.2085914
N 1.0276817 1.9463702 3.7077869
C 1.8654371 2.6150714 4.4987012
C 2.1787841 2.2106046 5.8186830
C -0.4528534 0.0898909 3.3637957
C -1.1114668 -1.0751136 3.8226126
C -0.8429408 -1.5432653 5.1525216
C 0.0283241 -0.8698131 5.9689326
C -2.0286856 -1.6903009 2.9362306
C -2.2683036 -1.1906881 1.6639354
C -1.5498963 -0.0430761 1.2136747
N -0.6953826 0.5383433 2.0930807
C -1.7774981 0.5164787 -0.1359050
C -3.0648505 0.2556238 -0.6935794
C -4.0488319 -0.6451020 0.0262580
C -3.3038323 -1.7850195 0.7411089
C -0.8239160 1.2915895 -0.8618230
C -1.2096017 1.8385355 -2.1148483
C -2.5068756 1.6424674 -2.6159153
C -3.4084994 0.8497569 -1.9096665
C 0.5700394 1.6163003 -0.3857986
C 1.6671894 0.7037539 -0.3830843
C 2.9410496 1.1126359 0.1130886
C 3.1214085 2.4427190 0.4982983
C 2.0749390 3.3613505 0.4638256
C 0.7962052 2.9472309 0.0564329
C 4.0833184 0.1210723 0.2135172
C 3.5489280 -1.2716154 0.5893097
C 2.5018966 -1.6825381 -0.4167989
C 1.6055596 -0.6813526 -0.8962043
C 2.4140294 -2.9736381 -0.9179549
C 1.4800790 -3.3254294 -1.9227767
C 0.6421044 -2.2968459 -2.4143360
N 0.7386957 -1.0416186 -1.8761093
C 1.3632622 -4.6407909 -2.4850148
C 0.4643548 -4.8957960 -3.4880557
C -0.3817716 -3.8587958 -4.0120577
C -0.2973858 -2.5378213 -3.4776228
C -1.3170394 -4.0649713 -5.0576501
C -2.0827632 -2.9999391 -5.5013349
C -1.9135178 -1.7288866 -4.9013901

N -1.0475986 -1.4938310 -3.9167663
O -0.3031165 3.7606942 0.0770685
C -0.1410579 5.1501339 0.4607039
O -0.2373153 2.5281250 -2.7854693
C -0.5705017 3.1652836 -4.0452202
H 0.3473303 3.6656390 -4.3627181
H -1.3706893 3.9042636 -3.9099593
H -0.8671018 2.4218232 -4.7963631
H 0.3827797 -5.8961759 -3.9105159
H 2.0073292 -5.4290543 -2.1005044
H 3.0930355 -3.7375745 -0.5408435
H 3.1079848 -1.2380633 1.5986966
H 4.3536706 -2.0145824 0.6136896
H 4.8069440 0.4677660 0.9601231
H 4.6209349 0.0582110 -0.7467640
H 4.0996692 2.7658305 0.8511244
H 2.2499671 4.3839558 0.7838801
H -1.1380449 5.5880263 0.3726015
H 0.5533122 5.6642140 -0.2162948
H 0.2112394 5.2342567 1.4969428
H -4.6257458 -0.0664232 0.7661140
H -4.7704277 -1.0504300 -0.6922698
H -3.9935010 -2.4158659 1.3126091
H -2.8152175 -2.4322371 -0.0051994
H -2.5722271 -2.5729468 3.2714968
H -1.3499463 -2.4414749 5.4993290
H 0.2256880 -1.2305955 6.9772832
H -4.3997490 0.6748095 -2.3252315
H -2.8054383 2.0764678 -3.5652833
H 1.8118637 0.7298913 7.3451046
H 2.8722301 2.8023849 6.4120879
H 2.3173227 3.5157707 4.0821530
H -1.4220863 -5.0543960 -5.5003001
H -2.8084580 -3.1242013 -6.3020585
H -2.5079755 -0.8808645 -5.2427388
H -0.1946055 1.3993126 1.8426897
H 0.1115641 -0.3473879 -2.2995216

[**5**,2H$^+$]-II
Total energy: –1988.9084429501 au
N 2.1948303 2.5569912 0.5109600
C 1.9229708 2.8192965 1.8166483
C 2.6592733 3.7464359 2.6133804
C 3.7283496 4.4257448 1.9763126
C 4.0028198 4.1589708 0.6453943
C 3.2100973 3.2139061 -0.0490375
C 2.3000933 3.9446096 3.9912372
C 1.2645572 3.2521459 4.5638405
C 0.5003841 2.3105271 3.7960546
C 0.8310125 2.1165596 2.4352196
C -0.5651006 1.5360976 4.3164422
C -1.2706530 0.6329267 3.5329251
C -0.9393898 0.4932304 2.1523040
N 0.1001497 1.2323089 1.6898833

C -2.3738495 -0.2307126 4.0931468
C -2.3064993 -1.6250409 3.4500626
C -2.3431695 -1.5112003 1.9391262
C -1.6785161 -0.4400519 1.2761201
C -3.0365733 -2.4541026 1.1773694
C -3.1165053 -2.3553602 -0.2097575
C -2.5010754 -1.2799826 -0.8714779
C -1.7592954 -0.3078822 -0.1452675
C -1.2589471 0.8890818 -0.9133967
C -0.2958575 0.8206868 -1.9680485
C -0.0195222 1.9671018 -2.7669017
C -0.6690702 3.1686530 -2.4763900
C -1.5999593 3.2639788 -1.4446711
C -1.9128957 2.1297757 -0.6774532
C 0.4593638 -0.4001673 -2.3200383
C 1.0081815 -0.5815399 -3.6243807
C 0.8360311 0.5285069 -4.6318318
C 0.9593003 1.8856346 -3.9211537
C 1.6925990 -1.7526047 -3.9204762
C 1.8920224 -2.7708668 -2.9564730
C 1.3726795 -2.5408101 -1.6617816
N 0.6866659 -1.3835493 -1.4133223
C 1.5554424 -3.4892897 -0.5960350
C 2.2620009 -4.6945901 -0.8866123
C 2.7698110 -4.9210300 -2.2123841
C 2.5967324 -3.9955013 -3.2090022
C 2.4241159 -5.6116984 0.1824264
C 1.9058705 -5.2984553 1.4278645
C 1.2262778 -4.0695291 1.6058566
N 1.0491408 -3.1821939 0.6275065
O -2.8603055 2.1329854 0.3100783
C -3.6042252 3.3533631 0.5671035
O -2.5879644 -1.0845929 -2.2231443
C -3.3736031 -2.0109109 -3.0190491
H -4.4214599 -2.0133623 -2.6931974
H -3.3040348 -1.6353944 -4.0425097
H -2.9593381 -3.0258842 -2.9655031
H -3.6811666 -3.0971733 -0.7661970
H -3.5393542 -3.2790017 1.6797894
H -3.1423735 -2.2452818 3.7928342
H -1.3815296 -2.1307579 3.7729402
H -2.2825918 -0.2902588 5.1834004
H -3.3505268 0.2294602 3.8722776
H -0.8318137 1.6483470 5.3668120
H 1.0043931 3.4033254 5.6096121
H 2.8741335 4.6586723 4.5798301
H -4.1623906 3.6649213 -0.3248546
H -2.9363309 4.1601460 0.8952110
H -4.2997809 3.0982273 1.3698564
H -2.0991340 4.2105425 -1.2616328
H -0.4545022 4.0495302 -3.0794843
H 1.9919009 2.0132822 -3.5566304
H 0.7687137 2.7045078 -4.6240393
H -0.1570852 0.4485142 -5.1027861

H 1.5815376 0.4269930 -5.4283282
H 2.0974713 -1.8912802 -4.9224065
H 2.9907978 -4.1723731 -4.2078066
H 3.3041975 -5.8485921 -2.4122471
H 4.3213771 5.1459733 2.5380520
H 4.8167343 4.6612993 0.1273763
H 3.4200789 2.9912531 -1.0955225
H 2.9533942 -6.5484939 0.0144329
H 2.0141056 -5.9791022 2.2693664
H 0.8185952 -3.8093529 2.5830651
H 0.3892433 1.1782879 0.7063152
H 0.3189432 -1.3148303 -0.4572024

[**5**,2H$^+$]-III
Total energy: –1988.9048545622 au
N 1.4527945 -1.9068370 2.5340080
C 1.2396589 -3.1370905 1.9969094
C 1.7409905 -4.3478801 2.5652864
C 2.4955668 -4.2310136 3.7595165
C 2.7085966 -2.9770833 4.3057425
C 2.1679478 -1.8423977 3.6563259
C 1.4716994 -5.6053716 1.9243633
C 0.7405093 -5.6691225 0.7675066
C 0.2165321 -4.4760159 0.1669296
C 0.4638198 -3.2290854 0.7876043
C -0.5316823 -4.4623669 -1.0337087
C -1.0219228 -3.2868257 -1.5833273
C -0.7865623 -2.0469484 -0.9152418
N -0.0445334 -2.0898689 0.2214080
C -1.3386827 -0.7808501 -1.4432192
C -1.6929213 -0.7849201 -2.8232299
C -1.4497105 -2.0122325 -3.6763825
C -1.7929940 -3.2792385 -2.8805583
C -2.2911186 0.3485535 -3.3788693
C -2.5540876 1.4833161 -2.6180538
C -2.2097048 1.5021498 -1.2569552
C -1.5830408 0.3798035 -0.6453802
C -1.4602378 0.4431318 0.8590084
C -0.5310339 1.2536959 1.5904560
C -0.7124021 1.4277475 2.9968496
C -1.7425345 0.7476658 3.6478123
C -2.6129211 -0.0957168 2.9627430
C -2.4874025 -0.2341973 1.5725420
C 0.6553102 1.9257231 1.0037859
C 1.7018075 2.4494727 1.8265721
C 1.6119146 2.3162493 3.3264666
C 0.1551494 2.3867665 3.7849769
C 2.8024009 3.0546199 1.2369220
C 2.9435508 3.1654606 -0.1650692
C 1.9060489 2.6222765 -0.9561536
N 0.8276736 2.0443811 -0.3403162
C 1.9621067 2.6712015 -2.3942956
C 3.0940073 3.2946450 -3.0024197
C 4.1347834 3.8464506 -2.1794504

C 4.0649134 3.7839449 -0.8124308
C 3.1201663 3.3288391 -4.4193820
C 2.0728537 2.7678609 -5.1307302
C 1.0002076 2.1730979 -4.4247331
N 0.9374477 2.1211757 -3.0951089
O -3.3374885 -0.9957991 0.8162050
C -4.4329619 -1.6862230 1.4718575
O -2.4587779 2.5669454 -0.4334366
C -3.1208595 3.7386608 -0.9785583
H -4.0579339 -2.4173791 2.1994655
H -4.9649794 -2.2018006 0.6689640
H -5.1048407 -0.9728967 1.9660470
H -3.3961468 -0.6156207 3.5058603
H -1.8682498 0.8794747 4.7213621
H 3.5888920 3.4540824 1.8764210
H 4.9879371 4.3172742 -2.6655977
H 4.8574529 4.2002152 -0.1937164
H -2.5242833 4.1906605 -1.7811621
H -4.1215118 3.4831956 -1.3497644
H -3.2016024 4.4339589 -0.1398754
H -3.0387804 2.3384868 -3.0789314
H -2.5707874 0.3388982 -4.4311770
H -0.7236769 -5.4060890 -1.5430680
H 1.8647188 -6.5143177 2.3773576
H 0.5437917 -6.6237912 0.2836632
H -0.2211971 3.4137532 3.6447957
H 0.0774587 2.1656593 4.8553673
H 2.0494032 1.3494218 3.6230235
H 2.2175440 3.0948595 3.8041785
H -0.3944524 -2.0536360 -3.9921640
H -2.0544718 -1.9554020 -4.5882877
H -2.8737670 -3.2982811 -2.6648997
H -1.5620773 -4.1848438 -3.4527476
H 2.8979239 -5.1255387 4.2326897
H 3.2828588 -2.8507153 5.2208325
H 2.3324351 -0.8502697 4.0769970
H 3.9609162 3.7942006 -4.9318564
H 2.0627066 2.7772758 -6.2184347
H 0.1679812 1.7275057 -4.9704739
H 0.1166332 1.6735365 -0.9845101
H 0.1752278 -1.2286277 0.7348548

[**5**,2H$^+$]-I′
Total energy: –1988.90812606 au
N -1.1513550 -1.5779062 -3.7943914
C -0.2900288 -2.5537880 -3.3751245
C -0.3849353 -3.8473391 -3.9607852
C -1.3747218 -4.0534527 -4.9513097
C -2.2233408 -3.0204276 -5.3379078
C -2.0917288 -1.7669392 -4.7375560
C 0.6885938 -2.2495980 -2.3652561
C 1.5782542 -3.2899887 -1.9735224
C 1.4701067 -4.5936948 -2.5666792
C 0.5261056 -4.8665406 -3.5219741

N 0.7228069 -0.9939396 -1.8555178
C 1.6467523 -0.6887944 -0.9358140
C 2.6160498 -1.6625140 -0.4961103
C 2.5571921 -2.9453580 -1.0120263
C 1.7174029 0.6982349 -0.3861156
C 2.9880417 1.1347843 0.0868303
C 4.1643915 0.1774688 0.1198389
C 3.6878140 -1.2401334 0.4778584
C 3.1459930 2.4566839 0.5109806
C 2.0781015 3.3519341 0.5266773
C 0.8081239 2.9162111 0.1219948
C 0.6077206 1.5909842 -0.3493874
C -0.7755722 1.2642918 -0.8495583
C -1.7530625 0.5171350 -0.1376421
C -3.0285290 0.2480872 -0.7289132
C -3.3230506 0.7921234 -1.9770249
C -2.3900645 1.5672730 -2.6753418
C -1.1201278 1.7921156 -2.1240207
C -4.0505393 -0.6026631 -0.0015468
C -3.3515964 -1.7393611 0.7616170
C -2.3146061 -1.1478599 1.6847832
C -1.5601813 -0.0268191 1.2239996
N -0.6990040 0.5425441 2.1049750
C -0.4847147 0.1092556 3.3858938
C -1.1812198 -1.0287888 3.8554321
C -2.1038818 -1.6324930 2.9675489
C -0.9405725 -1.4854816 5.1946833
C -0.0581168 -0.8268503 6.0108438
C 0.6443125 0.3423053 5.5578096
C 0.4319330 0.8279356 4.2320652
N 1.0395586 1.9314913 3.7248397
C 1.8879086 2.5869246 4.5153032
C 2.1787275 2.1903797 5.8427955
C 1.5557843 1.0691762 6.3649388
O -0.1346616 2.4933949 -2.7623591
C -0.4392501 3.1330760 -4.0256325
O -0.3141111 3.7069594 0.1666905
C -0.1760765 5.0886991 0.5786549
H 0.4758412 3.6589241 -4.3082708
H -1.2631041 3.8503463 -3.9147957
H -0.6906630 2.3910025 -4.7956503
H 0.4531634 -5.8558298 -3.9688172
H 2.1632750 -5.3691135 -2.2439104
H 3.2788543 -3.6960462 -0.6903982
H 3.2802353 -1.2388510 1.5024831
H 4.5170119 -1.9574822 0.4628166
H 4.9070385 0.5280380 0.8464642
H 4.6662384 0.1538611 -0.8618656
H 4.1243029 2.7928918 0.8528579
H 2.2331497 4.3680407 0.8764945
H -1.1808038 5.5121586 0.5043433
H 0.5065529 5.6314258 -0.0886100
H 0.1793864 5.1587757 1.6152721
H -4.6213687 0.0150575 0.7110002

H -4.7727516 -1.0086458 -0.7191258
H -4.0694063 -2.3316714 1.3400603
H -2.8683162 -2.4243244 0.0460482
H -2.6750830 -2.4949794 3.3097009
H -1.4766986 -2.3642060 5.5478481
H 0.1198298 -1.1801420 7.0254856
H -4.3004943 0.6100024 -2.4218177
H -2.6591460 1.9835895 -3.6419543
H 1.7547540 0.7399834 7.3838550
H 2.8834961 2.7696517 6.4354084
H 2.3671287 3.4702690 4.0918916
H -1.4661185 -5.0347211 -5.4136472
H -2.9850109 -3.1670297 -6.0974866
H -2.7134578 -0.9139274 -4.9902920
H -0.1584057 1.3739752 1.8367206
H -1.0360561 -0.6597618 -3.3476463

[**5**,2H$^+$]-II′
Total energy: –1988.9097887307 au
N 1.0761321 -3.0304443 0.8429320
C 1.5767504 -3.3815866 -0.3707688
C 2.2991348 -4.5890684 -0.6135208
C 2.4826130 -5.4567378 0.4927184
C 1.9693847 -5.0979238 1.7276943
C 1.2736766 -3.8718930 1.8568401
C 2.8023290 -4.8674807 -1.9306027
C 2.6113689 -3.9881473 -2.9646538
C 1.8919567 -2.7629224 -2.7615941
C 1.3729865 -2.4821021 -1.4756778
N 0.6722369 -1.3224663 -1.2784680
C 0.4297062 -0.3909235 -2.2350527
C 0.9876186 -0.6168480 -3.5281327
C 1.6862264 -1.7903409 -3.7713487
C 0.8102513 0.4583147 -4.5727342
C 0.9372139 1.8408685 -3.9082935
C -0.0468117 1.9627350 -2.7589862
C -0.3425858 0.8308525 -1.9401521
C -0.7014843 3.1676538 -2.5007791
C -1.6608636 3.2714778 -1.4910955
C -1.9984627 2.1459335 -0.7228121
C -1.3370323 0.9059984 -0.9235602
C -1.8554004 -0.2892801 -0.1683923
C -1.7408687 -0.4539053 1.2416591
C -2.4242211 -1.5162271 1.8939329
C -3.1645628 -2.4223418 1.1312021
C -3.2656037 -2.2975320 -0.2545384
C -2.6300453 -1.2278067 -0.9035547
C -0.9152868 0.4470455 2.0926124
C -1.2179649 0.5575808 3.4970748
C -2.3433498 -0.2692568 4.0686705
C -2.3539231 -1.6560940 3.4024711
N 0.1105184 1.1105282 1.5445626
C 0.8467815 1.9423918 2.3217650
C 0.6184978 2.1356902 3.7135526

C -0.4474402 1.3980357 4.2817644
C 1.9217471 2.6773241 1.7128246
C 2.7472172 3.5598634 2.4635412
C 2.4856483 3.7123230 3.8669784
C 1.4588920 3.0275199 4.4634020
C 3.7787544 4.2431201 1.7763390
C 3.9713747 4.0572613 0.4102536
C 3.1314021 3.1873743 -0.2869969
N 2.1535049 2.5400903 0.3724455
O -2.7255702 -1.0047676 -2.2556165
C -3.5232959 -1.9111875 -3.0572925
O -2.9753153 2.1567284 0.2371689
C -3.7059868 3.3851067 0.4769680
H -4.5717349 -1.9047459 -2.7314947
H -3.4512126 -1.5293142 -4.0786069
H -3.1243958 -2.9333072 -3.0140886
H -3.8591953 -3.0135926 -0.8147623
H -3.6835123 -3.2412035 1.6283849
H -3.2058729 -2.2465506 3.7601326
H -1.4431903 -2.2048014 3.6950130
H -2.2387726 -0.3496940 5.1572890
H -3.3045592 0.2337879 3.8714368
H -0.6558620 1.4932594 5.3472602
H 1.2640929 3.1508889 5.5277682
H 3.1172452 4.3849585 4.4433744
H -4.2415250 3.7071715 -0.4258664
H -3.0345741 4.1837114 0.8192826
H -4.4229319 3.1422668 1.2650027
H -2.1632186 4.2209107 -1.3317845
H -0.4776869 4.0403670 -3.1125816
H 1.9736425 1.9772065 -3.5569551
H 0.7482162 2.6353547 -4.6391767
H -0.1855631 0.3648064 -5.0349617
H 1.5519629 0.3300214 -5.3689597
H 2.0984129 -1.9689532 -4.7639918
H 3.0032580 -4.2019723 -3.9571325
H 3.3483983 -5.7956025 -2.0929548
H 4.4250251 4.9227315 2.3291663
H 4.7592477 4.5766491 -0.1265565
H 3.2220303 2.9950096 -1.3513991
H 3.0247727 -6.3919794 0.3606164
H 2.0938991 -5.7400400 2.5968547
H 0.8697385 -3.5749119 2.8252428
H 1.5180924 1.9009313 -0.1170195
H 0.3179237 -1.1878979 -0.3232815

[**5**,2H^{+}]-IIIa′
Total energy: –1988.9037893809 au
N 0.8447646 2.0881424 -3.2015963
C 1.9454868 2.5412345 -2.5493824
C 3.0880850 3.0883326 -3.2100766
C 3.0404923 3.1481987 -4.6256684
C 1.9147445 2.6864817 -5.2866813
C 0.8383627 2.1637395 -4.5311816

C 4.2104013 3.5461166 -2.4386928
C 4.2065766 3.4690610 -1.0708389
C 3.0771091 2.9262799 -0.3717883
C 1.9622796 2.4687884 -1.1105924
N 0.8819226 1.9552215 -0.4425346
C 0.7723155 1.8367563 0.9078419
C 1.8908215 2.2890306 1.6781137
C 3.0007583 2.8158182 1.0350945
C 1.8653590 2.1666563 3.1822228
C 0.4385463 2.3283734 3.7090272
C -0.5225421 1.4185795 2.9711439
C -0.4227859 1.2382834 1.5518997
C -1.5651675 0.8095543 3.6690101
C -2.5288113 0.0376433 3.0181730
C -2.4754416 -0.1027910 1.6250673
C -1.4326610 0.4941969 0.8668422
C -1.6050134 0.4186869 -0.6302981
C -1.3320780 -0.7227182 -1.4418101
C -1.6782984 -0.6961290 -2.8224616
C -2.3302691 0.4203591 -3.3515787
C -2.6361644 1.5295445 -2.5673709
C -2.2706770 1.5342119 -1.2138051
C -1.3448901 -1.8695306 -3.7211157
C -1.5990997 -3.1922410 -2.9881323
C -0.8698243 -3.2029523 -1.6673085
C -0.7381778 -1.9830732 -0.9053548
N -0.1145559 -1.9742200 0.2802761
C 0.3803101 -3.1334072 0.7792989
C 0.3250055 -4.3793885 0.0932970
C -0.3238804 -4.3699573 -1.1622527
C 0.9837838 -3.1215941 2.0851403
C 1.5375022 -4.2974671 2.6662858
C 1.4880769 -5.5214454 1.9179426
C 0.8984580 -5.5573732 0.6817645
C 2.0939589 -4.1960889 3.9631926
C 2.0869962 -2.9854719 4.6497372
C 1.5231035 -1.8617630 4.0446327
N 1.0039928 -1.9610769 2.8072646
O -2.5334750 2.5822935 -0.3653491
C -3.2159675 3.7491394 -0.8881673
O -3.4059253 -0.8024344 0.9037691
C -4.4951076 -1.4469872 1.6088666
H -4.1176612 -2.2015009 2.3118300
H -5.0914398 -1.9316128 0.8320144
H -5.1095917 -0.7106796 2.1437265
H -3.3233628 -0.4249896 3.5958350
H -1.6355787 0.9486805 4.7467420
H 3.8453031 3.1552208 1.6340255
H 5.0698560 3.9596732 -2.9643386
H 5.0583961 3.8172488 -0.4899119
H -2.6323642 4.2236860 -1.6880276
H -4.2153111 3.4862089 -1.2591905
H -3.3042397 4.4321816 -0.0397520
H -3.1555445 2.3751158 -3.0078216

H -2.6096663 0.4226296 -4.4045809
H -0.4015547 -5.2964459 -1.7308982
H 1.9171919 -6.4183878 2.3594932
H 0.8503752 -6.4914284 0.1238102
H 0.1205709 3.3754597 3.5737644
H 0.3972792 2.1238714 4.7849485
H 2.2658737 1.1787767 3.4654761
H 2.5390059 2.9095300 3.6246594
H -0.2857588 -1.8200661 -4.0244122
H -1.9410337 -1.8173595 -4.6397704
H -2.6807391 -3.3025747 -2.8010354
H -1.2909165 -4.0523793 -3.5949493
H 2.5247196 -5.0821129 4.4263942
H 2.5050192 -2.8967819 5.6477283
H 1.4706255 -0.8908867 4.5259931
H 3.8869872 3.5559838 -5.1763947
H 1.8457895 2.7192931 -6.3718345
H -0.0561355 1.7973656 -5.0359378
H 0.1126636 1.6299722 -1.0448188
H 0.5828413 -1.1463058 2.3453679

[**5**,2H$^+$]-IIIb′
Total energy: –1988.9055127077 au
N 0.9471453 -1.9753915 2.9513141
C 0.9474267 -3.1674891 2.3005849
C 1.4493403 -4.3784969 2.8704016
C 1.9678021 -4.3015603 4.1874385
C 1.9576748 -3.0861971 4.8498537
C 1.4351337 -1.9488030 4.1906103
C 1.4047187 -5.5996449 2.1154253
C 0.8754275 -5.6302244 0.8526322
C 0.3509572 -4.4383368 0.2503868
C 0.3974856 -3.2214325 0.9697181
C -0.2294315 -4.4033036 -1.0380337
C -0.7387917 -3.2370768 -1.5880392
C -0.6722385 -2.0177278 -0.8436905
N -0.1001843 -2.0836381 0.3884040
C -1.2323961 -0.7580360 -1.3870179
C -1.4930252 -0.7475008 -2.7967530
C -1.0801772 -1.9138415 -3.6697521
C -1.3743904 -3.2371651 -2.9566958
C -2.1346696 0.3502058 -3.3638434
C -2.5257325 1.4512711 -2.5925216
C -2.2439083 1.4667813 -1.2201166
C -1.5654796 0.3797627 -0.6002361
C -1.4090192 0.5034605 0.8940989
C -0.3778365 1.2424816 1.5491420
C -0.4608836 1.4484500 2.9571309
C -1.5370630 0.9206041 3.6726679
C -2.5347827 0.1708275 3.0514392
C -2.4733165 -0.0359885 1.6669563
C 0.8200021 1.8073512 0.8484892
C 1.9985681 2.1162832 1.6244353
C 1.9919573 1.9150462 3.1190122

C 0.6042032 2.2478811 3.6810489
C 3.1200249 2.6021312 0.9762458
C 3.1127738 2.8124871 -0.4207177
C 1.8978965 2.5022452 -1.0953029
N 0.7955701 2.0114553 -0.4756850
C 1.8498271 2.7400076 -2.5146753
C 2.9728169 3.2496985 -3.2270203
C 4.1778960 3.5335490 -2.5007128
C 4.2399475 3.3241572 -1.1486734
C 2.8381802 3.4545870 -4.6206361
C 1.6421884 3.1693730 -5.2719203
C 0.5661910 2.6757952 -4.5331447
N 0.7008562 2.4799277 -3.2087422
O -3.4149195 -0.7465775 0.9649601
C -4.5277616 -1.3283004 1.6878871
O -2.5909580 2.4978007 -0.3921086
C -3.3182371 3.6225084 -0.9442515
H -4.1769367 -2.0499270 2.4375783
H -5.1241759 -1.8429780 0.9305524
H -5.1325463 -0.5500972 2.1720178
H -3.3496709 -0.2361704 3.6424648
H -1.5925755 1.0867450 4.7479230
H 4.0190953 2.8339850 1.5469586
H 5.0348079 3.9222391 -3.0468639
H 5.1535786 3.5476000 -0.5996707
H -2.7289305 4.1338577 -1.7172024
H -4.2827088 3.3017561 -1.3595915
H -3.4836446 4.2979169 -0.1014019
H -3.0578546 2.2729947 -3.0629405
H -2.3551408 0.3447111 -4.4303806
H -0.2833133 -5.3266584 -1.6138527
H 1.7967080 -6.5078764 2.5707934
H 0.8372942 -6.5580427 0.2852669
H 0.4141136 3.3272807 3.5590764
H 0.5611968 2.0395304 4.7566916
H 2.2374412 0.8641208 3.3411184
H 2.7711287 2.5306456 3.5845765
H -0.0022253 -1.8539096 -3.8936496
H -1.6084796 -1.8665212 -4.6285764
H -2.4640191 -3.3647996 -2.8477284
H -1.0067589 -4.0929364 -3.5344867
H 2.3624490 -5.1977928 4.6640513
H 2.3425508 -2.9925949 5.8629312
H 1.4166852 -0.9869714 4.7035136
H 3.6856274 3.8426624 -5.1831174
H 1.5264956 3.3245133 -6.3402343
H -0.3985111 2.4353289 -4.9687205
H -0.0481067 -1.2386869 0.9729638
H -0.0903828 2.1206784 -2.6600536

[**5**,2H$^+$]-I''
Total energy: –1988.9076256761 au
N -1.1658045 -1.5789228 -3.7900283
C -0.2893684 -2.5482277 -3.3876840

C -0.3740598 -3.8377227 -3.9840635
C -1.3649051 -4.0445727 -4.9734060
C -2.2267747 -3.0172908 -5.3456657
C -2.1087976 -1.7698177 -4.7298484
C 0.6906804 -2.2438411 -2.3789698
C 1.5845193 -3.2821080 -1.9911929
C 1.4864350 -4.5816183 -2.5952143
C 0.5457565 -4.8531770 -3.5543191
N 0.7188055 -0.9908719 -1.8648086
C 1.6313704 -0.6860088 -0.9338661
C 2.5997787 -1.6600490 -0.4914311
C 2.5544327 -2.9384384 -1.0202864
C 1.6845309 0.6967447 -0.3716910
C 2.9421605 1.1300550 0.1495350
C 4.1262925 0.1821141 0.1773328
C 3.6573835 -1.2460777 0.5019672
C 3.0751231 2.4349981 0.6217837
C 1.9950174 3.3266471 0.6187888
C 0.7479454 2.8967379 0.1445768
C 0.5705175 1.5751849 -0.3477262
C -0.8031499 1.2385676 -0.8613243
C -1.7796791 0.4990389 -0.1454265
C -3.0509328 0.2371224 -0.7421060
C -3.3395832 0.7657977 -1.9994161
C -2.4001351 1.5290663 -2.7041353
C -1.1361821 1.7566212 -2.1423836
C -4.0824359 -0.5991577 -0.0085866
C -3.3934066 -1.7350472 0.7660472
C -2.3540646 -1.1476073 1.6887089
C -1.5655970 -0.0272361 1.2357568
N -0.6819616 0.5694613 2.0451340
C -0.5099132 0.0946309 3.3018977
C -1.2189240 -1.0190613 3.8347770
C -2.1616846 -1.6272509 2.9728315
C -0.9775058 -1.4492425 5.1838636
C -0.0744622 -0.8035646 5.9877493
C 0.6604090 0.3289778 5.4986928
C 0.4332651 0.7595877 4.1613841
N 1.1330923 1.8348861 3.6887777
C 2.0277821 2.5223915 4.4207426
C 2.2800461 2.1396359 5.7395851
C 1.6010063 1.0486374 6.2738398
O -0.1418580 2.4564096 -2.7794941
C -0.4423133 3.0969774 -4.0411429
O -0.3728684 3.6894504 0.1422456
C -0.2424067 5.0770907 0.5292567
H 0.4718723 3.6265384 -4.3212766
H -1.2684023 3.8127851 -3.9342524
H -0.6897061 2.3570684 -4.8150922
H 0.4802893 -5.8395433 -4.0088979
H 2.1835148 -5.3555480 -2.2770496
H 3.2788968 -3.6867100 -0.6990865
H 3.2376709 -1.2689396 1.5215236
H 4.4926731 -1.9564615 0.4828773

H 4.8599460 0.5243660 0.9171759
H 4.6370060 0.1800433 -0.8001804
H 4.0393908 2.7710753 1.0020158
H 2.1360372 4.3407785 0.9821425
H -1.2376824 5.5089248 0.3968257
H 0.4783017 5.6005251 -0.1133648
H 0.0614012 5.1710637 1.5811850
H -4.6489713 0.0279015 0.6998566
H -4.8087763 -1.0050267 -0.7230110
H -4.1174725 -2.3261713 1.3396271
H -2.9096997 -2.4222448 0.0517983
H -2.7494594 -2.4716471 3.3326297
H -1.5344828 -2.3048310 5.5631636
H 0.0995915 -1.1339791 7.0097163
H -4.3162685 0.5822214 -2.4462830
H -2.6614108 1.9364943 -3.6767926
H 1.7900908 0.7395608 7.3004463
H 3.0025832 2.6996119 6.3254621
H 2.5191903 3.3599292 3.9359276
H -1.4475283 -5.0220058 -5.4455378
H -2.9897055 -3.1643294 -6.1040534
H -2.7448544 -0.9226979 -4.9663304
H 0.9362987 2.1062716 2.7164610
H -1.0679105 -0.6649443 -3.3286836

[**5**,2H$^+$]-II''
Total energy: –1988.9076718217 au
N 0.8453927 -3.3537463 0.6483954
C 1.3838555 -3.5855691 -0.5862356
C 2.0110793 -4.8373982 -0.8389358
C 2.0426253 -5.7830829 0.2135271
C 1.4752576 -5.4918269 1.4508500
C 0.8720765 -4.2485156 1.6519901
C 2.5688641 -5.0714714 -2.1413538
C 2.4906837 -4.1115710 -3.1172142
C 1.8515248 -2.8479882 -2.8745192
C 1.2890471 -2.5678809 -1.5974462
N 0.6637025 -1.4085203 -1.2828773
C 0.5338852 -0.4628052 -2.2203541
C 1.1016588 -0.6318769 -3.5361794
C 1.7451965 -1.8191093 -3.8405333
C 0.9961706 0.4973048 -4.5321208
C 1.1473552 1.8425704 -3.8016335
C 0.1238480 1.9444799 -2.6846001
C -0.2027673 0.7950856 -1.9063090
C -0.5317242 3.1511675 -2.4319271
C -1.5274505 3.2455301 -1.4566213
C -1.8814676 2.1103834 -0.7136250
C -1.2107260 0.8732958 -0.9051708
C -1.7135909 -0.3100333 -0.1224095
C -1.5643519 -0.4550636 1.2851138
C -2.1805753 -1.5488966 1.9612940
C -2.9025448 -2.4900688 1.2246952
C -3.0639735 -2.3611139 -0.1569384

C -2.4936792 -1.2674236 -0.8236736
C -0.8041055 0.5144946 2.1252104
C -1.1332573 0.6367573 3.5249370
C -2.1867198 -0.2696522 4.1135943
C -2.0819177 -1.6638111 3.4721459
N 0.1705789 1.2482020 1.5760265
C 0.8172299 2.1623022 2.3377704
C 0.5446694 2.3857216 3.7166680
C -0.4579747 1.5697815 4.2929022
C 1.8354620 2.9660898 1.7177686
C 2.5520344 3.9587541 2.4426434
C 2.2454315 4.1435672 3.8333687
C 1.2796086 3.3844417 4.4422880
C 3.5250460 4.7117369 1.7432277
C 3.7658134 4.4846124 0.3911158
C 3.0383135 3.4978708 -0.2775926
N 2.1155035 2.7849813 0.3923670
O -2.6520910 -1.0443795 -2.1713852
C -3.4437447 -1.9797159 -2.9404225
O -2.8856636 2.1116063 0.2258891
C -3.6135664 3.3395405 0.4610513
H -4.4771260 -2.0218812 -2.5702106
H -3.4348946 -1.5930069 -3.9627089
H -3.0012127 -2.9850746 -2.9207777
H -3.6508397 -3.0982231 -0.6967608
H -3.3696871 -3.3299226 1.7380740
H -2.8758268 -2.3207237 3.8465714
H -1.1243053 -2.1218376 3.7719540
H -2.0751266 -0.3213523 5.2033913
H -3.1870292 0.1473791 3.9096239
H -0.6953775 1.6800485 5.3509728
H 1.0521043 3.5322042 5.4971738
H 2.7941478 4.9000749 4.3904847
H -4.1228496 3.6790612 -0.4511181
H -2.9478117 4.1313839 0.8308242
H -4.3544199 3.0937586 1.2261705
H -2.0340741 4.1932838 -1.3010083
H -0.2802644 4.0322462 -3.0215406
H 2.1738823 1.9182263 -3.4053225
H 1.0175382 2.6771441 -4.5007583
H 0.0101601 0.4627764 -5.0248954
H 1.7531151 0.3810200 -5.3172130
H 2.1764976 -1.9673378 -4.8305455
H 2.9153323 -4.2976574 -4.1027871
H 3.0516408 -6.0268996 -2.3360115
H 4.0876135 5.4771576 2.2750625
H 4.5085987 5.0574170 -0.1556112
H 3.1771996 3.2609517 -1.3276297
H 2.5170980 -6.7485513 0.0460803
H 1.4924671 -6.2104340 2.2645396
H 0.4167671 -3.9498153 2.5908163
H 1.5717955 2.0489859 -0.0751865
H 0.4148646 -2.4291589 0.7735848

[**5**,2H$^+$]-III"
Total energy: –1988.9029504894 au
N 0.6086623 2.5319104 -3.2308473
C 1.7945352 2.7233801 -2.5781184
C 2.9101234 3.2043831 -3.3213562
C 2.7283935 3.4582916 -4.7015463
C 1.4951482 3.2468271 -5.3097739
C 0.4295961 2.7753354 -4.5416342
C 4.1553125 3.4113230 -2.6377338
C 4.2621584 3.1581993 -1.2958264
C 3.1427587 2.6758054 -0.5367232
C 1.8888914 2.4409125 -1.1690406
N 0.7910492 1.9806811 -0.5208938
C 0.8582041 1.7278853 0.7931190
C 2.0765329 1.9712893 1.5309271
C 3.1929235 2.4279048 0.8533192
C 2.1142348 1.7468200 3.0230699
C 0.7574249 2.1162973 3.6372361
C -0.3603758 1.3661477 2.9382914
C -0.3351711 1.1899074 1.5194664
C -1.4378870 0.8728514 3.6776868
C -2.4956242 0.1961982 3.0643654
C -2.4831658 0.0194027 1.6747565
C -1.4097277 0.5077017 0.8835081
C -1.5799901 0.3721470 -0.6067761
C -1.1977524 -0.7454428 -1.3965106
C -1.4371142 -0.7172685 -2.8075392
C -2.1179129 0.3591200 -3.3722745
C -2.5632696 1.4347657 -2.5937260
C -2.2918436 1.4413158 -1.2199702
C -0.9504609 -1.8431853 -3.6976118
C -1.1760205 -3.1961912 -3.0166039
C -0.5655481 -3.1919414 -1.6371907
C -0.5925251 -1.9914246 -0.8311055
N -0.1158039 -1.9993842 0.4211145
C 0.3991460 -3.1433295 0.9334394
C 0.5263837 -4.3550967 0.1976740
C 0.0147078 -4.3361818 -1.1190197
C 0.8317071 -3.1543094 2.3062489
C 1.4113878 -4.3120257 2.9000883
C 1.5613077 -5.4936599 2.0989345
C 1.1259798 -5.5124834 0.8001421
C 1.7928118 -4.2392653 4.2605187
C 1.5901939 -3.0758785 4.9970154
C 1.0059938 -1.9707415 4.3773040
N 0.6583013 -2.0396803 3.0789142
O -2.6786655 2.4567477 -0.3821310
C -3.4646130 3.5428227 -0.9253208
O -3.4799275 -0.6277062 0.9874704
C -4.6262288 -1.1130485 1.7245299
H -4.3311482 -1.8702374 2.4638259
H -5.2805039 -1.5662541 0.9753397
H -5.1519207 -0.2889545 2.2259023
H -3.3146026 -0.1799561 3.6700491

H -1.4569442 1.0207976 4.7570311
H 4.1206499 2.6100959 1.3954950
H 5.0066159 3.7778846 -3.2074759
H 5.2063375 3.3246869 -0.7790738
H -2.9020289 4.0989590 -1.6878710
H -4.4061286 3.1736257 -1.3538090
H -3.6767041 4.1980482 -0.0766516
H -3.1223879 2.2411083 -3.0595855
H -2.3214374 0.3585683 -4.4426713
H 0.0651971 -5.2407542 -1.7248983
H 2.0126050 -6.3744338 2.5507402
H 1.2230129 -6.4173939 0.2019259
H 0.6015201 3.2030907 3.5358243
H 0.7442333 1.8964018 4.7116367
H 2.3416645 0.6872260 3.2288789
H 2.9275912 2.3312335 3.4700772
H 0.1254994 -1.7187481 -3.9061036
H -1.4670814 -1.8055951 -4.6639514
H -2.2598246 -3.3812341 -2.9250708
H -0.7603813 -4.0196258 -3.6102215
H 2.2422675 -5.1113073 4.7325615
H 1.8698768 -3.0111550 6.0440553
H 0.8002359 -1.0378410 4.8918993
H 3.5689334 3.8254327 -5.2878321
H 1.3430717 3.4407122 -6.3671611
H -0.5610227 2.5876179 -4.9433833
H -0.1750111 2.1826003 -2.6630719
H 0.2235736 -1.2327280 2.6137234

[**6**,2H^+]-I
Total energy: –1948.4137250015 au
N -1.3266202 -1.5604817 3.8327010
C -0.0965960 -2.1028999 3.6340042
C 0.7345785 -2.6002933 4.6817363
C 0.2218778 -2.5057003 6.0003756
C -1.0314660 -1.9522231 6.2012844
C -1.7734095 -1.4928223 5.0862324
C 2.0186291 -3.1647693 4.3663917
C 2.4632896 -3.2451561 3.0719085
C 1.6562668 -2.7597737 1.9890283
C 0.3962274 -2.1853139 2.2847250
C 2.0233983 -2.8487143 0.6236484
C 1.1994132 -2.3758480 -0.3888675
C -0.0372563 -1.7556394 -0.0503640
N -0.3777161 -1.7114323 1.2617732
C 1.5194207 -2.5369780 -1.8549842
C 0.2373146 -2.9336238 -2.6088858
C -0.8778604 -1.9398767 -2.3467270
C -0.9608084 -1.2690930 -1.0930211
C -1.8486091 -1.6936359 -3.3262626
C -2.8986208 -0.8103392 -3.0879249
C -2.9447547 -0.1066310 -1.8811901
C -1.9582385 -0.2617937 -0.8848746
O -3.9477942 0.8414395 -1.6955695

C -4.9396190 0.4867609 -0.7153109
O -4.3526008 -0.0259547 0.4939661
C -3.2790962 0.7158795 0.9804078
C -3.4413136 1.4367624 2.1668540
C -2.3518837 2.1105248 2.7133315
C -1.1153477 2.1334861 2.0554976
C -0.9682485 1.4508292 0.8141245
C -2.0410212 0.6474437 0.3077322
C 0.2873065 1.7203963 0.0873356
C 1.4735580 2.1021281 0.7773055
C 1.3908759 2.2427434 2.2778426
C 0.0500240 2.9032095 2.6461930
C 2.6237362 2.3807437 0.0509789
C 2.6450222 2.3280198 -1.3647290
C 1.4332053 1.9976408 -2.0187022
N 0.3277993 1.7060605 -1.2682945
C 3.7999519 2.6242448 -2.1635571
C 3.7284087 2.6011268 -3.5325922
C 2.5009522 2.2867770 -4.2116822
C 1.3314667 1.9802183 -3.4539215
N 0.1265352 1.6788089 -4.0052097
C 0.0434690 1.6721997 -5.3350966
C 1.1400449 1.9594273 -6.1835523
C 2.3687279 2.2660415 -5.6233132
H -3.6710790 -0.6365124 -3.8342728
H -1.7880746 -2.2152673 -4.2798224
H -0.0782713 -3.9404134 -2.2903144
H 0.4302140 -2.9904471 -3.6863920
H 2.3038950 -3.2898714 -1.9860081
H 1.9098509 -1.5906447 -2.2625557
H 2.9724605 -3.3160431 0.3634781
H 2.6367913 -3.5367768 5.1820488
H 3.4327887 -3.6823693 2.8420706
H -5.4942299 1.4137004 -0.5286757
H -4.4116983 1.4394598 2.6590361
H -2.4639881 2.6432261 3.6559976
H 0.0378342 3.9405551 2.2740522
H -0.0623373 2.9579735 3.7350997
H 2.2366852 2.8329809 2.6460318
H 1.4574828 1.2518182 2.7549914
H 3.5321256 2.6655572 0.5806948
H 4.6090492 2.8292822 -4.1310757
H 4.7312726 2.8732894 -1.6591033
H -5.5884277 -0.3193859 -1.0768723
H 3.2302860 2.4927634 -6.2495224
H 1.0043809 1.9372145 -7.2625291
H -0.9302407 1.4321649 -5.7631403
H 0.8155755 -2.8692730 6.8377451
H -1.4538594 -1.8657467 7.1998847
H -2.7634505 -1.0590684 5.2298376
H -0.5179681 1.5091595 -1.8167432
H -1.2896863 -1.3412448 1.5553129

[**6**,2H$^+$]-I′
Total energy: −1948.4166143627 au
C -4.5246527 4.0532157 -2.3876716
C -3.2410350 3.4877949 -2.5959781
C -2.5411501 3.0414483 -1.4350151
N -3.0315448 3.1339859 -0.1710408
C -4.2403357 3.6730471 -0.0179368
C -5.0230623 4.1455922 -1.0991364
C -1.2338804 2.4670307 -1.6138302
C -0.6384783 2.3632270 -2.8939182
C -1.3775546 2.8195274 -4.0364543
C -2.6303172 3.3573882 -3.8907485
N -0.5415488 2.0288845 -0.5190880
C 0.7077312 1.5030176 -0.5518743
C 1.3587908 1.4338284 -1.8179885
C 0.6787025 1.8435487 -2.9562239
C 2.7931965 0.9643669 -1.8383038
C 3.5341833 1.5639867 -0.6292192
C 2.8181139 1.2290846 0.6655822
C 1.3984341 1.1108927 0.6892934
C 0.7316891 0.7054317 1.8870303
C 1.5032456 0.6231163 3.0635950
C 2.8904922 0.7895160 3.0552416
C 3.5431907 1.0559962 1.8508760
O 0.8775031 0.2738854 4.2600023
C 0.0420371 1.3022155 4.8245316
O -0.8044674 1.9206423 3.8430902
C -1.4642990 1.0217321 3.0002839
C -2.8451753 0.8628722 3.1326418
C -3.5195306 0.0267975 2.2451513
C -2.8226544 -0.7049865 1.2734947
C -1.4092098 -0.5900645 1.1788795
C -0.7239439 0.3604499 1.9975428
C -0.7154063 -1.5657264 0.2914914
C -1.4332279 -2.1294271 -0.8209462
C -2.8710825 -1.7219779 -1.0288448
C -3.5723609 -1.6369026 0.3392056
N 0.5254226 -1.9622727 0.6025371
C 1.1280478 -2.8939901 -0.1749975
C 0.5254796 -3.4748963 -1.3283282
C -0.7959910 -3.0608091 -1.6220774
C 2.4470679 -3.3406226 0.1892756
C 3.1491524 -4.3040753 -0.5866574
C 2.5084245 -4.8443146 -1.7528789
C 1.2444151 -4.4471756 -2.1034421
C 4.4437425 -4.6826106 -0.1563354
C 4.9999532 -4.1336383 0.9945454
C 4.2679773 -3.2008123 1.7325093
N 3.0422744 -2.8387161 1.3136219
H -3.3744796 1.4104143 3.9097994
H -4.6012223 -0.0761602 2.3165985
H -3.6296702 -2.6451545 0.7810340
H -4.6046301 -1.2866814 0.2203392
H -3.3764689 -2.4347872 -1.6904332

H -2.9160094 -0.7376756 -1.5232548
H -1.3168541 -3.4954718 -2.4746907
H 3.0456296 -5.5812576 -2.3459099
H 0.7602319 -4.8707419 -2.9821199
H 0.6430069 2.1218828 5.2366436
H 3.4437651 0.6892801 3.9871727
H 4.6270670 1.1573602 1.8348425
H 3.5995938 2.6578386 -0.7474743
H 4.5635250 1.1897611 -0.5868949
H 3.2703218 1.2565532 -2.7799658
H 2.8299194 -0.1356016 -1.7856361
H 1.1738906 1.7818887 -3.9247876
H -3.1830821 3.7037045 -4.7627605
H -0.9189009 2.7347585 -5.0196052
H -0.5450122 0.7916420 5.5975563
H -5.1015627 4.4100988 -3.2395943
H -6.0028511 4.5764848 -0.9051581
H -4.6189912 3.7427773 1.0022374
H 5.0032411 -5.4153002 -0.7355354
H 5.9897272 -4.4194667 1.3363644
H 4.6288286 -2.7440190 2.6487577
H -1.0277839 2.1608283 0.3760239
H 2.4804125 -2.1724593 1.8549635

[**6**,2H^{+}]-I''
Total energy: –1948.4176390358 au
C -4.5063325 3.9974021 -2.3483256
C -3.2206759 3.4482427 -2.5720690
C -2.4814231 3.0070743 -1.4397091
N -3.0371618 3.1385888 -0.1971348
C -4.2553109 3.6646336 0.0223769
C -5.0212855 4.1040557 -1.0600411
C -1.1670920 2.4355247 -1.5714402
C -0.6084707 2.3487427 -2.8792504
C -1.3659695 2.7945302 -4.0152926
C -2.6241032 3.3198556 -3.8722125
N -0.5296795 2.0247245 -0.4495519
C 0.7027283 1.5116862 -0.5557728
C 1.3812423 1.4358878 -1.8234141
C 0.7100077 1.8394857 -2.9647629
C 2.8155578 0.9664428 -1.8448374
C 3.5587578 1.5462296 -0.6268300
C 2.8398806 1.1925653 0.6628062
C 1.4216493 1.0928727 0.6785900
C 0.7569741 0.6782956 1.8702647
C 1.5239472 0.5721654 3.0475684
C 2.9136968 0.7095479 3.0456658
C 3.5674868 0.9823381 1.8422668
O 0.8798576 0.2379943 4.2441737
C 0.0673558 1.2883219 4.7967239
O -0.7646467 1.9261321 3.8126707
C -1.4383367 1.0410675 2.9633757
C -2.8263358 0.9293632 3.0693633
C -3.5097896 0.0934825 2.1836612

C -2.8124341 -0.6834816 1.2483198
C -1.3953399 -0.5977167 1.1726614
C -0.7022874 0.3538902 1.9778329
C -0.7069479 -1.5829977 0.2945856
C -1.4194799 -2.1474635 -0.8220381
C -2.8579885 -1.7433452 -1.0352199
C -3.5624013 -1.6316489 0.3298812
N 0.5314570 -1.9824221 0.6101114
C 1.1418054 -2.9037138 -0.1721671
C 0.5487916 -3.4790844 -1.3326861
C -0.7753868 -3.0720711 -1.6263422
C 2.4631783 -3.3407268 0.1936147
C 3.1771594 -4.2932916 -0.5848502
C 2.5462066 -4.8290789 -1.7586454
C 1.2805455 -4.4383980 -2.1123707
C 4.4730914 -4.6623021 -0.1499249
C 5.0210540 -4.1114487 1.0044489
C 4.2784176 -3.1859426 1.7415170
N 3.0504824 -2.8351465 1.3202617
H -3.3544541 1.5026031 3.8291557
H -4.5951642 0.0205398 2.2385066
H -3.6211010 -2.6317730 0.7902163
H -4.5944336 -1.2837164 0.2012942
H -3.3638261 -2.4674564 -1.6846172
H -2.9007311 -0.7678648 -1.5472191
H -1.2918123 -3.5061556 -2.4822572
H 3.0926736 -5.5559542 -2.3558841
H 0.8058388 -4.8574148 -2.9984027
H 0.6850496 2.0968115 5.2073549
H 3.4660063 0.5893911 3.9759413
H 4.6536013 1.0624977 1.8238703
H 3.6252380 2.6418156 -0.7309043
H 4.5884908 1.1706382 -0.5918354
H 3.2985566 1.2635064 -2.7831810
H 2.8493254 -0.1347059 -1.8011394
H 1.2003435 1.7833936 -3.9364499
H -3.1901735 3.6553599 -4.7385800
H -0.9170238 2.7101548 -5.0039198
H -0.5351426 0.7974400 5.5711661
H -5.0918670 4.3414226 -3.1992037
H -6.0044960 4.5266233 -0.8767284
H -4.5835583 3.7275493 1.0551039
H 5.0407253 -5.3886937 -0.7290278
H 6.0127898 -4.3889367 1.3483113
H 4.6328996 -2.7246931 2.6579905
H 2.4823365 -2.1688515 1.8560925
H -2.4525433 2.8256060 0.5864939

7-I
Total energy: -1493.6049285102 au
C 1.9355707 1.3118505 -2.4221808
C 1.5252722 1.5749726 -1.0836195
C 2.5126228 2.0042097 -0.1285634
C 3.8682578 2.1362252 -0.5394301

C 4.2367536 1.8637000 -1.8459299
C 3.2614196 1.4525885 -2.7933191
C 2.0571109 2.3053394 1.1838964
C 0.7247336 2.1683523 1.5094108
C -0.1830894 1.6924452 0.4966279
N 0.2049080 1.4259346 -0.7511621
C 0.1505079 2.5497592 2.8528681
C -1.1554794 3.3371962 2.6356267
C -2.1306852 2.5553014 1.7679634
C -1.6354575 1.6306938 0.8102621
C -3.5140977 2.7964439 1.8947326
C -4.4449645 2.1651089 1.0680909
C -3.9433434 1.2153344 0.1845844
C -2.5805715 0.8427872 0.0845739
O -4.7417179 0.5097729 -0.7041055
C -3.8878436 -0.3730781 -1.3492396
C -2.5639226 -0.2876162 -0.8537537
C -1.6368389 -1.2772597 -1.3020132
C -2.0180470 -2.1098987 -2.3874628
C -3.3267972 -2.0642210 -2.9100825
C -4.3025391 -1.2274602 -2.3654695
C -1.0193731 -3.1043915 -2.9612005
C 0.4283644 -2.6137474 -2.7689681
C 0.6578227 -2.3168476 -1.3061872
C -0.3833844 -1.6301370 -0.5851695
C 1.7802717 -2.7170578 -0.6134786
C 1.8888987 -2.4752269 0.7832776
C 0.7788230 -1.8213003 1.4246311
N -0.3271808 -1.4113679 0.7289741
C 3.0093535 -2.8718361 1.5649786
C 3.0310297 -2.6418636 2.9301854
C 1.9299236 -2.0095040 3.5670392
C 0.8280417 -1.6082938 2.8321925
H -5.3306468 -1.2268158 -2.7212897
H -3.5898377 -2.7271808 -3.7336779
H -1.1385716 -4.0850153 -2.4696842
H -1.2329181 -3.2615002 -4.0265754
H 1.1488065 -3.3595605 -3.1276069
H 0.5780685 -1.7003051 -3.3678407
H 2.5875623 -3.2393460 -1.1296341
H -0.0221829 -1.1232809 3.3092155
H 1.9569993 -1.8406306 4.6428791
H 3.8916876 -2.9491527 3.5227559
H 3.8489898 -3.3637381 1.0729508
H -5.5089676 2.3865774 1.1171686
H -3.8581811 3.5216481 2.6314159
H -0.9211708 4.3055310 2.1614361
H -1.6291831 3.5676437 3.5988060
H 0.8756774 3.1389151 3.4281382
H -0.0725452 1.6431481 3.4391330
H 2.7707281 2.6631789 1.9279838
H 1.1794621 0.9983820 -3.1402788
H 3.5635691 1.2470870 -3.8195526
H 5.2763673 1.9675878 -2.1537798

H 4.6119335 2.4599186 0.1894571

[**7**,1H$^+$]-I
Total energy: –1494.0554759155 au
C 0.8439126 -1.5838660 2.8253811
C 0.8162241 -1.8147685 1.4343032
C 1.8994790 -2.4677423 0.7718927
C 3.0231687 -2.8605208 1.5472983
C 3.0505986 -2.6192293 2.9103171
C 1.9558710 -1.9849731 3.5500943
N -0.2681687 -1.4279122 0.6700258
C -0.3843850 -1.6223505 -0.6642075
C 0.6621206 -2.3197857 -1.3385086
C 1.7762814 -2.7158656 -0.6234860
C -1.6202999 -1.2481315 -1.3502173
C -1.9981325 -2.0773315 -2.4441225
C -1.0037283 -3.0651443 -3.0289506
C 0.4503227 -2.6107787 -2.8034364
C -3.3123218 -2.0331814 -2.9483604
C -4.2842055 -1.2089310 -2.3808881
C -3.8722605 -0.3616391 -1.3566146
C -2.5469298 -0.2684975 -0.8712861
C -2.5604373 0.8536685 0.0757607
C -3.9266146 1.2178315 0.1803674
O -4.7215965 0.5122184 -0.7068744
C -1.6128570 1.6400759 0.8087256
C -2.1142504 2.5653313 1.7619098
C -3.4993558 2.8048559 1.8823064
C -4.4290323 2.1700945 1.0597846
C -0.1578190 1.7068691 0.4967424
C 0.7456807 2.2109206 1.4983905
C 0.1739109 2.6093161 2.8385458
C -1.1482121 3.3666779 2.6198423
N 0.2333698 1.3970906 -0.7410600
C 1.5501300 1.5598213 -1.0835204
C 2.5329077 2.0256018 -0.1401754
C 2.0775804 2.3486113 1.1665992
C 3.8855945 2.1673971 -0.5578259
C 4.2523904 1.8753107 -1.8600171
C 3.2800910 1.4328875 -2.7967180
C 1.9583579 1.2765281 -2.4184698
H -5.3145950 -1.2087116 -2.7297164
H -3.5813939 -2.6867704 -3.7765262
H -1.1545402 -4.0575593 -2.5732784
H -1.2004114 -3.1815966 -4.1017214
H 1.1586866 -3.3742414 -3.1429208
H 0.6409724 -1.7004010 -3.3934063
H 2.5833393 -3.2414520 -1.1334931
H -0.0021188 -1.1047087 3.3145505
H 1.9871338 -1.8121674 4.6240520
H 3.9132664 -2.9218229 3.5004193
H 3.8555582 -3.3564484 1.0513923
H -5.4924756 2.3931259 1.1054536
H -3.8443288 3.5368446 2.6114501

H -0.9381400 4.3351915 2.1354227
H -1.6226089 3.5954430 3.5826712
H 0.8922519 3.2243441 3.3940456
H -0.0236490 1.7126496 3.4492335
H 2.7892374 2.7259606 1.9023003
H 1.2037552 0.9454858 -3.1301730
H 3.5812399 1.2178422 -3.8209400
H 5.2887778 1.9892801 -2.1743249
H 4.6264311 2.5166656 0.1613882
H -1.0537731 -0.9974126 1.1605731

[**7**,2H^+]-I
Total energy: –1494.4934609008 au
C 1.9657399 1.3222651 -2.4510701
C 1.5823964 1.5918965 -1.1214000
C 2.5399003 2.0087034 -0.1454631
C 3.8992229 2.1263011 -0.5425264
C 4.2711214 1.8477998 -1.8460602
C 3.3014763 1.4497122 -2.8012440
C 2.0727600 2.3163559 1.1607552
C 0.7389847 2.1925546 1.5078004
C -0.1894966 1.7251517 0.5325667
N 0.2633149 1.4702521 -0.7202730
C 0.1782530 2.5704844 2.8572424
C -1.1464790 3.3308590 2.6626276
C -2.1158636 2.5503654 1.7952020
C -1.6206807 1.6291536 0.8289736
C -3.4999752 2.7911197 1.9044153
C -4.4211742 2.1654834 1.0662619
C -3.9226196 1.2146664 0.1806514
C -2.5603356 0.8417227 0.0850028
O -4.7128473 0.5075087 -0.7011783
C -3.8680411 -0.3753966 -1.3405979
C -2.5440053 -0.2907174 -0.8469437
C -1.6159393 -1.2794493 -1.3131373
C -1.9932288 -2.1080200 -2.4078377
C -3.3064158 -2.0595453 -2.9167428
C -4.2780987 -1.2298332 -2.3598514
C -0.9999705 -3.1000986 -2.9826490
C 0.4529291 -2.6389103 -2.7641636
C 0.6674157 -2.3433256 -1.2995680
C -0.3840020 -1.6620667 -0.6198971
C 1.7878785 -2.7311321 -0.5862751
C 1.9113158 -2.4869129 0.8081434
C 0.8214951 -1.8497910 1.4785383
N -0.2696293 -1.4680958 0.7175490
C 3.0421903 -2.8728124 1.5771911
C 3.0706997 -2.6381919 2.9407229
C 1.9713635 -2.0179979 3.5871544
C 0.8511715 -1.6256283 2.8700923
H -5.3068157 -1.2297368 -2.7125200
H -3.5745687 -2.7167070 -3.7418416
H -1.1500785 -4.0861852 -2.5133589
H -1.1989691 -3.2302294 -4.0531551

H 1.1629003 -3.4013438 -3.1015135
H 0.6396226 -1.7326837 -3.3620694
H 2.5980046 -3.2478780 -1.1001542
H 0.0019429 -1.1601367 3.3667869
H 2.0055124 -1.8486771 4.6613657
H 3.9379960 -2.9350335 3.5265992
H 3.8771649 -3.3580338 1.0752907
H -5.4844563 2.3894147 1.1093973
H -3.8509492 3.5136615 2.6387847
H -0.9461462 4.3117465 2.2013679
H -1.6138614 3.5320956 3.6340081
H 0.9035468 3.1778070 3.4088786
H -0.0082167 1.6636906 3.4543118
H 2.7890330 2.6682369 1.9026906
H 1.2189139 1.0282948 -3.1862376
H 3.6085814 1.2426464 -3.8242856
H 5.3126795 1.9389636 -2.1467433
H 4.6353897 2.4429227 0.1939359
H -1.0748106 -1.0884517 1.2189401
H -0.4232421 1.2511856 -1.4444906

References

20. Guy, L.; Mosser, M.; Pitrat, D.; Mulatier, J.C.; Kukułka, M.; Srebro-Hooper, M.; Jeanneau, E.; Bensalah-Ledoux, A.; Baguenard, B.; Guy, S. Modulation of Chiroptical Properties in a Series of Helicene-like Compounds. *J. Org. Chem.* **2019**, *84*, 10870–10876. https://doi.org/10.1021/acs.joc.9b01465.
21. Bensalah-Ledoux, A.; Pitrat, D.; Reynaldo, T.; Srebro-Hooper, M.; Moore, B.; Autschbach, J.; Crassous, J.; Guy, S.; Guy, L. Large-Scale Synthesis of Helicene-Like Molecules for the Design of Enantiopure Thin Films with Strong Chiroptical Activity. *Chem. Eur. J.* **2016**, *22*, 3333–3346. https://doi.org/10.1002/chem.201504174.
37. Becke, A.D. Density-functional exchange-energy approximation with correct asymptotic behavior. *Phys. Rev. A* **1988**, *38*, 3098–3100. https://doi.org/10.1103/PhysRevA.38.3098.
38. Lee, C.; Yang, W.; Parr, R.G. Development of the Colle-Salvetti correlation-energy formula into a functional of the electron density. *Phys. Rev. B* **1988**, *37*, 785–789. https://doi.org/10.1103/PhysRevB.37.785.
39. Weigend, F.; Ahlrichs, R. Balanced basis sets of split valence, triple zeta valence and quadruple zeta valence quality for H to Rn: Design and assessment of accuracy. *Phys. Chem. Chem. Phys.* **2005**, *7*, 3297–3305. https://doi.org/10.1039/B508541A.
40. Klamt, A.; Schrüümann, G. COSMO: a new approach to dielectric screening in solvents with explicit expressions for the screening energy and its gradient. *J. Chem. Soc., Perkin Trans. 2* **1993**, pp. 799–805. https://doi.org/10.1039/p29930000799.
41. Klamt, A. Calculation of UV/Vis Spectra in Solution. *J. Phys. Chem.* **1996**, *100*, 3349–3353. https://doi.org/10.1021/jp950607f.
42. Ernzerhof, M.; Scuseria, G.E. Assessment of the Perdew–Burke–Ernzerhof exchange-correlation functional. *J. Chem. Phys.* **1999**, *110*, 5029–5036. https://doi.org/10.1063/1.478401.
43. Srebro, M.; Govind, N.; de Jong, W.; Autschbach, J. Optical Rotation calculated with Time-Dependent Density Functional Theory: The OR45 benchmark. *J. Phys. Chem. A* **2011**, *115*, 10930–10949. https://doi.org/10.1021/jp2055409.
44. Autschbach, J.; Srebro, M. Delocalization error and 'functional tuning' in Kohn-Sham calculations of molecular properties. *Acc. Chem. Res.* **2014**, *47*, 2592–2602. https://doi.org/10.1021/ar500171t.
45. Körzdörfer, T.; Brédas, J.L. Organic Electronic Materials: Recent Advances in the DFT Description of the Ground and Excited States Using Tuned Range-Separated Hybrid Functionals. *Acc. Chem. Res.* **2014**, *47*, 3284–3291. https://doi.org/10.1021/ar500021t.
46. Schäfer, A.; Horn, H.; Ahlrichs, R. Fully optimized contracted Gaussian basis sets for atoms Li to Kr. *J. Chem. Phys.* **1992**, *97*, 2571–2577. https://doi.org/10.1063/1.463096.
47. Eichkorn, K.; Weigend, F.; Treutler, O.; Ahlrichs, R. Auxiliary basis sets for main row atoms and transition metals and their use to approximate Coulomb potentials. *Theor. Chem. Acc.* **1997**, *97*, 119–124. https://doi.org/10.1007/s002140050244.
48. Tomasi, J.; Mennucci, B.; Cammi, R. Quantum Mechanical Continuum Solvation Models. *Chem. Rev.* **2005**, *105*, 2999–3094. https://doi.org/10.1021/cr9904009.
49. Cossi, M.; Barone, V. Time-dependent density functional theory for molecules in liquid solutions. *J. Chem. Phys.* **2001**, *115*, 4708–4717. https://doi.org/10.1063/1.1394921.
50. TURBOMOLE V7.1 2016, a development of University of Karlsruhe and Forschungszentrum Karlsruhe GmbH, 1989-2007, TURBOMOLE GmbH, since 2007; available from http://www.turbomole.com.

51. Balasubramani, S.G.; Chen, G.P.; Coriani, S.; Diedenhofen, M.; Frank, M.S.; Franzke, Y.J.; Furche, F.; Grotjahn, R.; Harding, M.E.; HÃ¤ttig, C.; et al. TURBOMOLE: Modular program suite for ab initio quantum-chemical and condensed-matter simulations. *The Journal of Chemical Physics* **2020**, *152*, 184107. https://doi.org/10.1063/5.0004635.
52. Frisch, M.J.; Trucks, G.W.; Schlegel, H.B.; Scuseria, G.E.; Robb, M.A.; Cheeseman, J.R.; Scalmani, G.; Barone, V.; Petersson, G.A.; Nakatsuji, H.; et al. Gaussian 16 Revision A.03, 2016. Gaussian Inc. Wallingford CT.
53. Dennington, R.; Keith, T.; Millam, J. GaussView Version 5. Semichem Inc. Shawnee Mission KS 2009.
54. Autschbach, J.; Ziegler, T.; Patchkovskii, S.; van Gisbergen, S.J.A.; Baerends, E.J. Chiroptical properties from time-dependent density functional theory. II. Optical rotations of small to medium sized organic molecules. *J. Chem. Phys.* **2002**, *117*, 581–592. https://doi.org/10.1063/1.1477925.

Disclaimer/Publisher's Note: The statements, opinions and data contained in all publications are solely those of the individual author(s) and contributor(s) and not of MDPI and/or the editor(s). MDPI and/or the editor(s) disclaim responsibility for any injury to people or property resulting from any ideas, methods, instructions or products referred to in the content.
